# Multi-strategy enterprise development optimizer for numerical optimization and constrained problems

**DOI:** 10.1038/s41598-025-93754-3

**Published:** 2025-03-27

**Authors:** Xinyu Cai, Weibin Wang, Yijiang Wang

**Affiliations:** 1 College of Business, Jiaxing University, Jiaxing, 314001 China; 2 School of Labor and Human Resources, Renmin University of China, Beijing, 100872 China

**Keywords:** Enterprise development optimizer, Restart strategy, Metaheuristic algorithms, Engineering optimization problems, CEC2017, CEC2022, Computational science, Computer science

## Abstract

Enterprise Development Optimizer (EDO) is a meta-heuristic algorithm inspired by the enterprise development process with strong global search capability. However, the analysis of the EDO algorithm shows that it suffers from the defects of rapidly decreasing population diversity and weak exploitation ability when dealing with complex optimization problems, while its algorithmic structure has room for further enhancement in the optimization process. In order to solve these challenges, this paper proposes a multi-strategy enterprise development optimizer called MSEDO based on basic EDO. A leader-based covariance learning strategy is proposed, aiming to strengthen the quality of search agents and alleviate the weak population diversity of the EDO algorithm in the later search stage through the guiding role of the dominant group and the modifying role of the leader. To dynamically improve the local exploitation capability of the EDO algorithm, a fitness and distance-based leader selection strategy is proposed. In addition, the structure of EDO algorithm is reconstructed and a diversity-based population restart strategy is presented. The strategy is utilized to assist the population to jump out of the local optimum when the population is stuck in search stagnation. Ablation experiments verify the effectiveness of the strategies of the MSEDO algorithm. The performance of the MSEDO algorithm is confirmed by comparing it with five different types of basic and improved metaheuristic algorithms. The experimental results of CEC2017 and CEC2022 show that MSEDO is effective in escaping from local optimums with its favorable exploitation and exploration capabilities. The experimental results of ten engineering constrained problems show that MSEDO has the ability to competently solve real-world complex optimization problems.

## Introduction

With the rapid advancement of artificial intelligence technology, optimization problems in engineering, medicine, economics, and other fields have grown increasingly complex, characterized by challenges such as high-dimensionality, nonlinearity, multimodality, and stringent constraints^1,2^. For instance, in intelligent manufacturing scenarios, multi-objective workshop scheduling necessitates coordinating the dynamic coupling of energy consumption, temporal costs, and resource allocation^3^. The UAV trajectory planning problem requires balancing length, time and various types of constraints^4^. In medical image analysis, tumor segmentation requires balancing high-dimensional feature spaces against noise interference^5^, while hyperparameter optimization for deep neural networks faces bottlenecks such as non-convexity, non-differentiability, and prohibitive computational costs^6^. Traditional optimization methods (e.g., gradient descent methods and integer programming), although stable under ideal conditions, struggle in real-world scenarios due to their dependence on precise mathematical models, differentiable assumptions, and convexity requirements. These limitations often lead to failure when addressing large-scale non-convex or discontinuous problems, as computational complexity escalates exponentially or solutions converge to local optima^7,8^. Research indicates that for typical NP-hard problems, the runtime of conventional deterministic algorithms increases drastically with dimensionality, making them impractical for real-time applications^9^. In this context, metaheuristic algorithms have emerged as critical tools for addressing complex optimization challenges, owing to their gradient-free operation, global search capabilities, and strong adaptability^10^.

**Fig. 1:**
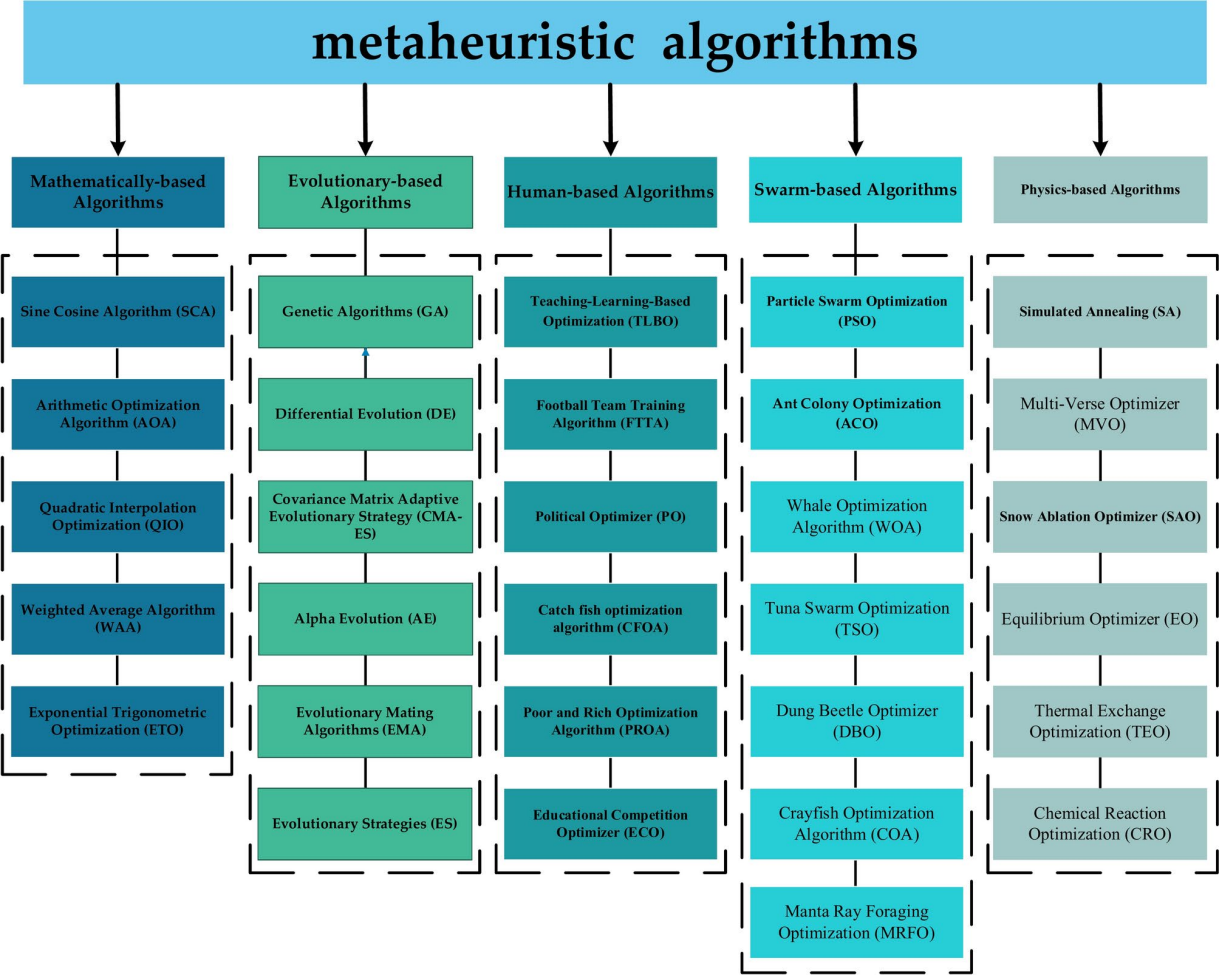
Classification of metaheuristic algorithm.

The core advantage of meta-heuristic algorithms lies in their ability to effectively balance global exploration and local exploitation by modeling natural evolution or group collaboration mechanisms and combining stochastic search and diversity strategies^11,12^. For example, Genetic Algorithm (GA) maintains population diversity through crossover and mutation operations to achieve extensive search in the solution space^13^. Particle Swarm Optimization (PSO) draws on the foraging behaviors of bird flocks to accelerate convergence by utilizing inter-individual information sharing^14^. Compared with traditional methods, the adaptability of metaheuristic algorithms is reflected in their low dependence on the problem structure. As an example, Simulated Annealing (SA), with its temperature decay mechanism, exhibits robustness in both discrete portfolio optimization and continuous parameter optimization by probabilistically accepting inferior solutions^15^. These results not only validate the engineering utility of metaheuristic algorithms, but also highlight their universal value in complex systems. Based on the source of inspiration and behavioral mechanisms, metaheuristic algorithms can be classified into the following five categories (as shown in Fig. 1).

Evolution-based algorithms: based on the principles of natural selection and genetic variation, they include Differential Evolution (DE)^16^, Covariance Matrix Adaptive Evolutionary Strategy (CMA-ES)^17^, Alpha Evolution (AE)^18^, Evolutionary Mating Algorithms (EMA)^19^, Evolutionary Strategies (ES)^20^. DE maintains population diversity through crossover and mutation operations, while CMA-ES utilizes adaptive covariance estimation to improve the efficiency of high-dimensional search. Swarm-based algorithms: simulate the collaborative behavior of groups of organisms, covering Ant Colony Optimization (ACO)^21^, Whale Optimization Algorithm (WOA)^22,23^, Tuna Swarm Optimization (TSO)^24^, Dung Beetle Optimizer (DBO)^25^, Crayfish Optimization Algorithm (COA)^26^, and Manta Ray Foraging Optimization (MRFO)^27^.ACO solves combinatorial optimization problems through pheromone positive feedback, DBO simulates dung beetle ball-rolling behavior to achieve efficient global search, while TSO adjusts the search direction based on the spiral foraging and parabolic foraging mechanisms of tuna groups. Mathematics-based algorithms: relying on mathematical theories and function models, such as Sine Cosine Algorithm (SCA)^28^, Arithmetic Optimization Algorithm (AOA)^29^, Quadratic Interpolation Optimization (QIO)^30^, Weighted Average Algorithm (WAA)^31^, and Exponential Trigonometric Optimization (ETO)^32^. SCA utilizes trigonometric periodicity to achieve a global search and QIO enhances local exploitation through generalized quadratic interpolation methods to enhance local exploitation. Physics-based algorithms: drawing on the laws of physics or chemical reaction mechanisms, including Multi-Verse Optimizer (MVO)^33^, Snow Ablation Optimization (SAO)^34^, Equilibrium Optimizer (EO)^35^, Thermal Exchange Optimization (TEO)^36^ and Chemical Reaction Optimization (CRO)^37^. MVO simulates the white hole-black hole interaction contraction solution space, TEO balances the exploration and exploitation through heat transfer mechanisms, and CRO adjusts the population evolution paths based on chemical reaction kinetics. Human-based algorithms: mapping social activities or cognitive processes, such as Teaching–Learning-Based Optimization (TLBO)^38^, Football Team Training Algorithm (FTTA)^39^, Political Optimizer (PO)^40^, Educational Competitive Optimizer (ECO)^41^, Escape Optimization Algorithm (EOA)^42^, Hiking Optimization Algorithm (HOA)^43^, and Social Network Search (SNS)^44^. TLBO accelerates convergence through “teacher” guidance and “student” mutual learning, while SNS simulates the information dissemination mechanism of social networks to enhance global search.

The Enterprise Development Optimizer (EDO), proposed by Truong in 2024, is a human-based meta-heuristic algorithm derived from the enterprise development process^45^. The algorithm performs the search for an optimization problem by constructing mathematical models of tasks, structures, technologies, and human interactions, and employs a switching mechanism to select which search model to follow. In the initial study, EDO demonstrated excellent search capabilities in classical test functions, CEC2020, CEC2022 compared to its comparison algorithms. However, the no free lunch theory suggests that there is no optimization technique that solves all problems perfectly^46^. Although EDO has demonstrated its potential as a metaheuristic algorithm, several limitations constrain its performance in solving complex optimization problems. A major problem is that all search methods move on their own base, ignoring the evolutionary tendencies of the population. This search approach reduces the convergence efficiency of the algorithm and weakens the diversity of the population. In addition, the existing search structure is unreasonable. Human-based search methods are mainly applied in the early stage. This approach can lead to the inability of the algorithm to balance the algorithm’s global and local search capabilities, as well as the difficulty of maintaining population diversity in the later stages of optimization. To further leverage the performance of EDO, it is essential to enhance it.

To address the deficiencies of EDO, this paper proposes a multi-strategy enterprise development optimizer with three optimization techniques to enhance the basic EDO algorithm. Firstly, we propose leader-based covariance learning strategy (LCL) and fitness and distance-based leader selection strategy (FLS) to address the problems of insufficient exploitation efficiency and weak population diversity. LCL allows the search agents to change the search position guided by the dominant group to enrich the population diversity while using the optimal individual to accelerate the convergence of the algorithm. FLS dynamically adjusts the local search capability of the EDO algorithm. Moreover, this paper proposes a diversity-based population restart strategy (DPR) to restart the process when the algorithm is stalled, assisting the algorithm to escape from local optima.

In the experimental section, ablation experiments based on the CEC 2017 and CEC 2022 test suites confirm the effectiveness of proposed improved strategies. Comparison results with five different types of basic and improved metaheuristic algorithms validate the performance of MSEDO. Numerical analysis, stability analysis, convergence analysis, Wilcoxon rank sum test, Friedman test and Kruskal Wallis test illustrate the superiority of the approach in this paper. The results for engineering design optimization problems further demonstrate the potential of MSEDO to solve real-world optimization problems. The main contributions of this paper are as follows.

1. The leader-based covariance learning strategy is proposed to allows the search agents to change the search position guided by the dominant group to enrich the population diversity while using the optimal individual to accelerate the convergence of the algorithm.
2. The introduction of fitness and distance-based leader selection strategy is to dynamically adjust the local search capability of the EDO algorithm.
3. The diversity-based population restart strategy is used to restart the process when the algorithm is stalled, assisting the algorithm to escape from local optima.
4. MSEDO is tested on 29 functions of CEC2017 and 12 functions of CEC2022 with different dimensions and 10 engineering design optimization problems. The effectiveness of different strategies are also discussed.

The paper is organized as follows: section "Enterprise development optimizer (EDO)" exhibits the structure and search strategies of the EDO algorithm. Three improvement mechanisms are proposed to enhance the MSEDO algorithm in Sect. "Multi-strategy enterprise development optimizer (MSEDO)": leader-based covariance learning strategy, fitness and distance-based leader selection strategy and diversity-based population restart strategy. Section "Experimental results and discussion" presents the results of ablation experiments based on the three improvement strategies. The superior performance of the MSEDO algorithm is verified by validating it under different dimensional problems in the CEC2017 and CEC2022 test sets, and the results are evaluated and comprehensively analyzed using a multitude of methods. Experimental results for engineering design optimization problems are also shown in this section. Finally, Sect. "Conclusions" reviews the work of this paper and provides an outlook for future work.

## Enterprise development optimizer (EDO)

The EDO algorithm is a meta-heuristic algorithm designed based on the enterprise development process. The optimization process of the EDO algorithm consists of four elements: task, structure, technology and people. Through these four elements, the EDO algorithm utilizes a mechanism for switching activities to smoothly transition from exploration to exploitation. This process is mathematically modeled as an optimization paradigm designed to determine the best solution while adhering to specific constraints. The mathematical model of the EDO algorithm is defined below.

### Initialization

The EDO algorithm randomly generates $NP$ individuals within the boundaries of the problem space in the initial phase. Each individual represents a solution $X_{i}$, denoted as follows.

1 $$X_{i} = lb + r_{1} \times \left( {ub - lb} \right),i = 1,2,...,NP$$

where $lb$ and $ub$ represent the upper and lower bounds about the solution of the problem to be solved. $r_{1}$ is a random number obeying a uniform distribution.

### Task

In the task phase, EDO assumes that tasks have different forms in business process management, and in order to model task activities, the worst activity is replaced using Eq. (2).

2 $$X_{worst}^{{}} = lb + r_{2} \times \left( {ub - lb} \right)$$

where $X_{worst}^{{}}$ is the individual with the worst fitness value among the candidate solutions, i.e., the one with the largest fitness (e.g., solving the minimization problem). $r_{2}$ is a random number obeying a uniform distribution.

### Structure

In the structure phase, the EDO algorithm assumes that each solution is influenced by the other candidate solutions as well as the best solution in the population. Equation (3) models this new structure.

3 $$X_{i}^{new} = X_{i}^{now} + r_{3} \times (X_{best}^{now} - X_{c}^{now} )$$

4 $$X_{c}^{now} = \frac{{X_{rand1}^{now} + X_{rand2}^{now} + \ldots + X_{randm}^{now} }}{m}$$

where $X_{i}^{new}$ is the new individual obtained after the updating of individual $X_{i}^{now}$. $r_{3}$ is a random number with a range between -1 and 1. $X_{best}^{now}$ is the best solution in the current population. $X_{c}^{now}$ is the average position of the other agents $X_{rand1}^{now}$, $X_{rand2}^{now}$ , …, $X_{randm}^{now}$, which are randomly selected from the population. m is the number of randomly selected individuals, which takes the value of 3 in the paper.

### Technology

EDO algorithm believes that companies need to increase exploration and development as a way to obtain the technology they need to grow. Therefore, EDO achieves a balance between exploitation and exploration in the technology phase by learning from the best and random solutions, as shown in Eq. (5).

5 $$X_{i}^{new} = X_{i}^{now} + r_{4} \times \left( {X_{best}^{now} - X_{i}^{now} } \right) + r_{5} \times \left( {X_{best}^{now} - X_{rand1}^{now} } \right)$$

where $r_{4}$ and $r_{5}$ are random numbers obeying a uniform distribution.

### People

In EDO, it is assumed that each human characteristic is a dimension, and Eq. (6) describes how human activity can be modeled by influencing one of the human characteristics.

6 $$X_{i,j}^{new} = X_{i,j}^{now} + r_{6} \times (X_{best,j}^{now} - X_{c,j}^{now} )$$

7 $$X_{c,j}^{now} = \frac{{X_{rand1,j}^{now} + X_{rand2,j}^{now} + \ldots + X_{randm,j}^{now} }}{m}$$

where $j$ denotes the $j{\text{th}}$ dimension of an individual.$r_{6}$ is are random numbers obeying a uniform distribution.

### Mechanism of switching activities

EDO assumes that only one step is utilized for each population updating. When $rand < 0.1$, EDO executes the task step. The EDO algorithm selects the structure, technology, and people steps, respectively, in case $c\left( {FEs} \right)$ takes the corresponding values 1, 2, and 3. $c\left( {FEs} \right)$ is calculated as shown below.

8 $$c\left( {FEs} \right) = 3 \times \left( {1 - \frac{{r_{7} \times FEs}}{{FEs_{\max } }}} \right)$$

where $FEs$ denotes the current number of evaluations and $FEs_{\max }$ denotes the maximum number of function evaluations.$r_{7}$ is are random numbers obeying a uniform distribution. Algorithm 1 presents the pseudo-code of the EDO algorithm.

**Algorithm 1:**
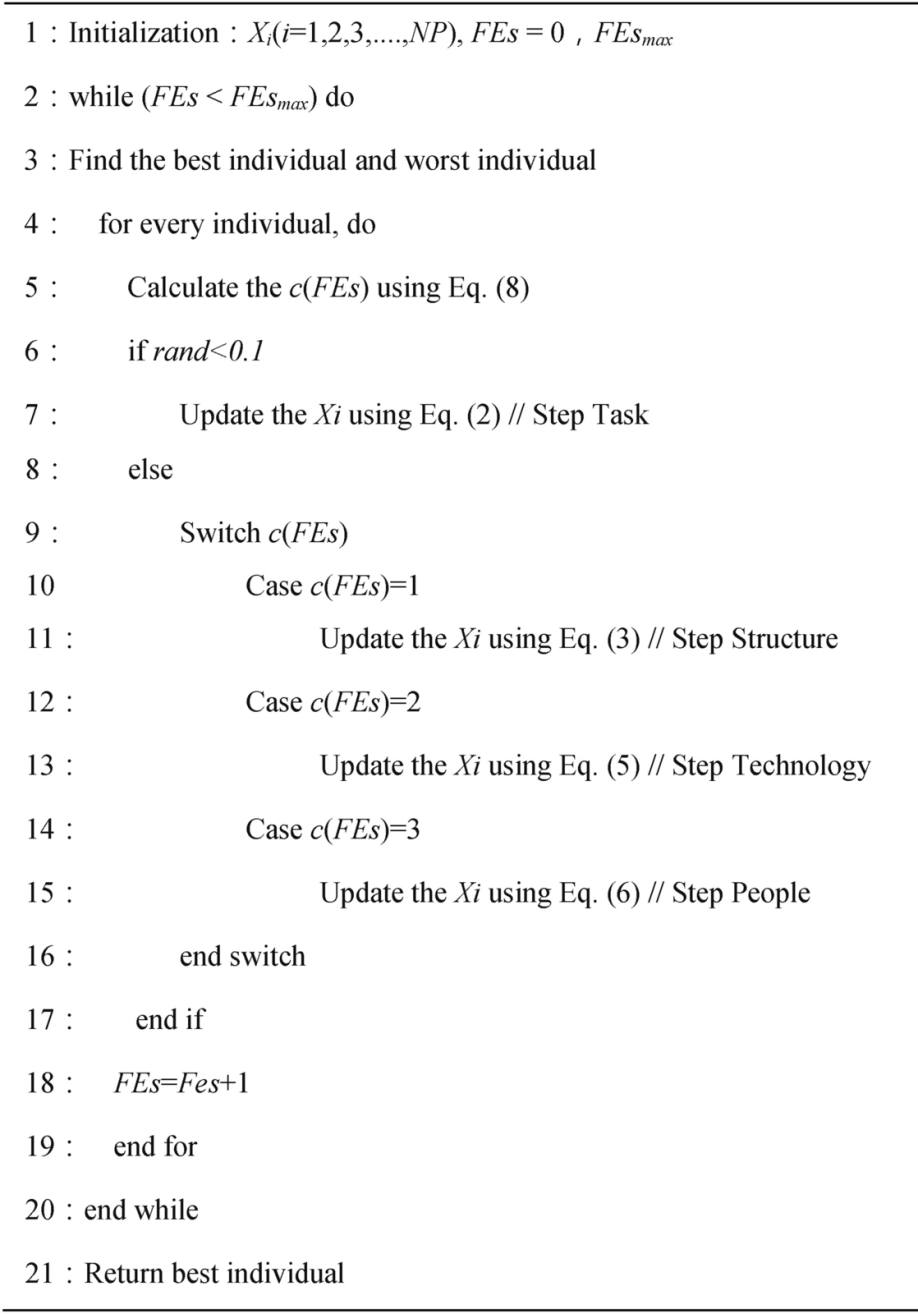
Enterprise development optimizer (EDO).

## Multi-strategy enterprise development optimizer (MSEDO)

### Motivation

The motivation to modify the basic EDO algorithm mainly stems from the bottleneck of its performance when confronting complex optimization problems, which mainly includes: the failure to leverage the information of the dominant population during the optimization process, which leads to the reduction of population quality. Insufficient exploitation efficiency as well as weakened population diversity in the later stages of optimization undermine the performance of the algorithm in different optimization scenarios. In addition, the algorithm structure has room for further enhancement and lacks a mechanism to get rid of the local optimum. Therefore, by introducing leader-based covariance learning strategy, fitness and distance-based leader selection strategy and diversity-based population restart strategy, it can improve the defect of insufficient exploitation efficiency, strengthen the population quality and enrich the population diversity, and help the algorithm to jump out of the local optimum smoothly.

### Leader-based covariance learning strategy (LCL)

The EDO algorithm replaces the worst individual in its task phase by randomly generating new individual in the problem space. This approach is rather blind and ignores the evolutionary trend of the whole population. This is detrimental to the quality of the population, while weakening the efficiency of the overall population exploitation. Therefore, this paper proposes a leader-based covariance learning strategy (LCL). The strategy incorporates three information: the best individual, the dominant population and itself. The optimal individual can modify the evolutionary direction of the population and increase the convergence efficiency. The dominant group represents the evolutionary direction of the population and can improve the quality of the population. The agent itself serves as a base point to expand the search. Overall, the strategy successfully strengthens the performance of the EDO algorithm with the guidance of three information. In addition, the task step occupies ten percent of the process in the whole searching process, and this strategy is mainly used to improve the quality of the population and accelerate the convergence speed. Therefore, in this paper, the judgment condition is modified to $rand < \frac{FEs}{{FEs_{\max } }}$ . In the pre-optimization phase, more other three steps are executed and in the post-optimization phase, more LCL strategies are executed to strengthen the population and accelerate the convergence through the bootstrapping effect of LCL strategy the details of LCL strategy are as follows.

9 $$X_{i}^{new} = \frac{{\left( {X_{best}^{now} + X_{mean} + X_{i}^{now} } \right)}}{3} + y,y\sim N\left( {0,Cov} \right)$$

10 $$Cov = \frac{1}{{NP_{d} }}\sum\limits_{i = 1}^{{NP_{d} }} {\left( {X_{i}^{d} - X_{mean} } \right) \times \left( {X_{i}^{d} - X_{mean} } \right)^{T} }$$

11 $$X_{mean} = \sum\limits_{i = 1}^{{NP_{d} }} {\omega_{i} \times X_{i}^{d} }$$

12 $$\omega_{i} = \frac{{\ln {(}NP_{d} { + 0}{\text{.5) - }}\ln {(}i{)}}}{{\sum\limits_{i = 1}^{{NP_{d} }} {{(}\ln {(}NP_{d} + 0.5{) - }\ln {(}i{))}} }}$$

$NP_{d}$ is the number of dominant groups and $X_{i}^{d}$ is the $i{\text{th}}$ individual of the dominant group. $X_{mean}$ is the weighted average position of the dominant group.

### Fitness and distance-based leader selection strategy (FLS)

By analyzing Eq. (8), we can learn that the structure step is more often executed in the later phases in the optimization. It is well known that metaheuristic algorithms need to search as much as possible a vast region in the early stage and as deeply as possible a localized region in the later stage to perform well. That is to say the EDO algorithm needs to have a strong local development capability in the late stage of optimization. However, the EDO algorithm searches around itself in the structural stage, which is not conducive to the precise development of EDO in the later stages and greatly weakens the convergence efficiency. Therefore, this paper proposes a Fitness and distance-based leader selection strategy (FLS). This strategy can dynamically adjust the development capability of EDO algorithms, which is realized by a roulette selection mechanism. In FLS, we select the leader based on the fitness and distance scores of each individual. Due to the roulette mechanism, the higher the score, the higher the probability that an individual will be selected as the leader. This mechanism effectively ensures a strong convergence ability, and at the same time has the probability to expand the search range with some global exploration ability. This capability meets the needs of the structural phase, i.e., strong exploitation and some degree of exploration. The details of the FLS are represented as follows.

13 $$Score_{i} = \omega_{1} \times Fit_{i} + \left( {1 - \omega_{1} } \right) \times Dis_{i}$$

14 $$X_{i}^{new} = X_{FLS} + r_{3} \times (X_{FLS} - X_{c}^{now} )$$

where $Fit_{i}$ is the score of the $i{\text{th}}$ individual. ω_1_ is the weight coefficient, which has a value of 0.5 in the paper. fit is the fitness value of the $i{\text{th}}$ individual. $Dis_{i}$ is the distance between the $i{\text{th}}$ individual and the optimal individual. $X_{FLS}$ is the leader selected through a score-based roulette selection mechanism.

### Diversity-based population restart strategy (DPR)

As the search proceeds, the population inevitably clusters around an optimal individual or multiple locally optimal individuals, which leads to a decrease in population diversity. When there is a severe lack of population diversity, the search of the population stagnates and fails to move towards other promising regions. Therefore, there is a need to determine if the algorithm is stagnant and to take steps to avoid stagnation. In addition, the three steps of EDO other than the task step are determined by the value of ct. The people step focuses more on the exchange of dimensions, an approach that greatly enriches population diversity but is not conducive to algorithmic convergence. However, the people step is more often executed in the pre-optimization phase, which seriously affects the comprehensive search of the problem space by the population. Therefore, we reconstruct the structure of EDO and propose a diversity-based population restart strategy (DPR). At first, we remove the people step within the normal selection framework. Then, we combine a diversity judgment technique with the people step. That is, when the algorithm is stalled, EDO employs the human step to update the population position. The details of DPR are represented as follows.

15 $$V_{\lim } = \sqrt {\prod\limits_{j = 1}^{D} {\left| {ub_{j} - lb_{j} } \right|} }$$

16 $$V_{pop} = \sqrt {\prod\limits_{j = 1}^{D} {\left| {\left( {ub_{{x_{j} }} - lb_{{x_{j} }} } \right)/2} \right|} }$$

17 $$nVOL = \sqrt {V_{pop} /V_{\lim } }$$

where $ub_{{x_{j} }}$ and $lb_{{x_{j} }}$ are the upper and lower boundaries of the $j^{th}$ dimensional position of the current population. After obtaining the boundaries of the search space and the spatial distribution of the population, we then need to record the number of times each individual fails to get better after updating. We introduce a $Count$ to record whether each individual becomes better at each update. If the individual does not get better, $Count$ is added to 1. When $nVOL < 0.01$ and $Count > 2 \times D$ are satisfied, MSEDO considers it stagnant and will perform the human step to update the stagnant individual.

### Implementation steps of the MSEDO

The proposed MSEDO algorithm is obtained by augmenting the basic EDO algorithm with the three improvement strategies mentioned above. To elucidate the proposed MSEDO, pseudo-code is provided in Algorithm 2.

**Algorithm 2:**
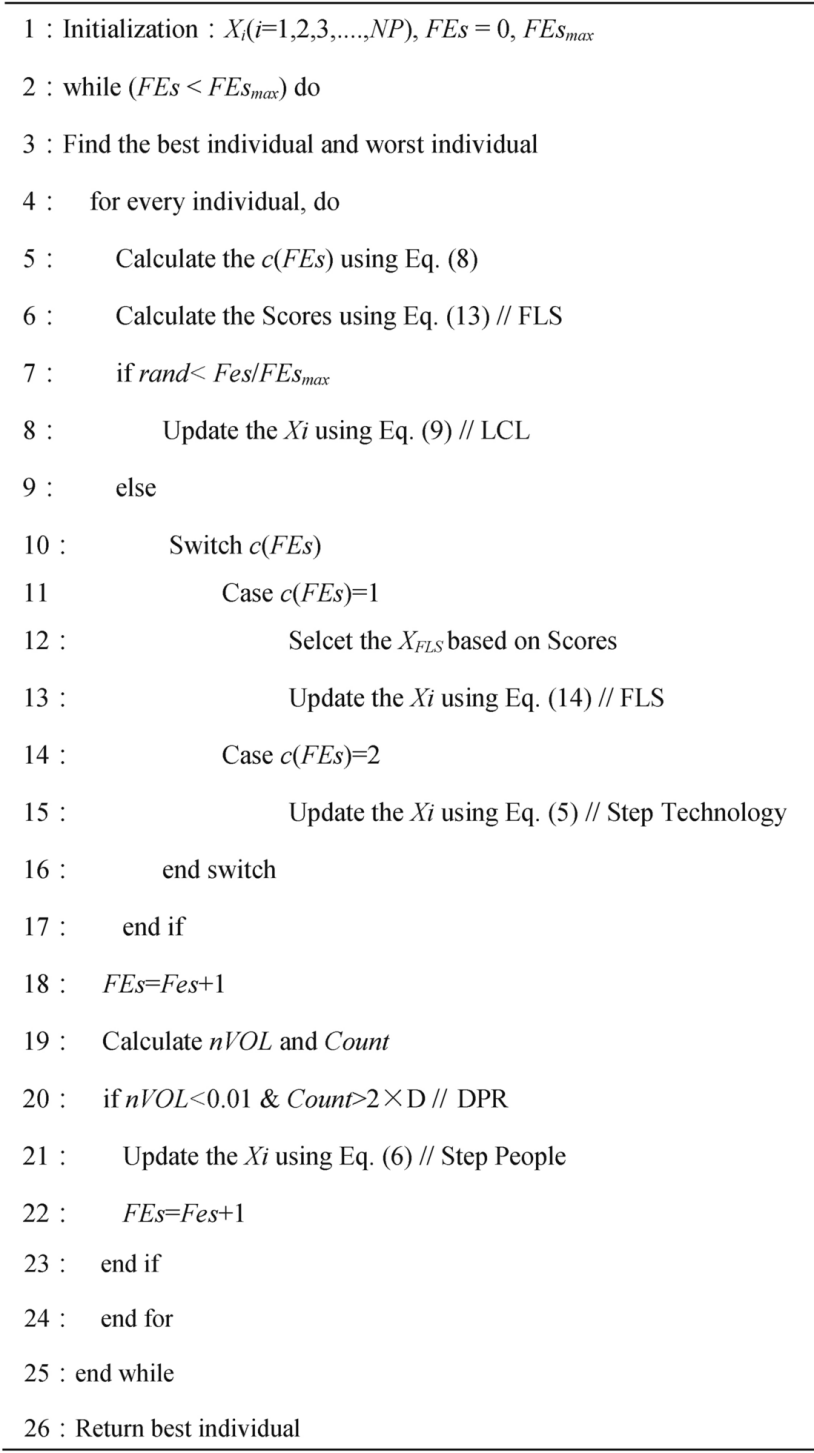
Multi strategy enterprise development optimizer (MSEDO).

### Computational time complexity

Time complexity is an important metric for evaluating the performance of an algorithm. In this paper, the population is assumed to be $NP$, the number of iterations is $T$, and the problem dimension is $D$. For the basic EDO algorithm, the time complexity of population initialization is $O\left( {NP \times D} \right)$. Since EDO chooses one step to update the individuals in each iteration, the time complexity of the EDO position updating strategy is $O\left( {T \times NP \times D} \right)$. Therefore, the time complexity of EDO is the sum of the time complexity of the initialization and the position updating strategy, which is $O\left( {NP \times D + T \times NP \times D} \right)$. Removing the low-order terms, the EDO algorithm’s time complexity is eventually $O\left( {T \times NP \times D} \right)$.

For the proposed MSEDO algorithm, LCL strategy is adopted to replace the task step and FLS strategy is adopted to replace the structure step. These new position updating methods do not increase extra computation and therefore do not affect the time complexity. For DPR, we assume that $NP_{r}$ individuals in each iteration will perform the DPR strategy, then the time complexity of the DPR strategy is $O\left( {T \times NP_{r} \times D} \right)$. Therefore, the total time complexity of the MSEDO algorithm is $O\left( {NP \times D + T \times NP \times D + T \times NP_{r} \times D} \right)$. Removing the low-order terms, the MSEDO algorithm’s time complexity is eventually $O\left( {T \times \left( {NP + NP_{r} } \right) \times D} \right)$.

We can learn that MSEDO will evaluate the fitness of more Nr individuals at each iteration compared to EDO. Therefore, the time complexity of MSEDO is higher than the basic EDO. To ensure fairness, the maximum number of function evaluations is employed as the stopping criterion for solving the problem in all subsequent experiments.

## Experimental results and discussion

In this section, we perform numerical experiments on MSEDO, EDO, and selected comparison algorithms using 41 functions from the CEC2017 test suite and the CEC2022 test suite, as well as 10 engineering constrained optimization problems. The results of the experiments are presented in four subsections. Sect. "Parameter setting and experimental environment" provides the experimental setup and the parameter settings of the comparison algorithms. Section "Effective analysis of each strategy" evaluates the impact of the different improvement strategies on the MSEDO algorithm. Sections "Comparison with basic algorithms" and "Comparison with improved algorithms" show the comparative results of MSEDO with the basic algorithm and the improved algorithms, respectively. Section "Comparison using constrained engineering optimization" summarizes the results of the MSEDO algorithm obtained for the engineering constrained optimization problems. More detailed information about the CEC2017 test suite and the CEC2022 test suite can be obtained in Table S1-S2 in the Supplementary file^47,48^.

### Parameter setting and experimental environment

The software and hardware specifications utilized in this paper are shown in Table 1. The MSEDO algorithm is developed based on MATLAB R2023. All experiments were conducted on a laptop equipped with AMD R9 7945HX, 2.5Ghz, 32 GB.

**Table 1:** Platform specifications utilized for experiments.

Hardware	
CPU	AMD R9 7945HX (2.5 GHz)
RAM	32 GB
Operating system	64-bit Windows 11

Software	
Programming language	MATLAB R2023

To comprehensively evaluate the performance of MSEDO, five different types of meta-heuristic algorithms are selected to participate in the experiments, including (1) Physics-based algorithms: RIME^49^, IRIME^50^. (2) Human-based algorithms: ECO, EMTLBO^51^. (3) Swarm-based algorithms: MRFO, EOSMA^52^. (4) Mathematics-based algorithms: QIO, MTVSCA^53^. (5) Evolution-based algorithms: AE, APSM-jSO^54^. The detailed parameter settings of the comparison algorithms are displayed in Table 2. In the experiments, the maximum number of function evaluations for each group experiment is 3000 times the number of dimensions. Specifically, for the 10/30/50/100 dimensions of CEC2017, the corresponding $FEs_{\max }$ is set to 30,000, 90,000, 150,000, and 300,000, respectively. For the 10/20 dimensions of CEC2022, the corresponding $FEs_{\max }$ is set to 30,000, and 60,000, respectively. All algorithms were tested in each of the above six cases for each function are solved 30 times to ensure robust statistical validity. In this paper, we use three nonparametric statistical test methods including Wilcoxon rank sum test, Friedman test and Kruskal Wallis test to analyze the experimental results obtained from MSEDO and comparison algorithms from a statistical point of view.

**Table 2:** Parameters setting of MSEDO and other competing algorithms.

Algorithm	Parameters setting
MSEDO	$m = 3,\omega_{1} = 0.5$
EDO	$m = 3$
RIME^49^	$W = 5$
IRIME^50^	$W = 5,F_{1} = 1,Cr_{1} = 0.1,F_{2} = 0.8,Cr_{2} = 0.2,F_{3} = 1,Cr_{1} = 0.9$
ECO^41^	$H = 0.5,G_{1} = 0.2,G_{2} = 0.1$
EMTLBO^51^	$p_{ini} = 0,swit_{ini} = 0,\alpha = 0.8$
MRFO^27^	$S = 2$
EOSMA^52^	$V = 1,a_{1} = 2,a_{2} = 1,GP = 0.5,z = 0.6,q = 0.2$
QIO^30^	$\alpha_{1} = 0.7,\alpha_{2} = 0.15,w = 3$
MTVSCA^53^	$\lambda = 0.25,n = 20,a = 2$
AE^18^	$w = 4$
APSM-jSO^54^	$k = 3,F = 0.3,CR = 0.8,H = 6$

### Effective analysis of each strategy

In our work, three modification techniques are integrated into the basic EDO to boost its optimization performance. Two experiments were designed to investigate the impact of the optimization techniques on the basic EDO. Three EDO variants integrating a single modification strategy were designed to evaluate the impact of LCL, FLS and DPR on the performance of MSEDO. Three EDO variants inheriting two modification strategies were designed to explore the impact of different strategies on each other. Table 3 shows the details of MSEDO and its six variants named MSEDO-1, MSEDO-2, MSEDO-3, MSEDO-12, MSEDO-13, MSEDO-23. The parameter settings for the six MSEDO variants are the same as those for MSEDO, and the parameter settings for the basic EDO algorithm are the same as in the original work, as shown in Table 2. Details of the results obtained by MSEDO, MSEDO-1, MSEDO-2, MSEDO-3, MSEDO-12, MSEDO-13, MSEDO-23 and EDO for the CEC2017 test suite and CEC2022 test suite are provided in the paper.

**Table 3:** Details of MSEDO and six MSEDO variants.

Strategy	MSEDO-1	MSEDO-2	MSEDO-3	MSEDO-12	MSEDO-13	MSEDO-23	MSEDO
LCL	Yes	No	No	Yes	Yes	No	Yes
FLS	No	Yes	No	Yes	No	Yes	Yes
DPR	No	No	Yes	No	Yes	Yes	Yes

**Fig. 2:**
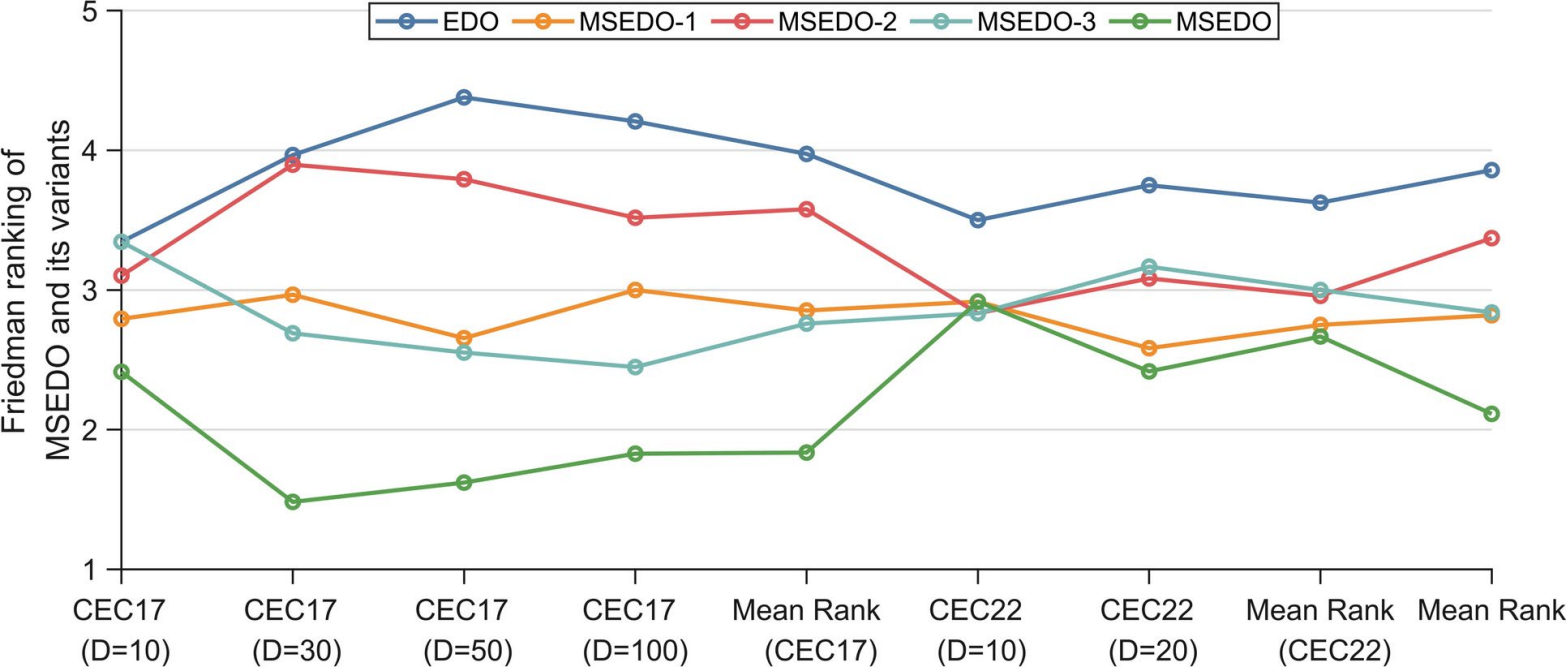
The ranking of MSEDO and its variants derived from the Friedman test (single strategy).

**Fig. 3:**
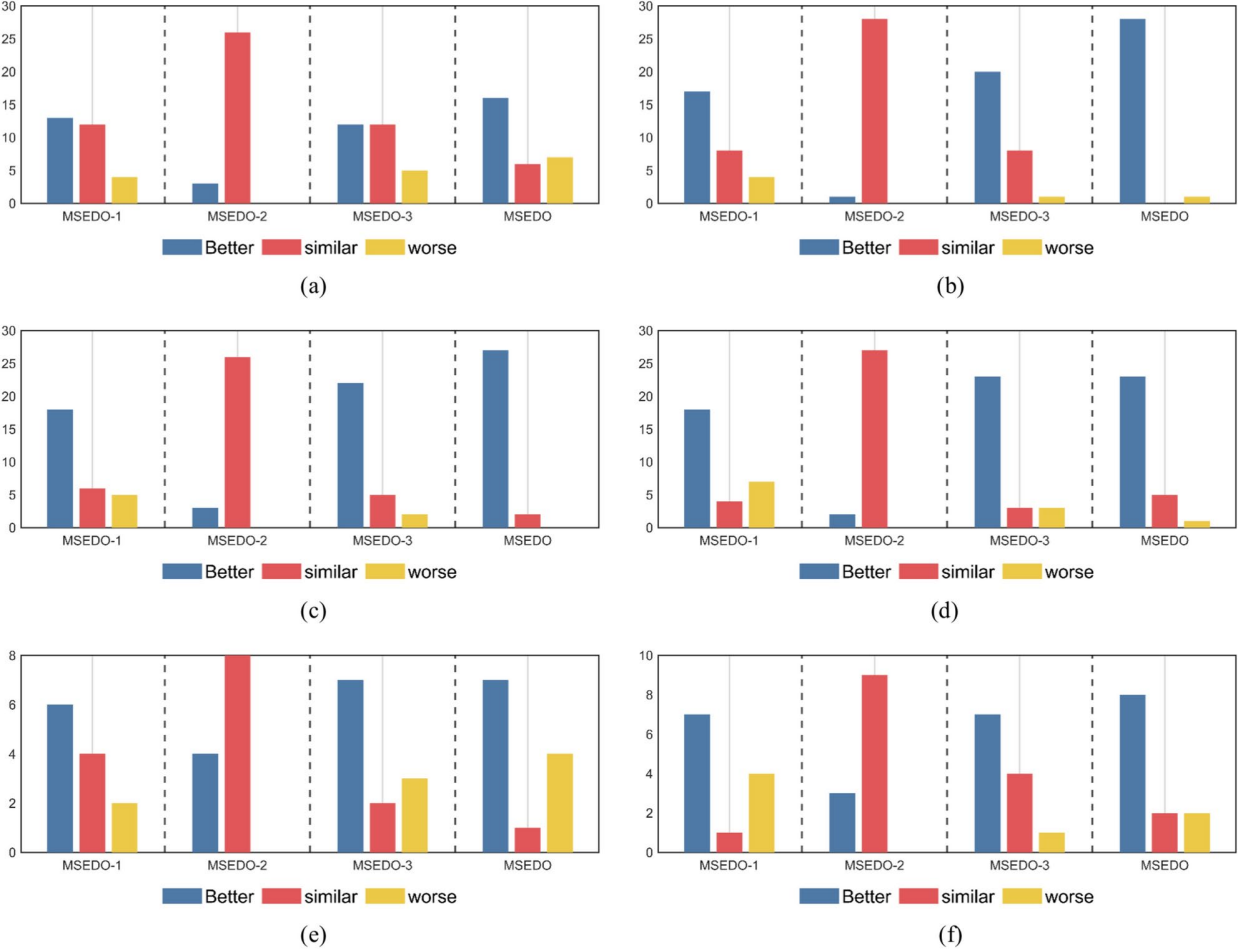
The visualization of Wilcoxon rank sum test results of MSEDO and its variants (single strategy).

Figure 2 illustrates the Friedman test results for MSEDO and EDO as well as the three MSEDO variants that inherit a single improved strategy. According to Fig. 2, MSEDO is worse than MSEDO-2 and MSEDO-3 on CEC2022 (D = 10) and ranks the same as MSEDO-1. Although MSEDO, which inherits the three improved strategies, is not the best in this case, the performance difference with the three variants is not significant, which can be supported by the Wilcoxon rank sum test results in Fig. 3. According to Fig. 3, MSEDO and the three variants obtained more + 's than − 's on CEC2022 (D = 10). The reason for MSEDO’s poorer ranking is that it loses to basic EDO more often, which explains why MSEDO obtains a poorer Friedman ranking in this case. For all other cases, MSEDO achieved the highest Friedman rankings and the most “ + ” signs. This indicates that MSEDO has the best overall performance. In addition, we can observe that MSEDO-1 is the best of the three variants for low-dimensional problems, and MSEDO-3 is more powerful in solving high-latitude problems. This suggests that the LCL mechanism enhances the ability of MSEDO to solve the low-latitude problem, leading the population evolution through the bootstrapping effect of the dominant group. The DPR mechanism, as a stagnant perturbation technique, plays a greater role in the high-dimensional problem, which is in line with our motivation for improvement. For MSEDO-2, we can find that it does not have any loss to EDO, that is, it does not receive a “ − ” sign. Although the improvement of FLS to EDO is minimal, its performance on EDO is not significantly impaired on all functions.

To further investigate the impact between the different improvement strategies. Table 4 summarizes the results of the Friedman test between MSEDO and the variants that integrate the two improvement strategies. According to Table 4, MSEDO integrating 3 strategies receives the highest Friedman scores in all cases. The 3 MSEDO variants integrating 2 strategies also ranked higher than the basic EDO. This indicates that the improvement strategies proposed in this paper do not restrict each other and jointly enhance the search capability of MSEDO.

**Table 4:** The Friedman ranking of MSEDO and its variants (two strategies).

Test suite	Dimension	EDO	MSEDO-12	MSEDO-13	MSEDO-23	MSEDO
CEC 2017	10	3.034	3.034	3.345	3.448	2.138
	30	4.724	2.000	3.276	3.207	1.793
	50	4.690	2.345	3.310	2.931	1.724
	100	4.276	3.276	3.000	2.793	1.655
Mean rank		4.181	2.664	3.233	3.095	1.828
CEC 2022	10	3.167	2.833	3.167	3.250	2.583
	20	3.500	2.667	3.250	3.333	2.250
Mean rank		3.333	2.750	3.208	3.292	2.417
Total mean rank		3.898	2.693	3.225	3.160	2.024

### Comparison with basic algorithms

**Fig. 4:**
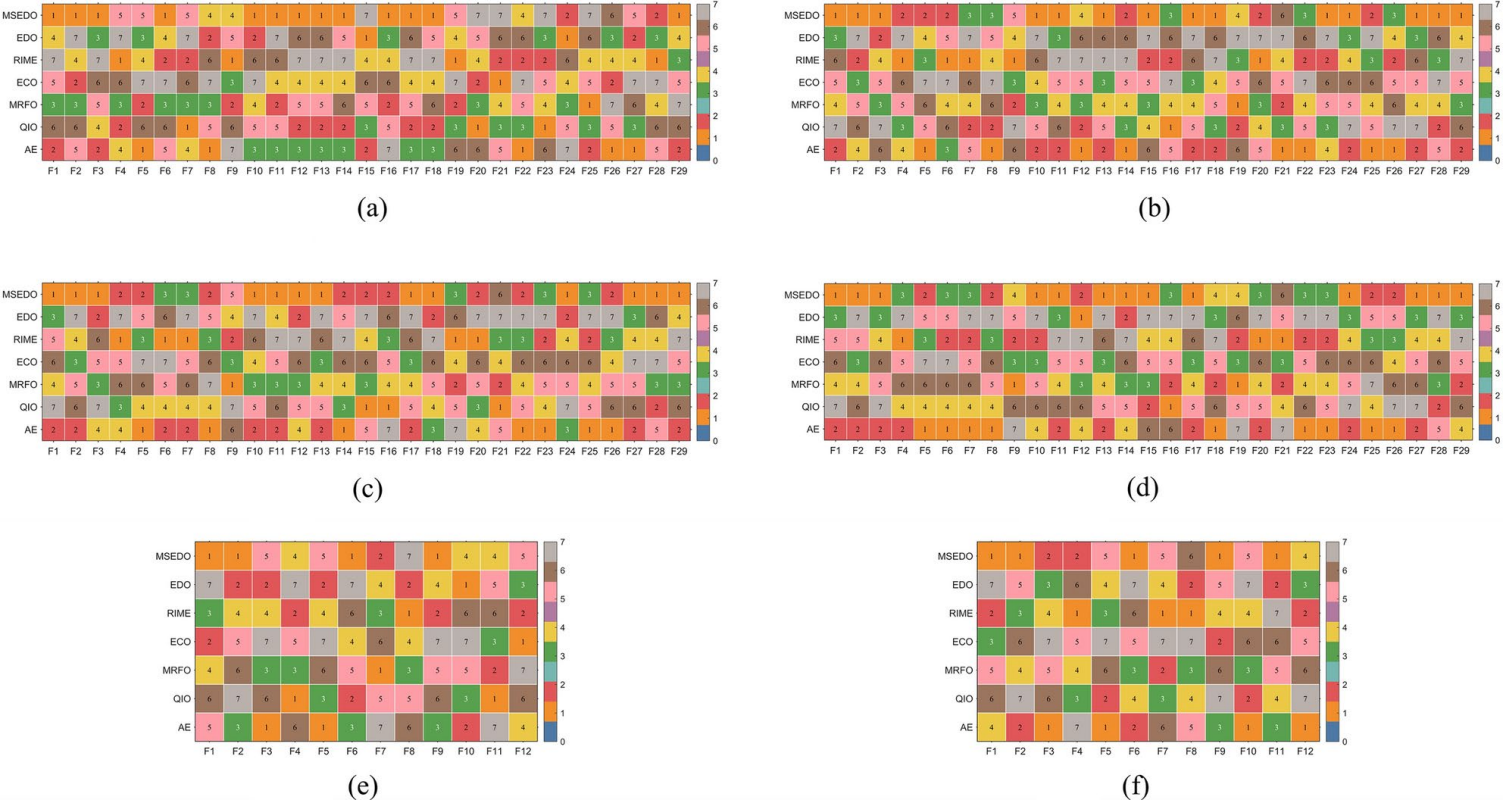
The ranking heat chart of MSEDO and basic competitors.

In this section, the performance of MSEDO is evaluated using the CEC2017 and CEC2022 test sets and compared with five different types of basic meta-heuristic algorithms including: RIME, ECO, MRFO, QIO, and AE. The parameter settings of the compared algorithms can be found in Table 2. Table S3-Table S8 as a Supplementary file provides detailed information on the results obtained by MSEDO and the five basic competitors, containing the best value (Best), the mean (Ave), and the standard deviation (Std). First, a general conclusion is obtained by showing the mean-based ranking of each algorithm on each function. As shown in Fig. 4, MSEDO ranks first the most times among all algorithms. Therefore, we can roughly conclude that MSEDO has a significant superiority compared to the basic algorithms. Next, the experimental results will be analyzed by statistical test methods to avoid the randomness of the results.

#### Analysis of the Wilcoxon rank sum test results

**Fig. 5:**
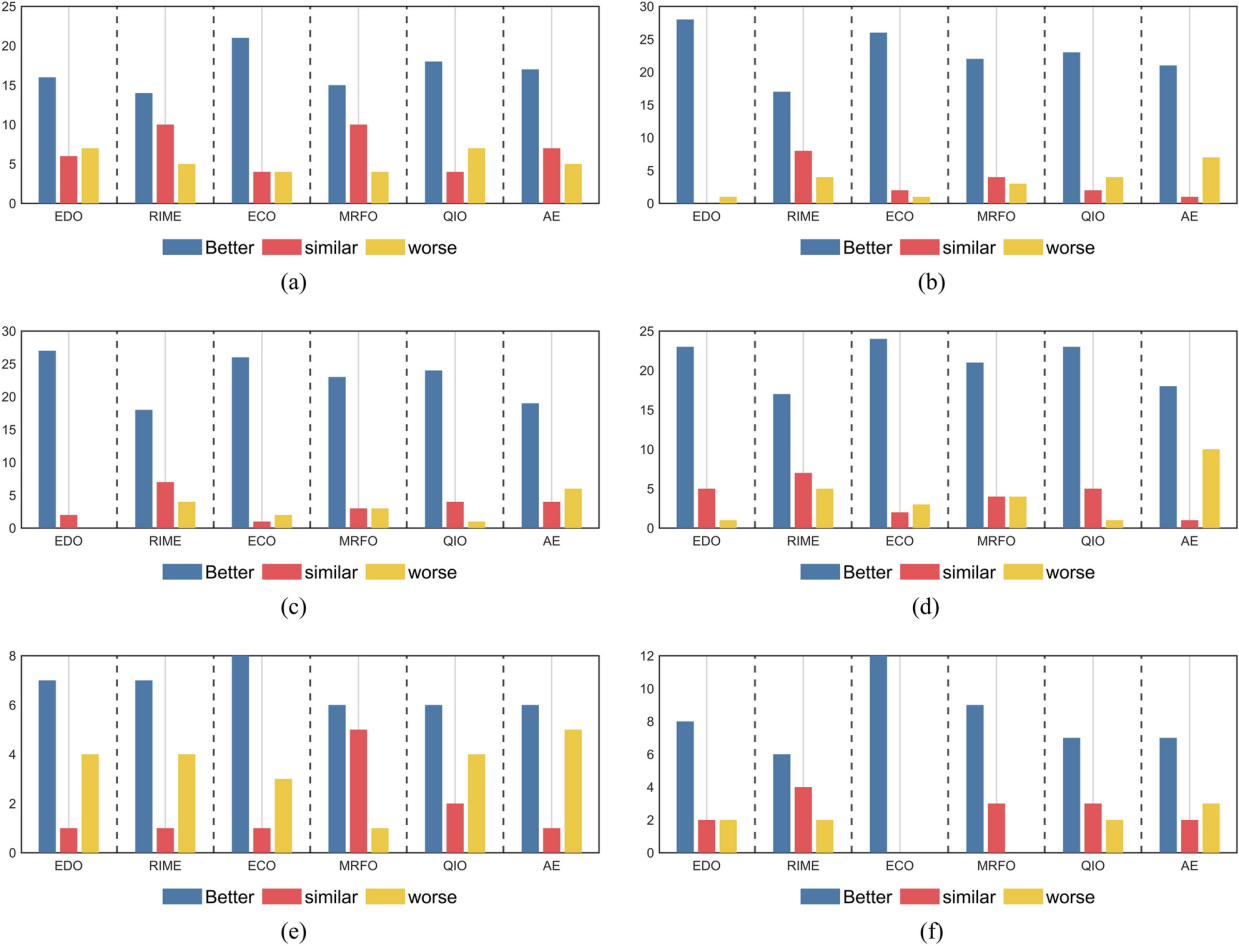
The visualization of Wilcoxon rank sum test results of MSEDO and its basic competitors.

Table 5 summarizes the results of the Wilcoxon rank sum test for the 30 independent outcomes obtained by MSEDO, EDO, RIME, ECO, MRFO, QIO and AE. These results were obtained at the significance level of *p* = 0.05. In Table 5, the symbols “ + / = / − ” imply that MSEDO has significantly better/similar/inferior optimization performance than its competitors. The results of the Wilcoxon rank sum test obtained by MSEDO and the basic comparison algorithms are intuitively presented in Fig. 5. A detailed analysis of the different scenarios is given below.

**Table 5:** Wilcoxon rank sum test results between the MSEDO and basic algorithms.

MSEDO vs. + / = / −	CEC-2017 test suite				CEC-2022 test suite	
	10D	30D	50D	100D	10D	20D
EDO	16/6/7	28/0/1	27/2/0	23/5/1	7/1/4	8/2/2
RIME	14/10/5	17/8/4	18/7/4	17/7/5	7/1/4	6/4/2
ECO	21/4/4	26/2/1	26/1/2	24/2/3	8/1/3	12/0/0
MRFO	15/10/4	22/4/3	23/3/3	21/4/4	6/5/1	9/3/0
QIO	18/4/7	23/2/4	24/4/1	23/5/1	6/2/4	7/3/2
AE	17/7/5	21/1/7	19/4/6	18/1/10	6/1/5	7/2/3

For the CEC2017 10D functions, the performance of IECO-MCO is superior (inferior) to EDO, RIME, ECO, MRFO, QIO and AE on 16(7), 14(5), 21(4), 15(4), 18(7), 17(5) functions. This means MSEDO significantly outperforms the basic competitors.

For the CEC2017 30D functions, the performance of IECO-MCO is superior (inferior) to EDO, RIME, ECO, MRFO, QIO and AE on 28(1), 17(4), 26(1), 22(3), 23(4), 21(7) functions. This means MSEDO significantly outperforms the basic competitors.

For the CEC2017 50D functions, the performance of IECO-MCO is superior (inferior) to EDO, RIME, ECO, MRFO, QIO and AE on 27(0), 18(4), 26(2), 23(3), 24(1), 19(6) functions. This means MSEDO significantly outperforms the basic competitors.

For the CEC2017 100D functions, the performance of IECO-MCO is superior (inferior) to EDO, RIME, ECO, MRFO, QIO and AE on 23(1), 17(5), 24(3), 21(4), 23(1), 18(10) functions. This means MSEDO significantly outperforms the basic competitors.

For the CEC2022 10D functions, the performance of IECO-MCO is superior (inferior) to EDO, RIME, ECO, MRFO, QIO and AE on 7(4), 7(4), 8(3), 6(1), 6(4), 6(5) functions. This means MSEDO significantly outperforms the basic competitors.

For the CEC2022 20D functions, the performance of IECO-MCO is superior (inferior) to EDO, RIME, ECO, MRFO, QIO and AE on 8(2), 6(2), 12(0), 9(0), 7(2), 7(3) functions. This means MSEDO significantly outperforms the basic competitors.

All in all, MSEDO achieves more “ + ” than “ − ” when comparing it to each of its basic competitors, and in most cases the number of “ + ” exceeds the sum of “ − ” and “ = ”. and “ = ”. This means that MSEDO significantly performs better than EDO, RIME, ECO, MRFO, QIO and AE in terms of overall performance. In addition, the advantage of MSEDO becomes more and more obvious with the increase of dimensionality, which means that it is able to deal with the challenges of complex optimization problems with high effectiveness, as confirmed by Fig. 5.

#### Analysis of the Friedman test results

**Fig. 6:**
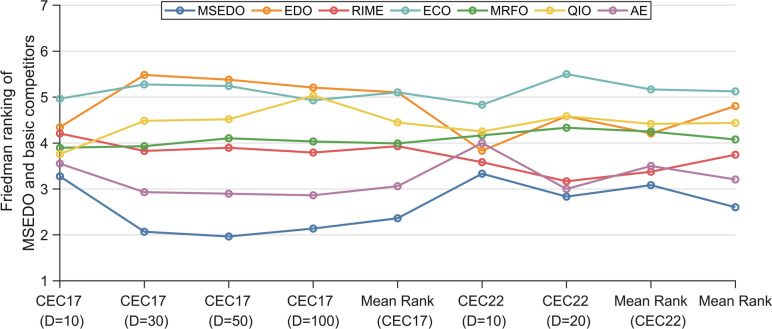
The ranking of MSEDO and basic algorithm derived from the Friedman test.

In this section, the experimental results are statistically analyzed using Friedman test to get the overall performance difference between MSEDO and basic comparison algorithms. Table 6 records the Friedman rankings of MSEDO and the basic competitor for a total of six cases in the two test suites, visualized in Fig. 6. According to Table 6, the *p*-value of the Friedman test for the four cases of CEC2017 is less than 0.05, which indicates that there is a significant difference between the algorithms that participated in the experiments on the CEC2017 test suite. The detailed results of the analysis are as follows.

**Table 6:** The results of MSEDO and basic algorithms derived from the Friedman test.

Algorithm	CEC-2017 test suite					CEC-2022 test suite			
	10D	30D	50D	100D	Mean rank	10D	20D	Mean rank	Total mean rank
MSEDO	3.276	2.069	1.966	2.138	2.362	3.333	2.833	3.083	2.602
EDO	4.345	5.483	5.379	5.207	5.103	3.833	4.583	4.208	4.805
RIME	4.207	3.828	3.897	3.793	3.931	3.583	3.167	3.375	3.746
ECO	4.966	5.276	5.241	4.931	5.103	4.833	5.500	5.167	5.125
MRFO	3.897	3.931	4.103	4.034	3.991	4.167	4.333	4.250	4.078
QIO	3.759	4.483	4.517	5.034	4.448	4.250	4.583	4.417	4.438
AE	3.552	2.931	2.897	2.862	3.060	4.000	3.000	3.500	3.207
Friedman *P*-value	6.82E − 02	3.33E − 10	2.32E − 10	3.02E − 09	N/A	7.20E − 01	1.56E − 02	N/A	N/A

**Fig. 7:**
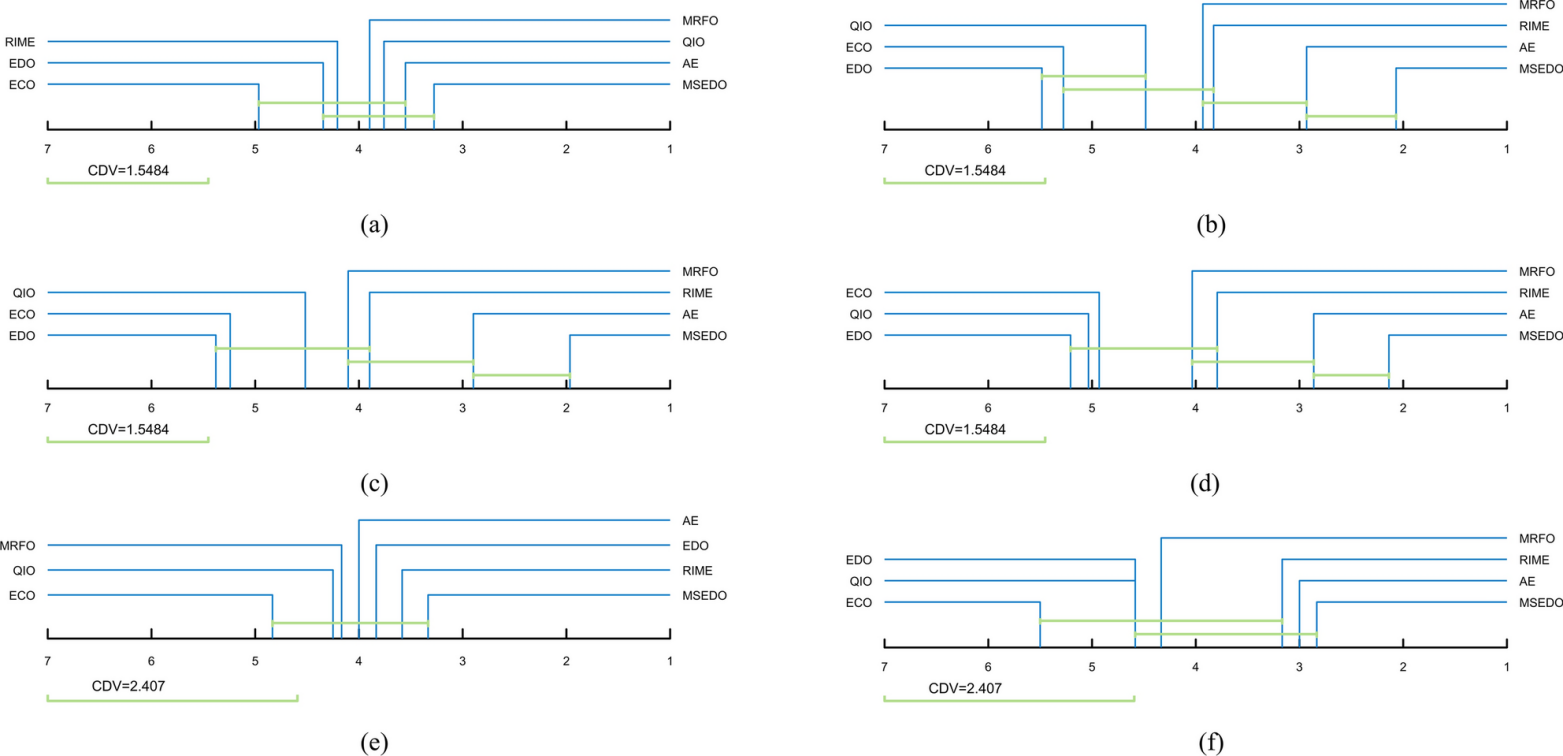
The visualization of Nemenyi test results of MSEDO and its basic competitors.

MSEDO achieved Friedman scores of 3.276, 2.069, 1.966, 2.138, 3.333 and 2.833, all ranking first among the six test groups. Meanwhile, the basic EDO achieved rankings of 4.345, 5.483, 5.379, 5.207, 3.833, and 4.582. According to the “Total mean rank”, MSEDO comes in first place overall, and EDO falls in the second last place, which shows a huge performance difference between MSEDO and EDO. To further analyze the results of the Friedman test, the Nemenyi test was applied as a post hoc test. Figure 7 illustrates the degree of difference between MSEDO and the underlying competitor. According to Fig. 7, MSEDO is not significantly different from the basic EDO on low-dimensional problems (10D/20D) and is significantly better than EDO in other cases. In conclusion, the proposed MSEDO has a clear advantage in comparison with the basic metaheuristic algorithm and is a promising improved algorithm.

### Comparison with improved algorithms

**Fig. 8:**
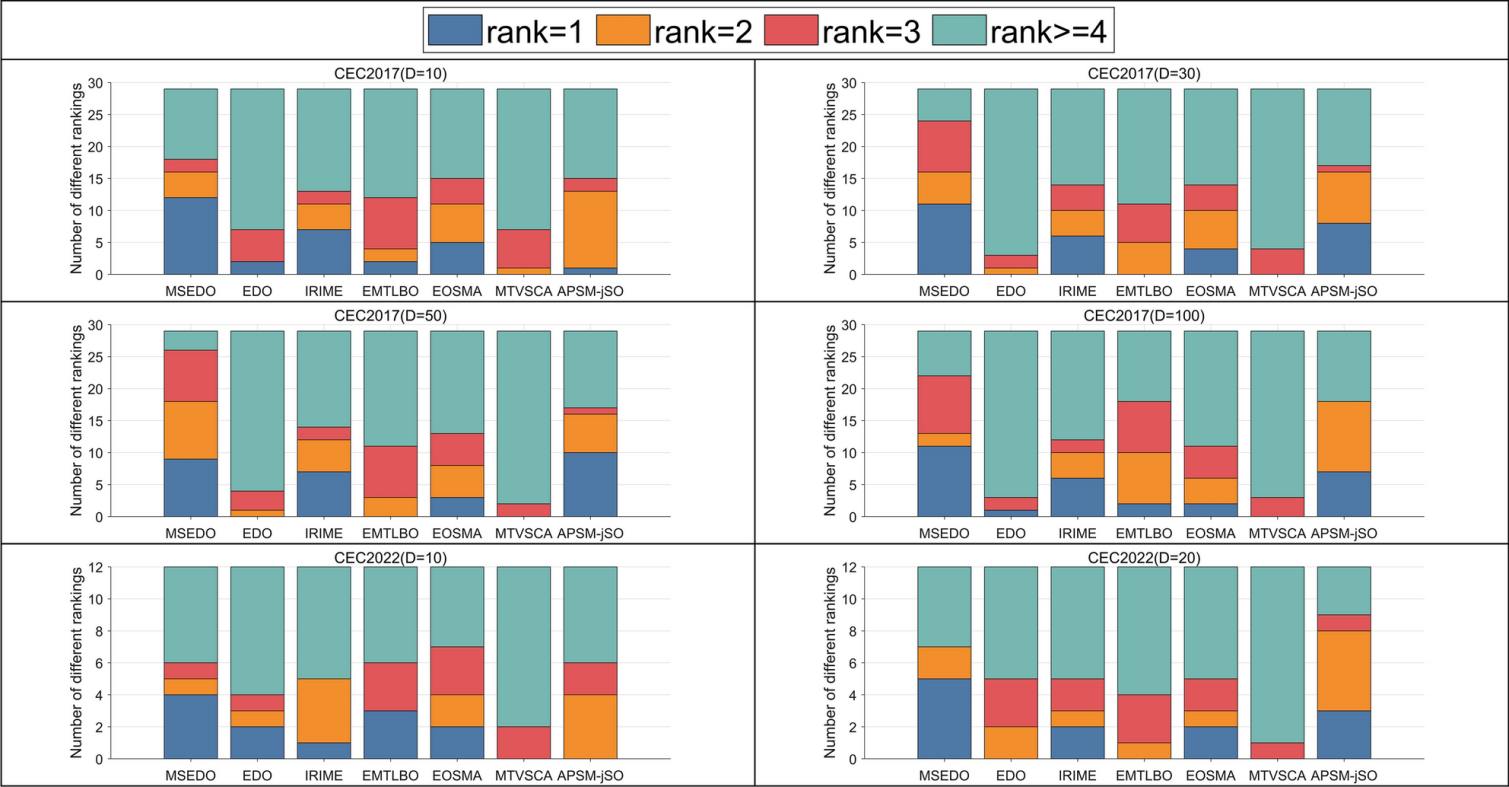
The ranking stack chart of MSEDO and improved competitors.

To further evaluate the performance of the proposed MSEDO, five improved meta-heuristic algorithms from different types are selected as competitors to compare with MSEDO. These improved algorithms include IRIME, EMTLBO, EOSMA, MTVSCA and APSM-jSO. The parameter settings of the compared algorithms can be found in Table 2. Table S9-Table S14 as a Supplementary file provides detailed information on the results obtained by MSEDO and the five improved competitors, containing the best value (Best), the mean (Ave), and the standard deviation (Std). Similarly, the performance of each algorithm is first roughly observed by showing the rankings based on the averages. Figure 8 shows the number of first, second, third, and other rankings achieved by MSEDO and the improved competitors. According to Fig. 8, MSEDO achieved more top three places on the CEC2017 test set. EOSMA and APSM-jSO achieved the most top three places on the 10D and 20D of the CEC2022 test set, respectively. Next, the experimental results will be analyzed by statistical test methods to avoid the randomness of the results.

#### Analysis of the Kruskal Wallis test results

**Fig. 9:**
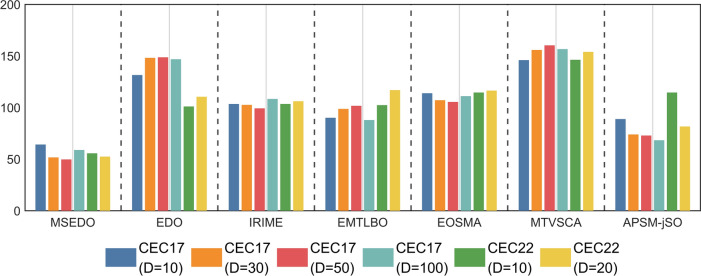
Kruskal Wallis ranking of MSEDO and improved competitors.

The Kruskal–Wallis test was used to analyze the experimental results of MSEDO and its improved competitors on the CEC2017 and CEC2022 test sets. The results of the Kruskal–Wallis test for different dimensions are shown in Fig. 9. According to Fig. 9, the proposed MSEDO obtains the least rankings in all cases and is much less than the rankings of the basic EDO. This shows a great difference between MSEDO and EDO. In addition, we can find that except for EDO, the improved algorithms including MSEDO achieve little Ranking fluctuation, which shows that these improved algorithms have stable performance.

**Fig. 10:**
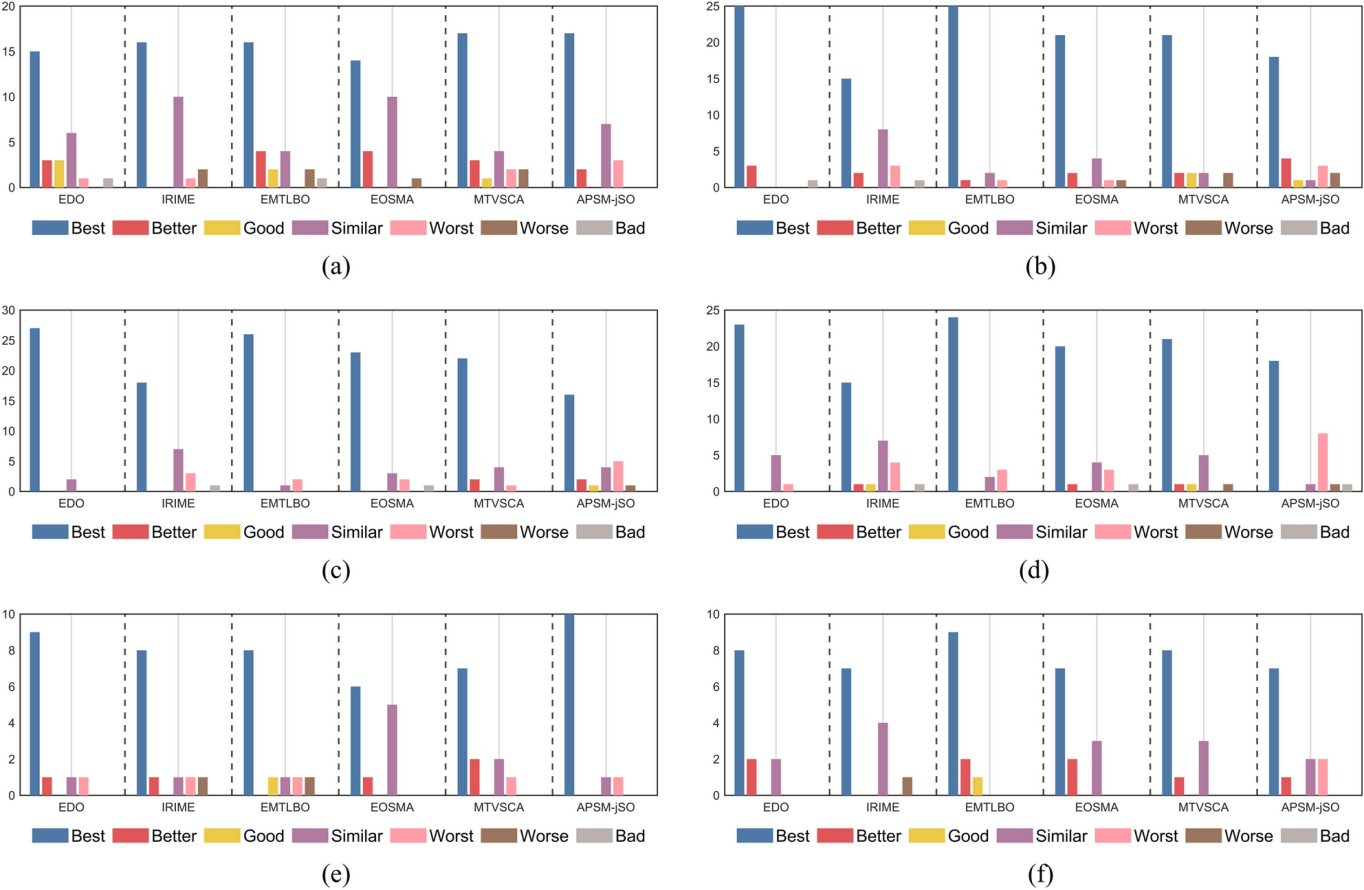
The visualization of Kruskal–Wallis test results of MSEDO and improved competitors.

To further analyze the magnitude of the amount of difference, this paper uses the Cliff Delta test for Effect Size Measure on the experimental results of MSEDO and the improved competitor. The difference is considered particularly significant when the absolute value of the measurement is greater than 0.474, moderately significant when the absolute value of the measurement is greater than 0.33, and generally significant when the absolute value of the measurement is greater than 0.147, the difference is considered generally significant. Figure 10 visualizes the results of the Cliff Delta test. Table 7 records the comparison between MSEDO and the improved competitor. In Table 7, the symbols “ +  +  + / +  + / + ” indicate that MSEDO performs greatly better/better/generally better than the comparison algorithms, respectively. The symbols “ −  − / − / − ” indicate the extent to which MSEDO is inferior to the comparison algorithms.

**Table 7:** Kruskal Wallis test results of MSEDO and improved competitors.

MSEDO vs. + + + / + + / + / = / − − − / − − / −	CEC-2017 test suite				CEC-2022 test suite	
	10D	30D	50D	100D	10D	20D
EDO	15/3/3/6/1/0/1	25/3/0/0/0/0/1	27/0/0/2/0/0/0	23/0/0/5/1/0/0	9/1/0/1/1/0/0	8/2/0/2/0/0/0
IRIME	16/0/0/10/1/2/0	15/2/0/8/3/0/1	18/0/0/7/3/0/1	15/1/1/7/4/0/1	8/1/0/1/1/1/0	7/0/0/4/0/1/0
EMTLBO	16/4/2/4/0/2/1	25/1/0/2/1/0/0	26/0/0/1/2/0/0	24/0/0/2/3/0/0	8/0/1/1/1/1/0	9/2/1/0/0/0/0
EOSMA	14/4/0/10/0/1/0	21/2/0/4/1/1/0	23/0/0/3/2/0/1	20/1/0/4/3/0/1	6/1/0/5/0/0/0	7/2/0/3/0/0/0
MTVSCA	17/3/1/4/2/2/0	21/2/2/2/0/2/0	22/2/0/4/1/0/0	21/1/1/5/0/1/0	7/2/0/2/1/0/0	8/1/0/3/0/0/0
APSM-jSO	17/2/0/7/3/0/0	18/4/1/1/3/2/0	16/2/1/4/5/1/0	18/0/0/1/8/1/1	10/0/0/1/1/0/0	7/1/0/2/2/0/0

According to the results, MSEDO is vastly superior to the comparison algorithms in most of the functions, and the blue bar represented by “Best” stands out, which shows the great advantage of MSEDO over the other competitors. Therefore, we can conclude that the overall performance of MSEDO is better than IRIME, EMTLBO, EOSMA, MTVSCA and APSM-jSO.

#### Analysis of the Friedman test results

**Fig. 11:**
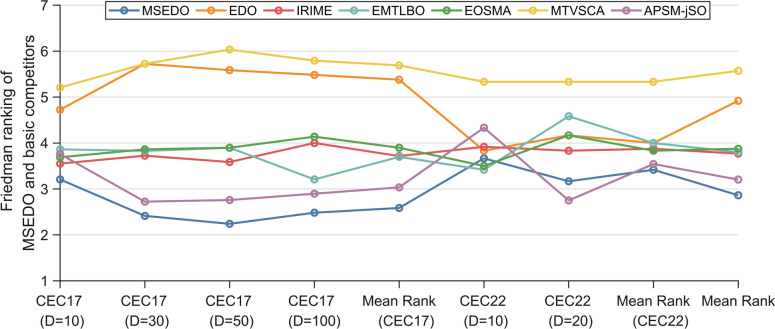
The ranking of MSEDO and improved algorithm derived from the Friedman test.

The scores of MSEDO, IRIME, EMTLBO, EOSMA, MTVSCA and APSM-jSO obtained from the Friedman test are shown in Table 8 and visualized in Fig. 11. The *p*-values of 10D, 30D, 50D, and 100D for the CEC2017 test suite derived from the Friedman test are less than 0.05, which indicates that some significant differences between MSEDO and the five improved algorithms. According to the “Total mean rank” listed in the last column of Table 8, MSEDO occupies the first place, followed by APSM-jSO, IRIME, EMTLBO, EOSMA, EDO and MTVSCA. The detailed analysis is as follows.

**Table 8:** The results of MSEDO and improved algorithms derived from the Friedman test.

Algorithm	CEC-2017 test suite					CEC-2022 test suite			
	10D	30D	50D	100D	Mean rank	10D	20D	Mean rank	Total mean rank
MSEDO	3.207	2.414	2.241	2.483	2.586	3.667	3.167	3.417	2.863
EDO	4.724	5.724	5.586	5.483	5.379	3.833	4.167	4.000	4.920
IRIME	3.552	3.724	3.586	4.000	3.716	3.917	3.833	3.875	3.769
EMTLBO	3.862	3.828	3.897	3.207	3.698	3.417	4.583	4.000	3.799
EOSMA	3.690	3.862	3.897	4.138	3.897	3.500	4.167	3.833	3.875
MTVSCA	5.207	5.724	6.034	5.793	5.690	5.333	5.333	5.333	5.571
APSM-jSO	3.759	2.724	2.759	2.897	3.034	4.333	2.750	3.542	3.204
Friedman *P*-value	5.00E − 03	8.84E − 12	2.16E − 13	2.16E − 13	N/A	3.45E − 01	3.45E − 01	N/A	N/A

For CEC2017 10D, MSEDO ranks in the first place followed by IRIME, EOSMA, APSM-jSO, EMTLBO, EDO and MTVSCA. This shows that MSEDO is outpacing the improved competition at CEC2017 10D.

For CEC2017 30D, MSEDO ranks in the first place followed by APSM-jSO, IRIME, EOSMA, EMTLBO and EDO/MTVSCA. This shows that MSEDO is outpacing the improved competition at CEC2017 30D.

For CEC2017 50D, MSEDO ranks in the first place followed by APSM-jSO, IRIME, EOSMA/EMTLBO, EDO and MTVSCA. This shows that MSEDO is outpacing the improved competition at CEC2017 50D.

For CEC2017 100D, MSEDO ranks in the first place followed by APSM-jSO, EMTLBO, IRIME, EOSMA, EDO and MTVSCA. This shows that MSEDO is outpacing the improved competition at CEC2017 100D.

For CEC2022 10D, MSEDO ranks in the third place following EMTLBO and EOSMA, followed by EDO, IRIME, APSM-jSO, and MTVSCA. This shows that MSEDO is outpacing EDO, IRIME, APSM-jSO, and MTVSCA, but is inferior to EMTLBO and EOSMA at CEC2022 10D.

For CEC2022 20D, MSEDO ranks in the third place following APSM-jSO, followed by IRIME, EOSMA/EDO, EMTLBO and MTVSCA. This shows that MSEDO is outpacing IRIME, EOSMA, EDO, EMTLBO and MTVSCA, but is inferior to APSM-jSO at CEC2022 20D.

Based on the above analysis, MSEDO outperforms all the improved comparison algorithms on 10D, 30D,50D and 100D for CEC2017 test suite, but is inferior to EOSMA and EMTLBO on 10D for CEC2022 test suite, and inferior to APSM-jSO on 20D for CEC2022 test suite. However, this ranking gap is small and MSEDO has the best mean ranking in the CEC2022 test suite, so we can conclude that the overall performance of MSEDO is the best of all the compared algorithms.

### Comparison using constrained engineering optimization

After the experiments in the previous subsections, we confirmed the superior performance of MSEDO on complex functions. In this subsection, we evaluate the potential of MSEDO to solve real-world optimization problems through 10 engineering constrained optimization problems. Three each from the basic and improved algorithms are selected along with EDO as comparison algorithms for this test. Table 9 shows the details of these engineering constrained optimization problems. For the constrained optimization problems, a penalty function approach is adopted to transform them into unconstrained optimization problems. The results of 30 independent runs are shown in Table S15 in the Supplementary file.

**Table 9:** Ten constrained engineering optimization problems.

Problem	Name	D
CE01	Tension/compression spring design problem	3
CE02	Pressure vessel design problem	4
CE03	Three-bar truss design problem	2
CE04	Welded beam design problem	4
CE05	Speed reducer design problem	7
CE06	Gear train design problem	4
CE07	Rolling element bearing design	10
CE08	Cantilever beam design problem	5
CE09	Multiple disk clutch brake design problem	5
CE10	Step-cone pulley problem	5

**Fig. 12:**
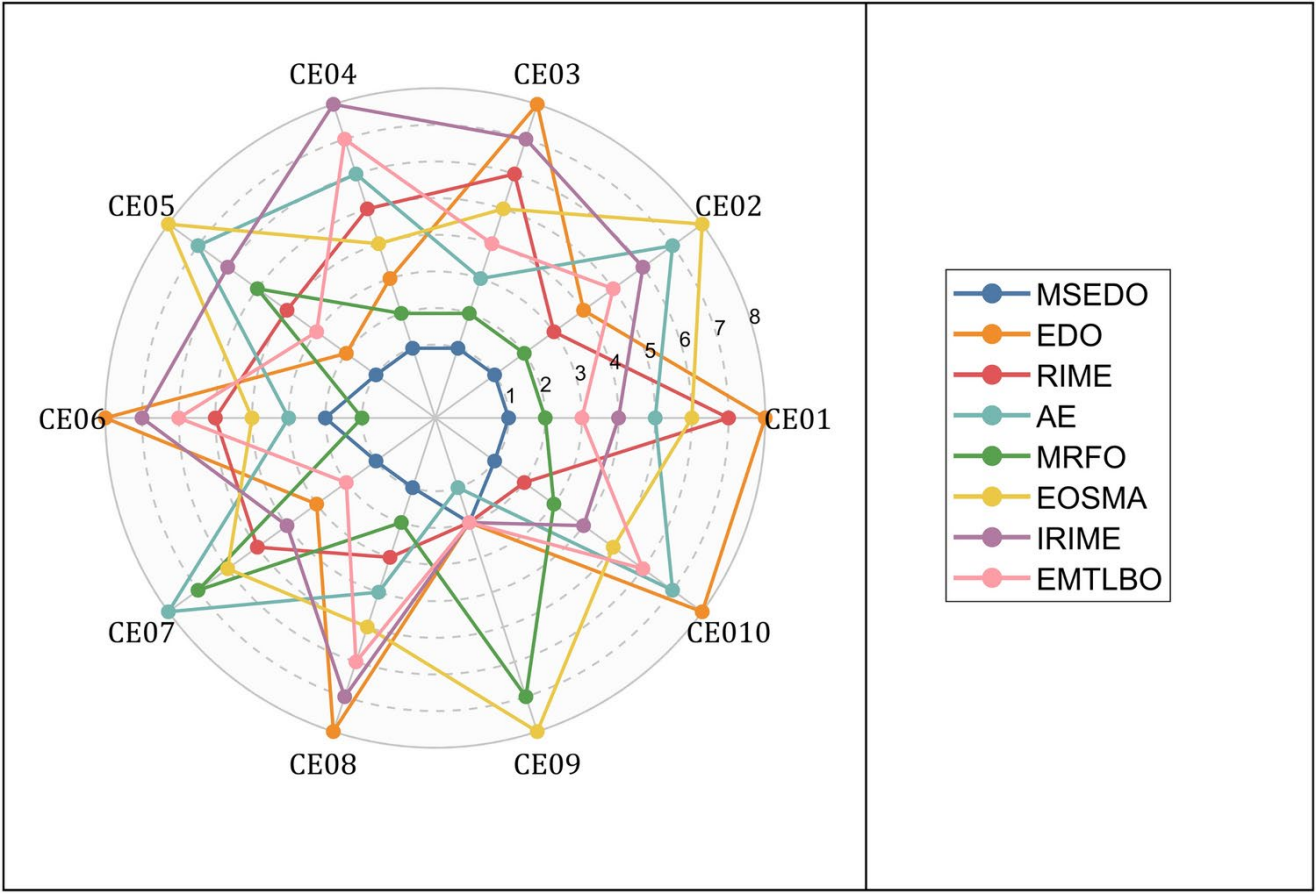
Rankings of MSEDO and its competitors on constrained optimization problems.

Figure 12 visualizes the performance of MSEDO and the comparison algorithms in solving engineering constraint problems. According to Fig. 12, MSEDO provides the best solution on 8 constrained problems and the second-best approach on 2 constrained problems. This indicates that the proposed MSEDO has a great potential to solve real-world problems.

### Strengths and limitations of MSEDO

The MSEDO algorithm performs better on high-dimensional problems than on low-dimensional ones. The superior performance on high-dimensional problems is due to the introduction of the diversity-based population restart strategy. This strategy reconstructs the algorithm structure, spends more computational cost on development and exploration, and ensures diversity of understanding through dimension swapping. The performance improvement of MSEDO on low-dimensional problems relies on the bootstrapping effect of the leader-based covariance learning strategy. This strategy promotes high-quality population evolution by absorbing valid information from dominant populations. In addition, the fitness and distance-based leader selection strategy improves the convergence efficiency of the MSEDO algorithm, and the roulette approach well balances the global search and local exploitation.

However, MSEDO also has some shortcomings. From the results in Appendix, the proposed MSEDO performs better on most problems. However, when facing some complex functions or mixed numbers, MSEDO is not capable of providing the best solution. When dealing with low-dimensional problems, MSEDO does not create a gap with EDO, which indicates that the improvement strategy proposed in this paper is more suitable for complex optimization problems.

## Conclusions

In this paper, we propose a multi-strategy enterprise development optimizer (MSEDO) that aims to comprehensively facilitate the exploitation and exploration performance of EDO. By integrating leader-based covariance learning strategy, diversity-based population restart strategy and fitness and distance-based leader selection strategy, MSEDO is equipped to solve complex optimization problems. Experiments on the CEC2017 and CEC2022 test sets confirm the overall superiority of the proposed technique over basic EDO and other basic or improved meta-heuristic algorithms. In addition, the ability of MSEDO to solve real-world problems is examined by solving engineering constraints. Future research is planned to proceed in the following directions.

1. Direction of algorithm application: apply MSEDO to specific research areas, such as enterprise resource allocation optimization, production scheduling optimization, and asset management optimization.
2. Direction of algorithm development: develop multi-objective and binary versions.
3. Direction of algorithm improvement: explore the combination of meta-heuristic algorithms and machine learning methods such as reinforcement learning and deep learning.

## Electronic supplementary material

Below is the link to the electronic supplementary material.

Supplementary Information.

## Data Availability

The data that support the findings of this study are available from the corresponding author.

## Appendix

See Tables 10, 11, 12, 13, 14, 15, 16, 17, 18, 19, 20, 21, 22, 23, 24.

**Table 10:** CEC 2017 test functions.

Type	ID	CEC 2017 Function name	Search range	fmin
Unimodal	F1	Shifted and Rotated Bent Cigar Function	[− 100,100]	100
	F2	Shifted and Rotated Zakharov Function	[− 100,100]	300
Multimodal	F3	Shifted and Rotated Rosenbrock’s Function	[− 100,100]	400
	F4	Shifted and Rotated Rastrigin’s Function	[− 100,100]	500
	F5	Shifted and Rotated Expanded Scaffer’s F6 Function	[− 100,100]	600
	F6	Shifted and Rotated Lunacek Bi_Rastrigin Function	[− 100,100]	700
	F7	Shifted and Rotated Non-Continuous Rastrigin’s Function	[− 100,100]	800
	F8	Shifted and Rotated Levy Function	[− 100,100]	900
	F9	Shifted and Rotated Schwefel’s Function	[− 100,100]	1000
Hybrid	F10	Hybrid Function 1 (N = 3)	[− 100,100]	1100
	F11	Hybrid Function 2 (N = 3)	[− 100,100]	1200
	F12	Hybrid Function 3 (N = 3)	[− 100,100]	1300
	F13	Hybrid Function 4 (N = 4)	[− 100,100]	1400
	F14	Hybrid Function 5 (N = 4)	[− 100,100]	1500
	F15	Hybrid Function 6 (N = 4)	[− 100,100]	1600
	F16	Hybrid Function 6 (N = 5)	[− 100,100]	1700
	F17	Hybrid Function 6 (N = 5)	[− 100,100]	1800
	F18	Hybrid Function 6 (N = 5)	[− 100,100]	1900
	F19	Hybrid Function 6 (N = 6)	[− 100,100]	2000
Composition	F20	Composition Function 1 (N = 3)	[− 100,100]	2100
	F21	Composition Function 2 (N = 3)	[− 100,100]	2200
	F22	Composition Function 3 (N = 4)	[− 100,100]	2300
	F23	Composition Function 4 (N = 4)	[− 100,100]	2400
	F24	Composition Function 5 (N = 5)	[− 100,100]	2500
	F25	Composition Function 6 (N = 5)	[− 100,100]	2600
	F26	Composition Function 7 (N = 6)	[− 100,100]	2700
	F27	Composition Function 8 (N = 6)	[− 100,100]	2800
	F28	Composition Function 9 (N = 3)	[− 100,100]	2900
	F29	Composition Function 10 (N = 3)	[− 100,100]	3000

**Table 11:** CEC 2022 test functions.

Type	ID	CEC 2022 Function name	Search range	f_min_
Unimodal functions	F1	Shifted and full Rotated Zakharov Function	[− 100,100]	300
Multimodal functions	F2	Shifted and full Rotated Rosenbrock’s Function	[− 100,100]	400
	F3	Shifted and full Rotated Expanded Schaffer’s f6 Function	[− 100,100]	600
	F4	Shifted and full Rotated Non-Continuous Rastrigin’s Function	[− 100,100]	800
	F5	Shifted and full Rotated Levy Function	[− 100,100]	900
Hybrid functions	F6	Hybrid Function 1 (N = 3)	[− 100,100]	1800
	F7	Hybrid Function 2 (N = 6	[− 100,100]	2000
	F8	Hybrid Function 3 (N = 5)	[− 100,100]	2200
Composition functions	F9	Composition Function 1 (N = 5)	[− 100,100]	2300
	F10	Composition Function 2 (N = 4)	[− 100,100]	2400
	F11	Composition Function 3 (N = 5)	[− 100,100]	2600
	F12	Composition Function 4 (N = 6)	[− 100,100]	2700

**Table 12:** The statistics results of MSEDO and basic algorithms solving CEC2017 (D = 10).

No	Index	MSEDO	EDO	RIME	ECO	MRFO	QIO	AE
F1	Best	1.0001E + 02	1.0014E + 02	7.1759E + 03	1.0146E + 02	1.0152E + 02	2.1725E + 03	1.0175E + 02
	Mean	1.0025E + 02	3.5109E + 03	3.5879E + 04	3.7647E + 03	1.2944E + 03	2.0785E + 04	3.5687E + 02
	Std	1.2520E + 00	2.7405E + 03	2.1751E + 04	3.8188E + 03	1.3843E + 03	2.1482E + 04	3.5630E + 02
	Rank	1	4	7	5	3	6	2
F2	Best	3.0000E + 02	1.0202E + 03	3.0027E + 02	3.0000E + 02	3.0002E + 02	3.3928E + 02	3.0229E + 02
	Mean	3.0000E + 02	3.1180E + 03	3.0140E + 02	3.0014E + 02	3.0028E + 02	5.5043E + 02	3.1035E + 02
	Std	9.9581E − 14	1.5322E + 03	7.9329E − 01	1.9823E − 01	3.7541E − 01	1.6905E + 02	7.1284E + 00
	Rank	1	7	4	2	3	6	5
F3	Best	4.0000E + 02	4.0243E + 02	4.0011E + 02	4.0003E + 02	4.0001E + 02	4.0033E + 02	4.0422E + 02
	Mean	4.0000E + 02	4.0477E + 02	4.0786E + 02	4.0680E + 02	4.0594E + 02	4.0488E + 02	4.0467E + 02
	Std	8.6802E − 10	7.3177E − 01	1.2133E + 01	1.2953E + 01	1.2775E + 01	2.1774E + 00	2.1146E − 01
	Rank	1	3	7	6	5	4	2
F4	Best	5.0398E + 02	5.1360E + 02	5.0401E + 02	5.0913E + 02	5.0398E + 02	5.0225E + 02	5.0092E + 02
	Mean	5.2172E + 02	5.2351E + 02	5.1355E + 02	5.2323E + 02	5.2008E + 02	5.1777E + 02	5.2110E + 02
	Std	2.3103E + 01	4.9301E + 00	6.2207E + 00	1.0744E + 01	9.4699E + 00	6.8669E + 00	9.1780E + 00
	Rank	5	7	1	6	3	2	4
F5	Best	6.0000E + 02	6.0000E + 02	6.0007E + 02	6.0017E + 02	6.0000E + 02	6.0023E + 02	6.0000E + 02
	Mean	6.0026E + 02	6.0010E + 02	6.0022E + 02	6.0744E + 02	6.0005E + 02	6.0060E + 02	6.0000E + 02
	Std	1.0890E + 00	3.0012E − 01	9.0413E − 02	5.9070E + 00	2.1475E − 01	3.4784E − 01	9.8908E − 04
	Rank	5	3	4	7	2	6	1
F6	Best	7.0533E + 02	7.2212E + 02	7.0898E + 02	7.2037E + 02	7.1462E + 02	7.2096E + 02	7.1953E + 02
	Mean	7.2163E + 02	7.3284E + 02	7.2278E + 02	7.4163E + 02	7.3282E + 02	7.4014E + 02	7.3530E + 02
	Std	9.1725E + 00	6.1432E + 00	6.5389E + 00	1.6724E + 01	8.2380E + 00	6.8364E + 00	5.7373E + 00
	Rank	1	4	2	7	3	6	5
F7	Best	8.0398E + 02	8.1182E + 02	8.0799E + 02	8.0598E + 02	8.0597E + 02	8.0298E + 02	8.0074E + 02
	Mean	8.2106E + 02	8.2262E + 02	8.1379E + 02	8.2220E + 02	8.1724E + 02	8.1272E + 02	8.1836E + 02
	Std	1.1172E + 01	4.6598E + 00	4.1310E + 00	7.1955E + 00	7.0681E + 00	5.2485E + 00	9.7398E + 00
	Rank	5	7	2	6	3	1	4
F8	Best	9.0000E + 02	9.0000E + 02	9.0001E + 02	9.0000E + 02	9.0000E + 02	9.0002E + 02	9.0000E + 02
	Mean	9.0009E + 02	9.0002E + 02	9.0025E + 02	9.2885E + 02	9.0006E + 02	9.0019E + 02	9.0000E + 02
	Std	1.9151E − 01	8.4851E − 02	2.9480E − 01	3.8848E + 01	1.5084E − 01	1.8035E − 01	3.5628E − 06
	Rank	4	2	6	7	3	5	1
F9	Best	1.1231E + 03	1.9125E + 03	1.1303E + 03	1.2546E + 03	1.2404E + 03	1.4893E + 03	1.9099E + 03
	Mean	1.7766E + 03	2.0972E + 03	1.4579E + 03	1.7255E + 03	1.6927E + 03	2.3299E + 03	2.3707E + 03
	Std	3.3407E + 02	1.0050E + 02	2.5502E + 02	2.9233E + 02	2.6921E + 02	3.0748E + 02	1.8050E + 02
	Rank	4	5	1	3	2	6	7
F10	Best	1.1000E + 03	1.1017E + 03	1.1029E + 03	1.1078E + 03	1.1014E + 03	1.1052E + 03	1.1042E + 03
	Mean	1.1058E + 03	1.1064E + 03	1.1123E + 03	1.1362E + 03	1.1081E + 03	1.1102E + 03	1.1074E + 03
	Std	4.9329E + 00	2.9064E + 00	6.0384E + 00	4.5102E + 01	4.5870E + 00	3.7743E + 00	2.0807E + 00
	Rank	1	2	6	7	4	5	3
F11	Best	1.2000E + 03	2.1599E + 04	1.0347E + 04	2.2292E + 03	2.8316E + 03	7.4045E + 03	6.4496E + 03
	Mean	1.2664E + 03	3.0129E + 05	1.8735E + 05	2.1422E + 04	1.4341E + 04	3.9582E + 04	1.6894E + 04
	Std	9.3930E + 01	3.6239E + 05	3.5720E + 05	1.8320E + 04	1.0465E + 04	3.5059E + 04	4.7285E + 03
	Rank	1	7	6	4	2	5	3
F12	Best	1.3003E + 03	1.3689E + 03	1.3418E + 03	1.5918E + 03	1.3540E + 03	1.3161E + 03	1.3792E + 03
	Mean	1.3039E + 03	5.0469E + 03	8.7790E + 03	2.1893E + 03	2.8525E + 03	1.3353E + 03	1.5788E + 03
	Std	3.3659E + 00	5.6742E + 03	7.6455E + 03	5.9496E + 02	1.7069E + 03	1.2175E + 01	9.1140E + 01
	Rank	1	6	7	4	5	2	3
F13	Best	1.4000E + 03	1.4458E + 03	1.4076E + 03	1.4271E + 03	1.4410E + 03	1.4117E + 03	1.4288E + 03
	Mean	1.4031E + 03	1.5549E + 03	2.1627E + 03	1.4798E + 03	1.4927E + 03	1.4227E + 03	1.4411E + 03
	Std	5.7833E + 00	2.2567E + 02	1.6563E + 03	3.0417E + 01	2.8265E + 01	5.0204E + 00	5.4244E + 00
	Rank	1	6	7	4	5	2	3
F14	Best	1.5000E + 03	1.5358E + 03	1.5076E + 03	1.5191E + 03	1.5494E + 03	1.5030E + 03	1.5221E + 03
	Mean	1.5014E + 03	1.7364E + 03	2.8904E + 03	1.6340E + 03	1.8289E + 03	1.5081E + 03	1.5414E + 03
	Std	5.7709E + 00	2.3632E + 02	2.5843E + 03	7.4486E + 01	4.0742E + 02	2.7037E + 00	1.0479E + 01
	Rank	1	5	7	4	6	2	3
F15	Best	1.6000E + 03	1.6036E + 03	1.6017E + 03	1.6023E + 03	1.6012E + 03	1.6022E + 03	1.6078E + 03
	Mean	1.7623E + 03	1.6352E + 03	1.6646E + 03	1.6849E + 03	1.6837E + 03	1.6498E + 03	1.6423E + 03
	Std	1.0048E + 02	3.4732E + 01	8.5690E + 01	6.1668E + 01	1.0147E + 02	6.1285E + 01	1.9373E + 01
	Rank	7	1	4	6	5	3	2
F16	Best	1.7024E + 03	1.7171E + 03	1.7034E + 03	1.7221E + 03	1.7021E + 03	1.7282E + 03	1.7470E + 03
	Mean	1.7208E + 03	1.7369E + 03	1.7448E + 03	1.7545E + 03	1.7332E + 03	1.7505E + 03	1.7693E + 03
	Std	2.7687E + 01	1.0117E + 01	4.2459E + 01	1.5166E + 01	1.7072E + 01	1.4698E + 01	1.0719E + 01
	Rank	1	3	4	6	2	5	7
F17	Best	1.8000E + 03	4.1404E + 03	1.8504E + 03	1.8942E + 03	2.0448E + 03	1.8233E + 03	1.9154E + 03
	Mean	1.8032E + 03	8.6641E + 03	1.4029E + 04	4.4660E + 03	6.7194E + 03	1.8335E + 03	2.1203E + 03
	Std	8.4084E + 00	4.6488E + 03	9.3918E + 03	4.8197E + 03	4.5662E + 03	9.6080E + 00	1.2341E + 02
	Rank	1	6	7	4	5	2	3
F18	Best	1.9000E + 03	1.9208E + 03	1.9063E + 03	1.9099E + 03	1.9437E + 03	1.9038E + 03	1.9072E + 03
	Mean	1.9015E + 03	1.9758E + 03	2.5450E + 03	1.9552E + 03	2.1147E + 03	1.9056E + 03	1.9166E + 03
	Std	6.7051E + 00	6.8381E + 01	1.1042E + 03	3.8374E + 01	2.4171E + 02	9.3842E − 01	5.3919E + 00
	Rank	1	5	7	4	6	2	3
F19	Best	2.0003E + 03	2.0243E + 03	2.0004E + 03	2.0231E + 03	2.0013E + 03	2.0188E + 03	2.0342E + 03
	Mean	2.0478E + 03	2.0436E + 03	2.0170E + 03	2.0676E + 03	2.0214E + 03	2.0422E + 03	2.0535E + 03
	Std	6.2270E + 01	9.9469E + 00	1.0933E + 01	3.1634E + 01	1.2639E + 01	1.4614E + 01	1.0846E + 01
	Rank	5	4	1	7	2	3	6
F20	Best	2.1000E + 03	2.2026E + 03	2.1001E + 03	2.2002E + 03	2.2000E + 03	2.2004E + 03	2.2015E + 03
	Mean	2.2855E + 03	2.2457E + 03	2.2452E + 03	2.2243E + 03	2.2294E + 03	2.2106E + 03	2.2759E + 03
	Std	5.9326E + 01	5.5662E + 01	6.4433E + 01	4.8349E + 01	5.1012E + 01	3.0392E + 01	5.7710E + 01
	Rank	7	5	4	2	3	1	6
F21	Best	2.3003E + 03	2.2197E + 03	2.2002E + 03	2.2000E + 03	2.2328E + 03	2.2003E + 03	2.3000E + 03
	Mean	2.3919E + 03	2.3221E + 03	2.2923E + 03	2.2810E + 03	2.2982E + 03	2.2951E + 03	2.3000E + 03
	Std	2.9812E + 02	1.4412E + 02	2.9375E + 01	3.5131E + 01	1.6446E + 01	2.9251E + 01	1.6330E − 02
	Rank	7	6	2	1	4	3	5
F22	Best	2.6055E + 03	2.6104E + 03	2.6057E + 03	2.6088E + 03	2.6060E + 03	2.6030E + 03	2.6000E + 03
	Mean	2.6162E + 03	2.6208E + 03	2.6146E + 03	2.6228E + 03	2.6183E + 03	2.6152E + 03	2.6139E + 03
	Std	6.3927E + 00	6.0258E + 00	5.2787E + 00	1.0209E + 01	9.9894E + 00	9.1539E + 00	1.0328E + 01
	Rank	4	6	2	7	5	3	1
F23	Best	2.5000E + 03	2.5092E + 03	2.5002E + 03	2.5002E + 03	2.5000E + 03	2.5007E + 03	2.5653E + 03
	Mean	2.7365E + 03	2.7101E + 03	2.6865E + 03	2.7267E + 03	2.7267E + 03	2.6656E + 03	2.7346E + 03
	Std	4.5287E + 01	8.7104E + 01	1.0789E + 02	7.7404E + 01	6.4044E + 01	1.0963E + 02	3.3652E + 01
	Rank	7	3	2	5	4	1	6
F24	Best	2.6000E + 03	2.6367E + 03	2.8978E + 03	2.6005E + 03	2.8977E + 03	2.8978E + 03	2.8983E + 03
	Mean	2.9062E + 03	2.9050E + 03	2.9353E + 03	2.9175E + 03	2.9138E + 03	2.9177E + 03	2.9374E + 03
	Std	6.2537E + 01	5.5194E + 01	2.0500E + 01	6.4410E + 01	2.2419E + 01	2.2684E + 01	1.7725E + 01
	Rank	2	1	6	4	3	5	7
F25	Best	2.8000E + 03	2.6019E + 03	2.8058E + 03	2.8009E + 03	2.8000E + 03	2.8026E + 03	2.9000E + 03
	Mean	3.3659E + 03	3.0345E + 03	2.9064E + 03	2.9565E + 03	2.8984E + 03	2.9020E + 03	2.9000E + 03
	Std	5.4672E + 02	3.8362E + 02	4.0183E + 01	7.0871E + 01	4.5126E + 01	2.1759E + 01	6.0437E − 03
	Rank	7	6	4	5	1	3	2
F26	Best	3.0889E + 03	3.0891E + 03	3.0893E + 03	3.0893E + 03	3.0896E + 03	3.0905E + 03	3.0895E + 03
	Mean	3.1003E + 03	3.0943E + 03	3.0957E + 03	3.0939E + 03	3.1036E + 03	3.0983E + 03	3.0913E + 03
	Std	1.7097E + 01	2.8280E + 00	9.4598E + 00	3.4982E + 00	1.8246E + 01	3.0988E + 00	2.2581E + 00
	Rank	6	3	4	2	7	5	1
F27	Best	3.1000E + 03	3.1000E + 03	3.1004E + 03	3.1001E + 03	3.1000E + 03	3.1007E + 03	3.1000E + 03
	Mean	3.2052E + 03	3.1300E + 03	3.1978E + 03	3.2890E + 03	3.2235E + 03	3.1558E + 03	3.1070E + 03
	Std	1.3135E + 02	3.3936E + 01	9.3661E + 01	1.4280E + 02	1.4344E + 02	8.2713E + 01	1.6675E + 01
	Rank	5	2	4	7	6	3	1
F28	Best	3.1293E + 03	3.1543E + 03	3.1402E + 03	3.1445E + 03	3.1473E + 03	3.1584E + 03	3.1702E + 03
	Mean	3.1842E + 03	3.1863E + 03	3.1778E + 03	3.2082E + 03	3.1916E + 03	3.1996E + 03	3.1986E + 03
	Std	6.6041E + 01	1.6812E + 01	2.8551E + 01	4.3985E + 01	2.6607E + 01	2.0587E + 01	1.4668E + 01
	Rank	2	3	1	7	4	6	5
F29	Best	3.3945E + 03	1.3839E + 04	3.7232E + 03	3.5547E + 03	4.3751E + 03	5.0412E + 03	5.8828E + 03
	Mean	3.0644E + 04	5.7709E + 04	5.6301E + 04	1.8563E + 05	3.8093E + 05	2.0385E + 05	5.5625E + 04
	Std	1.4920E + 05	4.5589E + 04	1.4916E + 05	5.0809E + 05	6.1755E + 05	4.0759E + 05	7.1338E + 04
	Rank	1	4	3	5	7	6	2

**Table 13:** The statistics results of MSEDO and basic algorithms solving CEC2017 (D = 30).

No	Index	MSEDO	EDO	RIME	ECO	MRFO	QIO	AE
F1	Best	1.0003E + 02	3.0527E + 02	4.9665E + 05	8.1707E + 04	9.0817E + 02	4.1892E + 06	5.7916E + 02
	Mean	1.0148E + 02	3.0404E + 03	1.6954E + 06	4.4234E + 05	6.3096E + 03	8.4807E + 06	1.9964E + 03
	Std	4.1493E − 01	3.6727E + 03	9.8409E + 05	3.3145E + 05	4.6497E + 03	3.0371E + 06	6.5929E + 02
	Rank	1	3	6	5	4	7	2
F2	Best	3.0000E + 02	6.0706E + 04	2.0915E + 03	2.2586E + 03	4.6619E + 03	3.3664E + 04	3.5574E + 03
	Mean	3.0000E + 02	8.5772E + 04	4.7443E + 03	8.1312E + 03	8.5537E + 03	5.2848E + 04	8.1368E + 03
	Std	7.2411E − 07	1.3340E + 04	1.8712E + 03	4.0344E + 03	2.7772E + 03	7.6591E + 03	2.6268E + 03
	Rank	1	7	2	3	5	6	4
F3	Best	4.0000E + 02	4.0424E + 02	4.7408E + 02	4.6482E + 02	4.0661E + 02	4.7077E + 02	4.8630E + 02
	Mean	4.2235E + 02	4.9474E + 02	5.0510E + 02	5.0628E + 02	5.0342E + 02	5.3218E + 02	5.1113E + 02
	Std	2.9431E + 01	3.1250E + 01	2.0318E + 01	1.7569E + 01	2.7837E + 01	2.5368E + 01	1.0680E + 01
	Rank	1	2	4	5	3	7	6
F4	Best	5.4079E + 02	6.1971E + 02	5.4528E + 02	5.7371E + 02	5.7761E + 02	5.3945E + 02	5.1309E + 02
	Mean	5.8872E + 02	6.7574E + 02	5.7123E + 02	6.6124E + 02	6.3920E + 02	6.0573E + 02	6.2968E + 02
	Std	3.2737E + 01	2.0990E + 01	1.3785E + 01	4.5002E + 01	3.9586E + 01	3.2983E + 01	5.9231E + 01
	Rank	2	7	1	6	5	3	4
F5	Best	6.0000E + 02	6.0068E + 02	6.0070E + 02	6.2182E + 02	6.0041E + 02	6.0287E + 02	6.0002E + 02
	Mean	6.0197E + 02	6.0480E + 02	6.0242E + 02	6.3792E + 02	6.0776E + 02	6.0593E + 02	6.0011E + 02
	Std	2.5760E + 00	7.2877E + 00	8.7951E − 01	9.4947E + 00	9.2432E + 00	3.5449E + 00	7.1555E − 02
	Rank	2	4	3	7	6	5	1
F6	Best	7.8002E + 02	8.7720E + 02	7.7744E + 02	8.4215E + 02	8.2349E + 02	8.5221E + 02	7.3755E + 02
	Mean	8.4944E + 02	9.1222E + 02	8.1476E + 02	9.8149E + 02	9.0730E + 02	9.2740E + 02	8.7622E + 02
	Std	3.3425E + 01	1.9114E + 01	1.9271E + 01	7.7454E + 01	6.3124E + 01	2.9356E + 01	3.8522E + 01
	Rank	2	5	1	7	4	6	3
F7	Best	8.4776E + 02	9.2645E + 02	8.3475E + 02	8.8565E + 02	8.7562E + 02	8.5108E + 02	8.0954E + 02
	Mean	8.9299E + 02	9.7066E + 02	8.6629E + 02	9.3538E + 02	9.2263E + 02	8.8331E + 02	9.2462E + 02
	Std	2.9080E + 01	2.2237E + 01	1.6614E + 01	2.9142E + 01	2.5885E + 01	2.6946E + 01	5.6326E + 01
	Rank	3	7	1	6	4	2	5
F8	Best	9.0000E + 02	9.3694E + 02	9.1849E + 02	1.9594E + 03	1.3416E + 03	9.3047E + 02	9.0000E + 02
	Mean	1.0213E + 03	1.7221E + 03	1.0526E + 03	3.7405E + 03	3.2109E + 03	1.0173E + 03	9.0017E + 02
	Std	4.5338E + 02	7.2002E + 02	1.2965E + 02	1.0700E + 03	1.0937E + 03	1.1029E + 02	2.5125E − 01
	Rank	3	5	4	7	6	2	1
F9	Best	3.0658E + 03	5.8261E + 03	3.4299E + 03	3.5898E + 03	3.2537E + 03	7.6177E + 03	7.5316E + 03
	Mean	6.6245E + 03	6.4926E + 03	4.2437E + 03	5.3387E + 03	4.6202E + 03	8.4038E + 03	8.2587E + 03
	Std	1.5034E + 03	2.9803E + 02	4.9563E + 02	8.1536E + 02	6.5728E + 02	3.2512E + 02	2.7881E + 02
	Rank	5	4	1	3	2	7	6
F10	Best	1.1050E + 03	1.2366E + 03	1.1989E + 03	1.1724E + 03	1.1450E + 03	1.1699E + 03	1.1586E + 03
	Mean	1.1324E + 03	1.3140E + 03	1.2801E + 03	1.2458E + 03	1.2073E + 03	1.2729E + 03	1.1948E + 03
	Std	2.5799E + 01	5.4545E + 01	4.2880E + 01	4.7336E + 01	4.0701E + 01	3.6874E + 01	3.1249E + 01
	Rank	1	7	6	4	3	5	2
F11	Best	1.2134E + 03	5.9990E + 04	1.7053E + 06	2.9667E + 05	8.3785E + 04	2.2602E + 05	9.9675E + 04
	Mean	5.9259E + 03	4.3781E + 05	1.0968E + 07	1.5561E + 06	7.2075E + 05	2.6155E + 06	2.6421E + 05
	Std	8.2464E + 03	3.6357E + 05	9.0111E + 06	9.7778E + 05	6.4968E + 05	1.7200E + 06	1.4006E + 05
	Rank	1	3	7	5	4	6	2
F12	Best	1.3445E + 03	1.5918E + 03	4.0666E + 04	4.7579E + 03	1.4479E + 03	3.5211E + 03	3.2353E + 03
	Mean	1.1104E + 04	3.6597E + 04	1.1828E + 05	2.5774E + 04	1.0107E + 04	8.7020E + 03	8.2747E + 03
	Std	1.9693E + 04	1.8302E + 04	8.1327E + 04	2.3129E + 04	1.2793E + 04	5.3190E + 03	2.8114E + 03
	Rank	4	6	7	5	3	2	1
F13	Best	1.4363E + 03	7.3693E + 03	2.9189E + 03	1.6024E + 03	3.2584E + 03	1.9589E + 03	1.6105E + 03
	Mean	1.4503E + 03	2.9967E + 04	3.2661E + 04	2.1432E + 03	9.4956E + 03	1.0472E + 04	1.6918E + 03
	Std	8.7641E + 00	2.3039E + 04	2.5367E + 04	1.0825E + 03	6.7868E + 03	8.0811E + 03	5.2765E + 01
	Rank	1	6	7	3	4	5	2
F14	Best	1.5165E + 03	1.5789E + 03	5.2173E + 03	2.0751E + 03	1.5880E + 03	1.8023E + 03	1.7508E + 03
	Mean	2.8983E + 03	1.7367E + 04	3.5193E + 04	1.5639E + 04	8.4558E + 03	6.5005E + 03	2.6741E + 03
	Std	5.5076E + 03	1.0025E + 04	1.6569E + 04	1.1669E + 04	8.6697E + 03	5.3347E + 03	7.9073E + 02
	Rank	2	6	7	5	4	3	1
F15	Best	1.8149E + 03	2.6610E + 03	1.8440E + 03	2.0966E + 03	1.8550E + 03	1.9971E + 03	2.8130E + 03
	Mean	2.3016E + 03	3.1266E + 03	2.3816E + 03	2.7909E + 03	2.4499E + 03	2.5874E + 03	3.0482E + 03
	Std	3.3898E + 02	1.8984E + 02	2.1073E + 02	2.6120E + 02	3.0025E + 02	4.0725E + 02	1.3749E + 02
	Rank	1	7	2	5	3	4	6
F16	Best	1.7517E + 03	1.8166E + 03	1.7600E + 03	1.8346E + 03	1.7947E + 03	1.7708E + 03	1.9591E + 03
	Mean	2.0114E + 03	2.1527E + 03	1.9380E + 03	2.1920E + 03	2.0641E + 03	1.9101E + 03	2.1233E + 03
	Std	1.6424E + 02	1.7370E + 02	1.3525E + 02	2.5094E + 02	1.4351E + 02	1.0259E + 02	1.0539E + 02
	Rank	3	6	2	7	4	1	5
F17	Best	1.8264E + 03	1.2573E + 05	9.6483E + 04	1.5786E + 04	2.6692E + 04	1.2233E + 05	2.0912E + 04
	Mean	1.8318E + 03	5.7885E + 05	5.1071E + 05	7.2081E + 04	2.1363E + 05	3.3394E + 05	3.5593E + 04
	Std	1.4891E + 01	4.2539E + 05	4.0557E + 05	6.0600E + 04	2.1356E + 05	1.7963E + 05	9.5223E + 03
	Rank	1	7	6	3	4	5	2
F18	Best	1.9173E + 03	1.9786E + 03	3.1953E + 03	2.1611E + 03	1.9990E + 03	2.0601E + 03	2.1474E + 03
	Mean	2.6267E + 03	2.0098E + 04	3.6880E + 04	1.0007E + 04	1.3049E + 04	7.5037E + 03	5.3387E + 03
	Std	2.5069E + 03	1.6027E + 04	4.1988E + 04	1.1430E + 04	1.3109E + 04	6.2450E + 03	2.3411E + 03
	Rank	1	6	7	4	5	3	2
F19	Best	2.0526E + 03	2.4584E + 03	2.0997E + 03	2.2386E + 03	2.1602E + 03	2.1302E + 03	2.4319E + 03
	Mean	2.4441E + 03	2.7232E + 03	2.3739E + 03	2.4451E + 03	2.3578E + 03	2.3664E + 03	2.5863E + 03
	Std	2.5198E + 02	1.1965E + 02	1.5669E + 02	1.4128E + 02	1.4651E + 02	1.5147E + 02	9.3510E + 01
	Rank	4	7	3	5	1	2	6
F20	Best	2.3397E + 03	2.4326E + 03	2.3458E + 03	2.3810E + 03	2.2089E + 03	2.3399E + 03	2.3082E + 03
	Mean	2.3797E + 03	2.4700E + 03	2.3784E + 03	2.4444E + 03	2.3823E + 03	2.3965E + 03	2.4155E + 03
	Std	3.1585E + 01	2.2692E + 01	2.0224E + 01	4.1382E + 01	4.1824E + 01	3.4369E + 01	6.0758E + 01
	Rank	2	7	1	6	3	4	5
F21	Best	2.3000E + 03	7.1523E + 03	2.3065E + 03	2.3058E + 03	2.3001E + 03	2.3172E + 03	2.3000E + 03
	Mean	5.5608E + 03	7.7073E + 03	3.2820E + 03	3.6549E + 03	2.3008E + 03	2.3211E + 03	2.3002E + 03
	Std	2.7843E + 03	3.0644E + 02	1.6763E + 03	2.2776E + 03	1.0476E + 00	2.9761E + 00	6.1332E − 01
	Rank	6	7	4	5	2	3	1
F22	Best	2.6921E + 03	2.7734E + 03	2.6859E + 03	2.7661E + 03	2.6974E + 03	2.6975E + 03	2.6530E + 03
	Mean	2.7318E + 03	2.8320E + 03	2.7256E + 03	2.8395E + 03	2.7598E + 03	2.7703E + 03	2.7178E + 03
	Std	3.0304E + 01	2.5066E + 01	1.8817E + 01	4.8940E + 01	3.0671E + 01	3.1670E + 01	6.2891E + 01
	Rank	3	6	2	7	4	5	1
F23	Best	2.8464E + 03	2.9838E + 03	2.8541E + 03	2.9018E + 03	2.8898E + 03	2.8612E + 03	2.8258E + 03
	Mean	2.9011E + 03	3.0101E + 03	2.9025E + 03	2.9771E + 03	2.9434E + 03	2.9198E + 03	2.9281E + 03
	Std	3.6066E + 01	1.8030E + 01	2.5863E + 01	4.8847E + 01	3.3712E + 01	3.4980E + 01	6.2813E + 01
	Rank	1	7	2	6	5	3	4
F24	Best	2.8834E + 03	2.8838E + 03	2.8862E + 03	2.8841E + 03	2.8849E + 03	2.8921E + 03	2.8871E + 03
	Mean	2.8872E + 03	2.8879E + 03	2.8962E + 03	2.9070E + 03	2.9016E + 03	2.9206E + 03	2.8877E + 03
	Std	2.1901E + 00	2.1579E + 00	1.4599E + 01	2.0207E + 01	1.4822E + 01	2.0611E + 01	3.4355E − 01
	Rank	1	3	4	6	5	7	2
F25	Best	4.0280E + 03	5.1156E + 03	2.9075E + 03	2.8380E + 03	2.8015E + 03	2.8610E + 03	3.6797E + 03
	Mean	4.3714E + 03	5.5703E + 03	4.4844E + 03	5.4099E + 03	4.5780E + 03	4.5991E + 03	3.8243E + 03
	Std	2.2448E + 02	2.0210E + 02	3.7750E + 02	8.5690E + 02	1.0312E + 03	7.3758E + 02	2.6358E + 02
	Rank	2	7	3	6	4	5	1
F26	Best	3.1742E + 03	3.2132E + 03	3.2022E + 03	3.2091E + 03	3.1989E + 03	3.2371E + 03	3.1975E + 03
	Mean	3.2206E + 03	3.2391E + 03	3.2176E + 03	3.2524E + 03	3.2572E + 03	3.2764E + 03	3.2118E + 03
	Std	1.8197E + 01	1.3575E + 01	9.1648E + 00	2.9959E + 01	1.9148E + 01	1.8478E + 01	6.0569E + 00
	Rank	3	4	2	5	6	7	1
F27	Best	3.1000E + 03	3.1902E + 03	3.2255E + 03	3.2013E + 03	3.1225E + 03	3.2414E + 03	3.1977E + 03
	Mean	3.1318E + 03	3.2197E + 03	3.2703E + 03	3.2563E + 03	3.2307E + 03	3.2937E + 03	3.2110E + 03
	Std	5.4952E + 01	1.6162E + 01	3.7359E + 01	2.8210E + 01	2.9823E + 01	3.9531E + 01	1.1908E + 01
	Rank	1	3	6	5	4	7	2
F28	Best	3.2642E + 03	3.6779E + 03	3.4981E + 03	3.6039E + 03	3.4601E + 03	3.5066E + 03	3.5816E + 03
	Mean	3.4428E + 03	3.8739E + 03	3.7542E + 03	4.1510E + 03	3.7798E + 03	3.6910E + 03	3.8335E + 03
	Std	1.2868E + 02	1.2664E + 02	1.9143E + 02	2.6096E + 02	2.0075E + 02	1.6123E + 02	8.7887E + 01
	Rank	1	6	3	7	4	2	5
F29	Best	4.9516E + 03	1.1870E + 04	6.4998E + 04	8.7855E + 03	7.8723E + 03	2.7071E + 04	8.9763E + 03
	Mean	5.0560E + 03	2.7493E + 04	6.1923E + 05	4.1057E + 04	1.6487E + 04	9.3183E + 04	1.5160E + 04
	Std	6.7568E + 01	1.3012E + 04	5.5510E + 05	5.5685E + 04	8.4552E + 03	6.1468E + 04	5.1644E + 03
	Rank	1	4	7	5	3	6	2

**Table 14:** The statistics results of MSEDO and basic algorithms solving CEC2017 (D = 50).

No	Index	MSEDO	EDO	RIME	ECO	MRFO	QIO	AE
F1	Best	1.0013E + 02	4.0728E + 03	4.0942E + 06	5.0154E + 06	1.1063E + 05	5.1162E + 07	2.7238E + 03
	Mean	3.2584E + 03	6.0924E + 04	8.9937E + 06	1.6263E + 07	3.1968E + 05	8.9428E + 07	5.0636E + 03
	Std	3.6554E + 03	7.3973E + 04	3.8162E + 06	1.6940E + 07	1.5546E + 05	2.4975E + 07	1.5976E + 03
	Rank	1	3	5	6	4	7	2
F2	Best	3.0000E + 02	1.4211E + 05	2.4222E + 04	1.8507E + 04	6.2300E + 04	9.7159E + 04	1.9134E + 04
	Mean	3.0000E + 02	1.9863E + 05	3.9210E + 04	2.9561E + 04	8.7309E + 04	1.3341E + 05	2.8298E + 04
	Std	1.5309E − 06	2.7624E + 04	1.0451E + 04	7.2863E + 03	1.4925E + 04	1.7400E + 04	5.7629E + 03
	Rank	1	7	4	3	5	6	2
F3	Best	4.0000E + 02	4.3639E + 02	5.3557E + 02	5.0429E + 02	4.5184E + 02	6.0860E + 02	5.2407E + 02
	Mean	4.2879E + 02	5.4903E + 02	6.1327E + 02	6.0427E + 02	5.7759E + 02	6.8439E + 02	5.9190E + 02
	Std	4.2571E + 01	4.4080E + 01	4.2098E + 01	5.6488E + 01	5.3633E + 01	4.1570E + 01	2.9937E + 01
	Rank	1	2	6	5	3	7	4
F4	Best	5.9950E + 02	8.0275E + 02	5.8959E + 02	6.9025E + 02	6.9702E + 02	6.2420E + 02	5.3390E + 02
	Mean	6.9071E + 02	8.8399E + 02	6.5161E + 02	7.9418E + 02	7.9664E + 02	7.1130E + 02	7.4621E + 02
	Std	7.7283E + 01	3.0885E + 01	3.1434E + 01	4.7059E + 01	5.2915E + 01	4.9676E + 01	1.3392E + 02
	Rank	2	7	1	5	6	3	4
F5	Best	6.0006E + 02	6.0399E + 02	6.0333E + 02	6.4206E + 02	6.0573E + 02	6.0774E + 02	6.0004E + 02
	Mean	6.0427E + 02	6.2258E + 02	6.0648E + 02	6.5277E + 02	6.2652E + 02	6.1385E + 02	6.0018E + 02
	Std	4.0040E + 00	1.7343E + 01	2.2562E + 00	5.9100E + 00	1.1606E + 01	4.1509E + 00	1.0660E − 01
	Rank	2	5	3	7	6	4	1
F6	Best	8.6227E + 02	1.1062E + 03	8.8880E + 02	1.1024E + 03	9.7004E + 02	1.0444E + 03	7.7622E + 02
	Mean	1.0693E + 03	1.1951E + 03	9.6319E + 02	1.3251E + 03	1.1555E + 03	1.1550E + 03	1.0261E + 03
	Std	9.0640E + 01	4.8507E + 01	3.5837E + 01	1.2596E + 02	1.0712E + 02	4.1092E + 01	1.1316E + 02
	Rank	3	6	1	7	5	4	2
F7	Best	8.8557E + 02	1.0822E + 03	8.8216E + 02	9.6136E + 02	9.3635E + 02	9.4303E + 02	8.2835E + 02
	Mean	9.7726E + 02	1.1621E + 03	9.4138E + 02	1.0835E + 03	1.0854E + 03	1.0044E + 03	9.6907E + 02
	Std	7.2787E + 01	3.1966E + 01	2.5019E + 01	5.1539E + 01	7.2747E + 01	3.4748E + 01	1.3824E + 02
	Rank	3	7	1	5	6	4	2
F8	Best	9.0045E + 02	2.4541E + 03	1.2929E + 03	6.3566E + 03	6.9692E + 03	1.4217E + 03	9.0010E + 02
	Mean	1.8929E + 03	9.6788E + 03	2.4205E + 03	1.2034E + 04	1.2392E + 04	3.0953E + 03	9.0265E + 02
	Std	1.7664E + 03	3.8539E + 03	1.1650E + 03	2.9699E + 03	3.9282E + 03	1.2892E + 03	1.8581E + 00
	Rank	2	5	3	6	7	4	1
F9	Best	1.0398E + 04	1.0142E + 04	5.0340E + 03	6.4800E + 03	5.7195E + 03	1.2599E + 04	1.2999E + 04
	Mean	1.1151E + 04	1.1138E + 04	7.2050E + 03	8.5900E + 03	6.9221E + 03	1.4519E + 04	1.4424E + 04
	Std	3.1728E + 02	5.6569E + 02	7.5479E + 02	1.1849E + 03	8.0247E + 02	5.8957E + 02	4.4780E + 02
	Rank	5	4	2	3	1	7	6
F10	Best	1.1352E + 03	2.3384E + 03	1.3762E + 03	1.2560E + 03	1.2224E + 03	1.3485E + 03	1.2035E + 03
	Mean	1.2296E + 03	3.4394E + 03	1.5182E + 03	1.3887E + 03	1.3297E + 03	1.5074E + 03	1.2730E + 03
	Std	3.3379E + 01	6.5475E + 02	7.9586E + 01	7.2790E + 01	4.9108E + 01	8.2711E + 01	2.9723E + 01
	Rank	1	7	6	4	3	5	2
F11	Best	4.4898E + 03	1.9074E + 06	8.7356E + 06	4.7842E + 06	9.2330E + 05	1.2930E + 07	9.4488E + 05
	Mean	1.1372E + 04	4.8349E + 06	7.2983E + 07	1.5629E + 07	3.2195E + 06	2.2923E + 07	1.8442E + 06
	Std	1.2310E + 04	2.0424E + 06	4.3226E + 07	8.5469E + 06	1.9674E + 06	8.6180E + 06	3.4724E + 05
	Rank	1	4	7	5	3	6	2
F12	Best	1.4853E + 03	1.7861E + 03	9.8501E + 04	2.6943E + 04	1.7091E + 03	1.4239E + 04	5.5991E + 03
	Mean	6.0416E + 03	7.1538E + 03	2.2851E + 05	1.0368E + 05	7.3440E + 03	3.3914E + 04	8.6114E + 03
	Std	4.9066E + 03	6.1075E + 03	1.0842E + 05	4.9962E + 04	5.9630E + 03	1.4380E + 04	1.4337E + 03
	Rank	1	2	7	6	3	5	4
F13	Best	1.4788E + 03	2.2616E + 05	1.9494E + 04	2.1738E + 03	6.4097E + 03	3.8262E + 04	2.3399E + 03
	Mean	1.5040E + 03	4.8320E + 05	1.5931E + 05	3.9491E + 04	7.1892E + 04	1.3174E + 05	4.3126E + 03
	Std	8.0550E + 00	1.7139E + 05	9.0870E + 04	4.2010E + 04	6.0629E + 04	9.3739E + 04	1.9599E + 03
	Rank	1	7	6	3	4	5	2
F14	Best	1.6136E + 03	1.7445E + 03	2.0475E + 04	6.4458E + 03	1.6950E + 03	2.3711E + 03	1.9744E + 03
	Mean	3.2448E + 03	1.5307E + 04	8.5959E + 04	3.1271E + 04	9.2206E + 03	5.0251E + 03	2.3730E + 03
	Std	2.3423E + 03	8.1859E + 03	4.5829E + 04	1.8345E + 04	7.1836E + 03	3.1623E + 03	4.2803E + 02
	Rank	2	5	7	6	4	3	1
F15	Best	2.1484E + 03	4.0166E + 03	2.7179E + 03	2.9462E + 03	2.4310E + 03	1.9543E + 03	2.2413E + 03
	Mean	2.8262E + 03	4.5182E + 03	3.3113E + 03	3.5876E + 03	3.2781E + 03	2.8174E + 03	3.5771E + 03
	Std	4.8980E + 02	2.5510E + 02	3.7940E + 02	4.2754E + 02	4.1983E + 02	3.0530E + 02	5.0605E + 02
	Rank	2	7	4	6	3	1	5
F16	Best	2.1500E + 03	2.4026E + 03	2.2767E + 03	2.6813E + 03	2.2927E + 03	2.1402E + 03	2.7791E + 03
	Mean	2.8850E + 03	3.3866E + 03	2.8955E + 03	3.3383E + 03	2.9951E + 03	2.7795E + 03	3.4096E + 03
	Std	5.0863E + 02	2.9227E + 02	3.0382E + 02	3.2671E + 02	3.7720E + 02	2.7503E + 02	2.6266E + 02
	Rank	2	6	3	5	4	1	7
F17	Best	1.8420E + 03	5.5410E + 05	1.4321E + 05	6.2846E + 04	6.3386E + 04	2.1426E + 05	3.0516E + 04
	Mean	1.8536E + 03	2.1052E + 06	2.0388E + 06	3.7692E + 05	8.8274E + 05	1.1939E + 06	7.0679E + 04
	Std	7.8626E + 00	1.4635E + 06	1.4102E + 06	3.4184E + 05	9.5758E + 05	7.7393E + 05	2.6814E + 04
	Rank	1	7	6	3	4	5	2
F18	Best	1.9272E + 03	2.0132E + 03	3.4395E + 04	7.1826E + 03	1.9855E + 03	2.4487E + 03	7.9224E + 03
	Mean	2.3713E + 03	8.1301E + 03	2.5526E + 05	3.6568E + 04	1.4841E + 04	1.4363E + 04	1.1894E + 04
	Std	1.2144E + 03	1.0576E + 04	2.4679E + 05	1.6259E + 04	1.1284E + 04	7.9816E + 03	2.2635E + 03
	Rank	1	2	7	6	5	4	3
F19	Best	2.3849E + 03	3.2019E + 03	2.3247E + 03	2.5307E + 03	2.3469E + 03	2.3220E + 03	3.2256E + 03
	Mean	3.0903E + 03	3.6338E + 03	2.9331E + 03	3.0978E + 03	2.9374E + 03	3.2159E + 03	3.6512E + 03
	Std	3.1807E + 02	1.7678E + 02	2.9137E + 02	2.5546E + 02	2.9479E + 02	4.3567E + 02	1.3115E + 02
	Rank	3	6	1	4	2	5	7
F20	Best	2.3784E + 03	2.6312E + 03	2.3978E + 03	2.5082E + 03	2.4370E + 03	2.4368E + 03	2.3310E + 03
	Mean	2.4679E + 03	2.6817E + 03	2.4555E + 03	2.6050E + 03	2.5281E + 03	2.4990E + 03	2.5239E + 03
	Std	6.6396E + 01	3.6399E + 01	3.5462E + 01	7.9856E + 01	4.4709E + 01	3.9331E + 01	1.2150E + 02
	Rank	2	7	1	6	5	3	4
F21	Best	5.0645E + 03	1.1947E + 04	7.1354E + 03	2.3446E + 03	2.3053E + 03	2.3831E + 03	2.3043E + 03
	Mean	1.2716E + 04	1.3838E + 04	8.6816E + 03	1.0257E + 04	8.5635E + 03	5.1204E + 03	1.1029E + 04
	Std	1.4890E + 03	5.1002E + 02	8.8790E + 02	1.8897E + 03	1.9697E + 03	5.1221E + 03	6.3533E + 03
	Rank	6	7	3	4	2	1	5
F22	Best	2.7843E + 03	3.0764E + 03	2.8545E + 03	2.9635E + 03	2.8597E + 03	2.9507E + 03	2.7543E + 03
	Mean	2.8860E + 03	3.1481E + 03	2.9074E + 03	3.1189E + 03	2.9933E + 03	3.0484E + 03	2.8216E + 03
	Std	5.7869E + 01	3.1446E + 01	4.4851E + 01	9.8067E + 01	7.7754E + 01	6.3117E + 01	1.0767E + 02
	Rank	2	7	3	6	4	5	1
F23	Best	2.9958E + 03	3.2799E + 03	2.9977E + 03	3.1305E + 03	3.1158E + 03	3.1023E + 03	2.9096E + 03
	Mean	3.0790E + 03	3.3383E + 03	3.0663E + 03	3.3164E + 03	3.2096E + 03	3.1965E + 03	3.0098E + 03
	Std	8.1016E + 01	3.4741E + 01	3.5088E + 01	1.0888E + 02	6.6464E + 01	6.4632E + 01	1.2157E + 02
	Rank	3	7	2	6	5	4	1
F24	Best	2.9287E + 03	2.9882E + 03	3.0302E + 03	3.0555E + 03	3.0450E + 03	3.1166E + 03	3.0319E + 03
	Mean	3.0162E + 03	3.0582E + 03	3.0767E + 03	3.1214E + 03	3.1020E + 03	3.1743E + 03	3.0697E + 03
	Std	4.1608E + 01	2.5467E + 01	3.5373E + 01	3.7653E + 01	2.6867E + 01	3.7754E + 01	2.2321E + 01
	Rank	1	2	4	6	5	7	3
F25	Best	4.5769E + 03	7.0641E + 03	2.9306E + 03	3.3588E + 03	2.9190E + 03	3.7619E + 03	4.0030E + 03
	Mean	5.5286E + 03	7.7959E + 03	5.4178E + 03	7.6329E + 03	5.6057E + 03	6.3967E + 03	4.5166E + 03
	Std	4.8108E + 02	3.6907E + 02	6.7026E + 02	1.5626E + 03	3.2068E + 03	1.3603E + 03	7.1364E + 02
	Rank	3	7	2	6	4	5	1
F26	Best	3.2347E + 03	3.4426E + 03	3.3109E + 03	3.2851E + 03	3.3039E + 03	3.4450E + 03	3.2344E + 03
	Mean	3.3484E + 03	3.7059E + 03	3.4109E + 03	3.5747E + 03	3.5889E + 03	3.6573E + 03	3.2715E + 03
	Std	8.4625E + 01	1.6269E + 02	6.6990E + 01	1.5928E + 02	1.4470E + 02	9.4629E + 01	3.6527E + 01
	Rank	2	7	3	4	5	6	1
F27	Best	3.2534E + 03	3.2659E + 03	3.3147E + 03	3.3228E + 03	3.3044E + 03	3.4174E + 03	3.2779E + 03
	Mean	3.2811E + 03	3.3320E + 03	3.3681E + 03	3.7564E + 03	3.3797E + 03	3.5042E + 03	3.3232E + 03
	Std	2.2219E + 01	2.8095E + 01	3.5308E + 01	1.3404E + 03	4.1989E + 01	5.9588E + 01	1.7519E + 01
	Rank	1	3	4	7	5	6	2
F28	Best	3.2421E + 03	4.1706E + 03	3.6958E + 03	4.3705E + 03	3.6540E + 03	3.7721E + 03	3.7553E + 03
	Mean	3.7881E + 03	4.7416E + 03	4.3267E + 03	5.0770E + 03	4.2837E + 03	4.2574E + 03	4.4075E + 03
	Std	2.9583E + 02	4.1695E + 02	3.3820E + 02	4.0968E + 02	4.2850E + 02	3.2001E + 02	3.0526E + 02
	Rank	1	6	4	7	3	2	5
F29	Best	5.8888E + 05	2.1555E + 06	1.8459E + 07	1.8390E + 06	1.5162E + 06	5.5971E + 06	8.1430E + 05
	Mean	6.7410E + 05	3.2409E + 06	2.9521E + 07	3.5372E + 06	2.1479E + 06	8.1373E + 06	9.9018E + 05
	Std	1.0825E + 05	8.0587E + 05	7.4476E + 06	1.2685E + 06	5.5297E + 05	1.7537E + 06	9.7545E + 04
	Rank	1	4	7	5	3	6	2

**Table 15:** The statistics results of MSEDO and basic algorithms solving CEC2017 (D = 100).

No	Index	MSEDO	EDO	RIME	ECO	MRFO	QIO	AE
F1	Best	1.0913E + 02	1.5586E + 06	5.8311E + 07	1.5411E + 08	1.9143E + 07	7.5725E + 08	8.0534E + 04
	Mean	5.5156E + 03	1.0813E + 07	9.7914E + 07	4.7562E + 08	5.8840E + 07	9.7929E + 08	1.3473E + 05
	Std	5.6516E + 03	1.0848E + 07	2.6887E + 07	3.2221E + 08	3.6589E + 07	1.4752E + 08	4.8400E + 04
	Rank	1	3	5	6	4	7	2
F2	Best	3.0000E + 02	4.2148E + 05	1.6879E + 05	9.7872E + 04	1.8450E + 05	2.7605E + 05	9.2869E + 04
	Mean	3.0000E + 02	5.2850E + 05	2.2829E + 05	1.3274E + 05	2.2519E + 05	3.3825E + 05	1.1439E + 05
	Std	2.8577E − 04	5.6824E + 04	3.1423E + 04	1.7417E + 04	2.3741E + 04	2.6862E + 04	1.1974E + 04
	Rank	1	7	5	3	4	6	2
F3	Best	4.0000E + 02	7.1766E + 02	7.3673E + 02	8.4797E + 02	7.9343E + 02	1.0037E + 03	6.6685E + 02
	Mean	4.3858E + 02	8.0698E + 02	8.5483E + 02	9.9967E + 02	9.4433E + 02	1.1635E + 03	7.1318E + 02
	Std	6.1311E + 01	4.7583E + 01	4.9589E + 01	9.8631E + 01	6.2070E + 01	1.0782E + 02	2.4904E + 01
	Rank	1	3	4	6	5	7	2
F4	Best	7.3083E + 02	1.3953E + 03	7.9975E + 02	1.0636E + 03	1.0447E + 03	9.8287E + 02	6.1579E + 02
	Mean	9.9878E + 02	1.4976E + 03	9.0143E + 02	1.2240E + 03	1.2281E + 03	1.0970E + 03	9.9634E + 02
	Std	1.8964E + 02	5.4242E + 01	5.6901E + 01	7.9473E + 01	8.9004E + 01	6.2350E + 01	3.3185E + 02
	Rank	3	7	1	5	6	4	2
F5	Best	6.0185E + 02	6.2317E + 02	6.1061E + 02	6.4513E + 02	6.3374E + 02	6.2059E + 02	6.0054E + 02
	Mean	6.1496E + 02	6.4858E + 02	6.1834E + 02	6.6050E + 02	6.4933E + 02	6.3055E + 02	6.0104E + 02
	Std	8.5638E + 00	2.0334E + 01	4.1175E + 00	7.4530E + 00	7.7386E + 00	4.9834E + 00	3.7643E − 01
	Rank	2	5	3	7	6	4	1
F6	Best	1.2853E + 03	1.8401E + 03	1.2616E + 03	2.1159E + 03	1.7230E + 03	1.6947E + 03	9.5795E + 02
	Mean	1.7735E + 03	2.0744E + 03	1.4879E + 03	2.6222E + 03	2.1756E + 03	1.8208E + 03	1.4610E + 03
	Std	2.7391E + 02	1.2875E + 02	1.0785E + 02	2.7924E + 02	3.0670E + 02	6.7802E + 01	2.7398E + 02
	Rank	3	5	2	7	6	4	1
F7	Best	1.0388E + 03	1.6774E + 03	1.0870E + 03	1.3713E + 03	1.4377E + 03	1.3240E + 03	9.0862E + 02
	Mean	1.3408E + 03	1.8237E + 03	1.1884E + 03	1.5884E + 03	1.6435E + 03	1.4176E + 03	1.1677E + 03
	Std	2.4984E + 02	7.4271E + 01	5.8567E + 01	1.2821E + 02	1.1084E + 02	5.1143E + 01	3.0800E + 02
	Rank	3	7	2	5	6	4	1
F8	Best	1.3888E + 03	2.9244E + 04	4.6869E + 03	2.2976E + 04	2.1231E + 04	8.3680E + 03	9.1372E + 02
	Mean	1.0011E + 04	4.7430E + 04	1.0562E + 04	2.9195E + 04	2.7784E + 04	1.8275E + 04	9.6043E + 02
	Std	8.1509E + 03	8.8267E + 03	5.5093E + 03	3.5803E + 03	6.8676E + 03	7.0921E + 03	3.0940E + 01
	Rank	2	7	3	6	5	4	1
F9	Best	2.5350E + 04	2.6317E + 04	1.3887E + 04	1.4547E + 04	1.2289E + 04	2.1984E + 04	2.9512E + 04
	Mean	2.7362E + 04	2.7406E + 04	1.6120E + 04	1.8652E + 04	1.5814E + 04	3.0366E + 04	3.0866E + 04
	Std	6.8810E + 02	5.2420E + 02	1.1889E + 03	2.1633E + 03	1.9600E + 03	2.3589E + 03	6.4150E + 02
	Rank	4	5	2	3	1	6	7
F10	Best	1.5587E + 03	3.3086E + 04	3.4724E + 03	3.1519E + 03	1.4272E + 04	2.2898E + 04	3.6631E + 03
	Mean	1.7078E + 03	7.2993E + 04	4.1840E + 03	4.4960E + 03	2.4469E + 04	3.9297E + 04	4.6929E + 03
	Std	1.0992E + 02	1.6438E + 04	4.6972E + 02	8.6236E + 02	5.7590E + 03	9.2391E + 03	5.6254E + 02
	Rank	1	7	2	3	5	6	4
F11	Best	2.5064E + 04	6.8305E + 06	1.7585E + 08	4.2019E + 07	1.3513E + 07	1.2524E + 08	6.7073E + 06
	Mean	5.3747E + 04	3.1281E + 07	5.5025E + 08	8.8989E + 07	3.9554E + 07	2.4345E + 08	1.2892E + 07
	Std	2.0065E + 04	1.6817E + 07	2.4688E + 08	3.6541E + 07	1.6554E + 07	6.2197E + 07	3.4017E + 06
	Rank	1	3	7	5	4	6	2
F12	Best	1.4692E + 03	2.4953E + 03	1.4784E + 05	1.2863E + 04	2.6094E + 03	4.6194E + 04	1.2626E + 04
	Mean	6.8615E + 03	5.1882E + 03	5.7472E + 05	2.4955E + 04	9.4972E + 03	9.0204E + 04	1.8049E + 04
	Std	5.0932E + 03	2.8914E + 03	1.6566E + 06	1.2332E + 04	8.1315E + 03	2.6270E + 04	2.6042E + 03
	Rank	2	1	7	5	3	6	4
F13	Best	1.6446E + 03	2.1961E + 06	9.1692E + 05	1.3602E + 05	2.9790E + 05	3.6436E + 05	9.7411E + 04
	Mean	1.6828E + 03	5.4510E + 06	2.4834E + 06	4.7103E + 05	9.2209E + 05	1.0823E + 06	1.7225E + 05
	Std	1.5503E + 01	2.2058E + 06	9.1303E + 05	3.2440E + 05	3.8324E + 05	4.8796E + 05	5.8081E + 04
	Rank	1	7	6	3	4	5	2
F14	Best	1.7211E + 03	1.8750E + 03	5.6477E + 04	4.0960E + 03	1.9801E + 03	5.2701E + 03	4.3676E + 03
	Mean	4.6235E + 03	4.8927E + 03	1.2339E + 05	1.1988E + 04	5.1322E + 03	9.5661E + 03	6.7341E + 03
	Std	4.0545E + 03	3.9883E + 03	5.6918E + 04	1.2969E + 04	3.7994E + 03	3.8931E + 03	1.8006E + 03
	Rank	1	2	7	6	3	5	4
F15	Best	2.2939E + 03	4.6297E + 03	4.3429E + 03	5.0486E + 03	4.6804E + 03	3.7927E + 03	4.1901E + 03
	Mean	4.9053E + 03	8.7882E + 03	5.8414E + 03	6.4050E + 03	5.5825E + 03	5.2791E + 03	7.1651E + 03
	Std	1.8254E + 03	1.0461E + 03	8.1127E + 02	6.3387E + 02	6.5659E + 02	7.2474E + 02	1.5670E + 03
	Rank	1	7	4	5	3	2	6
F16	Best	3.0024E + 03	5.2287E + 03	4.1605E + 03	4.4199E + 03	3.4657E + 03	3.2585E + 03	4.9996E + 03
	Mean	5.0339E + 03	6.3438E + 03	5.0755E + 03	5.4174E + 03	4.7540E + 03	4.2761E + 03	6.2764E + 03
	Std	1.0975E + 03	4.0154E + 02	4.7454E + 02	5.5729E + 02	6.5591E + 02	5.2634E + 02	4.8147E + 02
	Rank	3	7	4	5	2	1	6
F17	Best	1.9330E + 03	4.1028E + 06	1.8888E + 06	2.6014E + 05	3.2469E + 05	9.1623E + 05	1.6992E + 05
	Mean	1.9559E + 03	1.4862E + 07	4.3441E + 06	6.9038E + 05	1.4717E + 06	1.9872E + 06	3.0589E + 05
	Std	1.2776E + 01	6.4769E + 06	1.7747E + 06	2.6894E + 05	7.2611E + 05	7.7524E + 05	8.2470E + 04
	Rank	1	7	6	3	4	5	2
F18	Best	1.9946E + 03	2.1666E + 03	2.6555E + 05	2.8044E + 03	2.0591E + 03	4.1461E + 03	2.5095E + 03
	Mean	8.6803E + 03	7.5659E + 03	4.2879E + 06	8.7625E + 03	5.7425E + 03	9.2471E + 03	3.1946E + 03
	Std	6.6548E + 03	6.3150E + 03	3.1510E + 06	6.4952E + 03	4.0952E + 03	5.0124E + 03	4.4180E + 02
	Rank	4	3	7	5	2	6	1
F19	Best	5.3994E + 03	5.7532E + 03	3.9966E + 03	4.3563E + 03	3.9227E + 03	4.0479E + 03	6.3832E + 03
	Mean	6.4559E + 03	6.4822E + 03	5.0868E + 03	5.3302E + 03	4.9854E + 03	6.4784E + 03	6.7777E + 03
	Std	3.3349E + 02	4.1746E + 02	4.9442E + 02	4.7541E + 02	5.6984E + 02	1.0229E + 03	1.8412E + 02
	Rank	4	6	2	3	1	5	7
F20	Best	2.5762E + 03	3.2391E + 03	2.6308E + 03	2.9000E + 03	2.7563E + 03	2.8194E + 03	2.4250E + 03
	Mean	2.8963E + 03	3.3706E + 03	2.7566E + 03	3.2185E + 03	2.9707E + 03	2.9725E + 03	2.7866E + 03
	Std	2.4950E + 02	6.9273E + 01	7.9581E + 01	1.5008E + 02	9.6051E + 01	1.0288E + 02	3.1021E + 02
	Rank	3	7	1	6	4	5	2
F21	Best	2.8692E + 04	2.7228E + 04	1.5322E + 04	1.8956E + 04	1.6321E + 04	2.6843E + 03	3.1436E + 04
	Mean	2.9926E + 04	2.8952E + 04	1.8504E + 04	2.2166E + 04	1.8898E + 04	2.2947E + 04	3.2861E + 04
	Std	4.8877E + 02	6.7121E + 02	1.2801E + 03	1.8540E + 03	1.6258E + 03	1.3552E + 04	6.4630E + 02
	Rank	6	5	1	3	2	4	7
F22	Best	3.1244E + 03	3.7857E + 03	3.1513E + 03	3.4708E + 03	3.3901E + 03	3.6536E + 03	2.9476E + 03
	Mean	3.2641E + 03	3.9575E + 03	3.2361E + 03	3.7434E + 03	3.5948E + 03	3.8092E + 03	2.9914E + 03
	Std	8.5084E + 01	6.2468E + 01	5.0307E + 01	1.4142E + 02	1.0194E + 02	9.7606E + 01	2.1882E + 01
	Rank	3	7	2	5	4	6	1
F23	Best	3.4807E + 03	4.2957E + 03	3.5775E + 03	4.1642E + 03	4.0862E + 03	4.1648E + 03	3.3577E + 03
	Mean	3.7464E + 03	4.4823E + 03	3.7230E + 03	4.4475E + 03	4.3156E + 03	4.3999E + 03	3.3969E + 03
	Std	1.4054E + 02	8.6097E + 01	6.8044E + 01	1.9161E + 02	1.7241E + 02	1.0993E + 02	2.5997E + 01
	Rank	3	7	2	6	4	5	1
F24	Best	3.1372E + 03	3.3339E + 03	3.3921E + 03	3.5784E + 03	3.4910E + 03	3.6195E + 03	3.3000E + 03
	Mean	3.2328E + 03	3.4740E + 03	3.5797E + 03	3.7090E + 03	3.6257E + 03	3.8143E + 03	3.4051E + 03
	Std	5.4325E + 01	6.6301E + 01	7.9151E + 01	7.9112E + 01	6.6125E + 01	9.7170E + 01	3.6854E + 01
	Rank	1	3	4	6	5	7	2
F25	Best	8.5685E + 03	1.2147E + 04	9.1177E + 03	1.5050E + 04	7.4637E + 03	1.2763E + 04	6.6797E + 03
	Mean	1.0285E + 04	1.7055E + 04	1.0420E + 04	1.7998E + 04	1.8897E + 04	1.5042E + 04	7.3263E + 03
	Std	1.3210E + 03	1.1837E + 03	8.3763E + 02	2.0166E + 03	3.5315E + 03	1.9215E + 03	2.6829E + 02
	Rank	2	5	3	6	7	4	1
F26	Best	3.3863E + 03	3.6509E + 03	3.4695E + 03	3.5011E + 03	3.7409E + 03	3.7390E + 03	3.3397E + 03
	Mean	3.4910E + 03	3.8614E + 03	3.5951E + 03	3.7992E + 03	3.8875E + 03	3.9302E + 03	3.3926E + 03
	Std	7.8241E + 01	1.3643E + 02	6.9800E + 01	1.7880E + 02	1.1956E + 02	9.0190E + 01	2.4649E + 01
	Rank	2	5	3	4	6	7	1
F27	Best	3.2620E + 03	3.4953E + 03	3.5531E + 03	3.5926E + 03	3.6320E + 03	3.7438E + 03	3.4344E + 03
	Mean	3.3381E + 03	3.6254E + 03	3.6536E + 03	3.7442E + 03	3.7977E + 03	3.9399E + 03	3.4753E + 03
	Std	4.9672E + 01	7.6599E + 01	5.6245E + 01	7.2996E + 01	9.8708E + 01	1.0351E + 02	1.7654E + 01
	Rank	1	3	4	5	6	7	2
F28	Best	4.1747E + 03	5.9941E + 03	5.8327E + 03	6.8130E + 03	5.9467E + 03	5.7146E + 03	5.9040E + 03
	Mean	5.7039E + 03	8.4348E + 03	7.3018E + 03	7.8147E + 03	7.0116E + 03	6.8711E + 03	7.7459E + 03
	Std	7.2025E + 02	1.1455E + 03	5.3835E + 02	5.9645E + 02	6.4379E + 02	5.2793E + 02	8.5837E + 02
	Rank	1	7	4	6	3	2	5
F29	Best	5.8090E + 03	4.1378E + 04	1.8817E + 07	1.2781E + 05	2.4307E + 04	8.9442E + 05	8.9647E + 04
	Mean	4.3415E + 04	1.8021E + 05	4.7295E + 07	4.7825E + 05	1.0681E + 05	1.8244E + 06	1.8640E + 05
	Std	1.6219E + 05	1.3755E + 05	1.6593E + 07	2.7946E + 05	7.5390E + 04	7.2162E + 05	6.2047E + 04
	Rank	1	3	7	5	2	6	4

**Table 16:** The statistics results of MSEDO and basic algorithms solving CEC2022 (D = 10).

No	Index	MSEDO	EDO	RIME	ECO	MRFO	QIO	AE
F1	Best	3.0000E + 02	8.7876E + 02	3.0003E + 02	3.0000E + 02	3.0687E + 02	3.7014E + 02	3.6336E + 02
	Mean	3.0000E + 02	2.2632E + 03	3.0093E + 02	3.0017E + 02	3.4589E + 02	6.8992E + 02	4.5810E + 02
	Std	4.3522E − 14	8.3485E + 02	9.2844E − 01	2.8774E − 01	3.6215E + 01	2.5012E + 02	9.1790E + 01
	Rank	1	7	3	2	4	6	5
F2	Best	4.0000E + 02	4.0000E + 02	4.0010E + 02	4.0000E + 02	4.0000E + 02	4.0000E + 02	4.0006E + 02
	Mean	4.0485E + 02	4.0503E + 02	4.0773E + 02	4.0893E + 02	4.0895E + 02	4.0976E + 02	4.0555E + 02
	Std	1.2778E + 01	3.3098E + 00	1.3006E + 01	1.1777E + 01	2.0607E + 01	2.0321E + 01	4.0250E + 00
	Rank	1	2	4	5	6	7	3
F3	Best	6.0000E + 02	6.0000E + 02	6.0005E + 02	6.0015E + 02	6.0000E + 02	6.0014E + 02	6.0002E + 02
	Mean	6.0056E + 02	6.0004E + 02	6.0023E + 02	6.0600E + 02	6.0006E + 02	6.0059E + 02	6.0003E + 02
	Std	1.4918E + 00	5.9272E − 02	1.6339E − 01	5.8912E + 00	2.5772E − 01	3.4657E − 01	9.0982E − 03
	Rank	5	2	4	7	3	6	1
F4	Best	8.0298E + 02	8.1318E + 02	8.0500E + 02	8.0697E + 02	8.0597E + 02	8.0307E + 02	8.0444E + 02
	Mean	8.1907E + 02	8.2380E + 02	8.1727E + 02	8.2236E + 02	8.1844E + 02	8.1540E + 02	8.2322E + 02
	Std	1.1159E + 01	5.6819E + 00	7.1954E + 00	1.0853E + 01	7.2727E + 00	7.7032E + 00	8.8875E + 00
	Rank	4	7	2	5	3	1	6
F5	Best	9.0000E + 02	9.0000E + 02	9.0001E + 02	9.0054E + 02	9.0000E + 02	9.0001E + 02	9.0000E + 02
	Mean	9.0035E + 02	9.0009E + 02	9.0021E + 02	9.7620E + 02	9.0129E + 02	9.0016E + 02	9.0000E + 02
	Std	1.1638E + 00	2.0934E − 01	2.7217E − 01	1.0343E + 02	4.7911E + 00	1.8524E − 01	5.0601E − 04
	Rank	5	2	4	7	6	3	1
F6	Best	1.8000E + 03	2.0045E + 03	1.9232E + 03	1.8429E + 03	1.8240E + 03	1.8458E + 03	1.9679E + 03
	Mean	1.8022E + 03	4.6887E + 03	3.6348E + 03	2.3752E + 03	3.2919E + 03	2.0586E + 03	2.3071E + 03
	Std	6.3734E + 00	1.8429E + 03	1.8261E + 03	1.3399E + 03	1.3804E + 03	3.0099E + 02	2.7356E + 02
	Rank	1	7	6	4	5	2	3
F7	Best	2.0000E + 03	2.0144E + 03	2.0005E + 03	2.0059E + 03	2.0015E + 03	2.0136E + 03	2.0221E + 03
	Mean	2.0149E + 03	2.0265E + 03	2.0173E + 03	2.0351E + 03	2.0141E + 03	2.0268E + 03	2.0366E + 03
	Std	2.2938E + 01	3.8551E + 00	8.3008E + 00	1.6600E + 01	8.6399E + 00	6.2885E + 00	5.8898E + 00
	Rank	2	4	3	6	1	5	7
F8	Best	2.2017E + 03	2.2113E + 03	2.2012E + 03	2.2069E + 03	2.2012E + 03	2.2137E + 03	2.2245E + 03
	Mean	2.2337E + 03	2.2228E + 03	2.2183E + 03	2.2243E + 03	2.2231E + 03	2.2267E + 03	2.2289E + 03
	Std	4.2763E + 01	3.3278E + 00	7.5104E + 00	6.9006E + 00	7.4220E + 00	4.4143E + 00	1.8376E + 00
	Rank	7	2	1	4	3	5	6
F9	Best	2.4000E + 03	2.5293E + 03	2.5293E + 03	2.5293E + 03	2.5293E + 03	2.5301E + 03	2.5293E + 03
	Mean	2.5207E + 03	2.5293E + 03	2.5293E + 03	2.5342E + 03	2.5294E + 03	2.5312E + 03	2.5293E + 03
	Std	3.2800E + 01	2.5059E − 02	4.5358E − 03	2.6826E + 01	6.2637E − 02	9.6843E − 01	3.4177E − 03
	Rank	1	4	2	7	5	6	3
F10	Best	2.4070E + 03	2.5003E + 03	2.5002E + 03	2.5003E + 03	2.5002E + 03	2.5003E + 03	2.5002E + 03
	Mean	2.5015E + 03	2.5004E + 03	2.5077E + 03	2.5284E + 03	2.5046E + 03	2.5005E + 03	2.5004E + 03
	Std	2.7193E + 01	5.7553E − 02	2.7924E + 01	5.1441E + 01	2.2893E + 01	1.1043E − 01	7.8214E − 02
	Rank	4	1	6	7	5	3	2
F11	Best	2.6000E + 03	2.6001E + 03	2.6099E + 03	2.6001E + 03	2.6000E + 03	2.6021E + 03	2.7550E + 03
	Mean	2.8487E + 03	2.8701E + 03	2.8816E + 03	2.7151E + 03	2.6970E + 03	2.6277E + 03	2.8979E + 03
	Std	1.1799E + 02	9.1419E + 01	1.3127E + 02	1.7803E + 02	1.5245E + 02	4.9151E + 01	2.7010E + 01
	Rank	4	5	6	3	2	1	7
F12	Best	2.8639E + 03	2.8602E + 03	2.8597E + 03	2.8614E + 03	2.8627E + 03	2.8639E + 03	2.8638E + 03
	Mean	2.8664E + 03	2.8643E + 03	2.8641E + 03	2.8639E + 03	2.8676E + 03	2.8671E + 03	2.8649E + 03
	Std	2.0092E + 00	1.2616E + 00	1.6810E + 00	1.3868E + 00	3.9393E + 00	1.9174E + 00	2.0063E − 01
	Rank	5	3	2	1	7	6	4

**Table 17:** The statistics results of MSEDO and basic algorithms solving CEC2022 (D = 20).

No	Index	MSEDO	EDO	RIME	ECO	MRFO	QIO	AE
F1	Best	3.0000E + 02	9.6051E + 03	3.3493E + 02	3.2946E + 02	1.7475E + 03	5.9728E + 03	1.7094E + 03
	Mean	3.0000E + 02	2.1724E + 04	3.8781E + 02	6.2205E + 02	5.6839E + 03	9.9115E + 03	2.8656E + 03
	Std	9.0394E − 08	5.6737E + 03	3.6110E + 01	3.0272E + 02	2.2734E + 03	2.5759E + 03	8.1008E + 02
	Rank	1	7	2	3	5	6	4
F2	Best	4.0000E + 02	4.4490E + 02	4.0114E + 02	4.2936E + 02	4.0128E + 02	4.5154E + 02	4.4911E + 02
	Mean	4.3884E + 02	4.5124E + 02	4.4962E + 02	4.6118E + 02	4.5087E + 02	4.6814E + 02	4.4925E + 02
	Std	1.9476E + 01	7.4382E + 00	1.6928E + 01	2.5822E + 01	1.6836E + 01	1.8290E + 01	1.2588E − 01
	Rank	1	5	3	6	4	7	2
F3	Best	6.0000E + 02	6.0010E + 02	6.0043E + 02	6.0883E + 02	6.0003E + 02	6.0118E + 02	6.0007E + 02
	Mean	6.0061E + 02	6.0066E + 02	6.0109E + 02	6.2337E + 02	6.0189E + 02	6.0283E + 02	6.0016E + 02
	Std	1.5761E + 00	7.1989E − 01	5.2992E − 01	9.8771E + 00	3.4688E + 00	1.0261E + 00	1.0616E − 01
	Rank	2	3	4	7	5	6	1
F4	Best	8.2089E + 02	8.5664E + 02	8.1818E + 02	8.4191E + 02	8.2985E + 02	8.1573E + 02	8.5004E + 02
	Mean	8.4812E + 02	8.8172E + 02	8.4313E + 02	8.7035E + 02	8.5648E + 02	8.4994E + 02	8.9028E + 02
	Std	1.8245E + 01	1.2250E + 01	1.3821E + 01	1.8988E + 01	1.5435E + 01	2.3230E + 01	1.1610E + 01
	Rank	2	6	1	5	4	3	7
F5	Best	9.0000E + 02	9.0056E + 02	9.0110E + 02	1.0249E + 03	9.0091E + 02	9.0103E + 02	9.0000E + 02
	Mean	9.4602E + 02	9.2659E + 02	9.2635E + 02	1.8896E + 03	1.3077E + 03	9.0981E + 02	9.0009E + 02
	Std	1.5860E + 02	6.5319E + 01	6.4275E + 01	4.4870E + 02	4.8346E + 02	1.0655E + 01	1.4000E − 01
	Rank	5	4	3	7	6	2	1
F6	Best	1.8002E + 03	3.3424E + 03	3.3343E + 03	2.0418E + 03	1.9694E + 03	2.3326E + 03	2.0253E + 03
	Mean	1.8111E + 03	1.5245E + 04	1.5149E + 04	1.0482E + 04	6.7608E + 03	9.1048E + 03	2.7727E + 03
	Std	2.2581E + 01	1.2646E + 04	7.4080E + 03	7.1222E + 03	4.4931E + 03	5.5442E + 03	6.7150E + 02
	Rank	1	7	6	5	3	4	2
F7	Best	2.0149E + 03	2.0463E + 03	2.0253E + 03	2.0489E + 03	2.0247E + 03	2.0432E + 03	2.0815E + 03
	Mean	2.0907E + 03	2.0753E + 03	2.0372E + 03	2.1060E + 03	2.0489E + 03	2.0597E + 03	2.0979E + 03
	Std	8.7455E + 01	1.4856E + 01	9.2467E + 00	3.5368E + 01	1.7744E + 01	1.3338E + 01	7.7018E + 00
	Rank	5	4	1	7	2	3	6
F8	Best	2.2225E + 03	2.2272E + 03	2.2222E + 03	2.2259E + 03	2.2242E + 03	2.2289E + 03	2.2298E + 03
	Mean	2.2379E + 03	2.2302E + 03	2.2280E + 03	2.2432E + 03	2.2319E + 03	2.2355E + 03	2.2372E + 03
	Std	3.5363E + 01	1.6912E + 00	3.7648E + 00	3.3297E + 01	2.2142E + 01	3.5405E + 00	3.2958E + 00
	Rank	6	2	1	7	3	4	5
F9	Best	2.4808E + 03	2.4808E + 03	2.4808E + 03	2.4808E + 03	2.4811E + 03	2.4830E + 03	2.4808E + 03
	Mean	2.4808E + 03	2.4814E + 03	2.4811E + 03	2.4809E + 03	2.4817E + 03	2.4868E + 03	2.4809E + 03
	Std	6.7826E − 07	6.0194E − 01	2.1965E − 01	1.1575E − 01	3.7736E − 01	3.0092E + 00	5.7867E − 02
	Rank	1	5	4	2	6	7	3
F10	Best	2.5002E + 03	2.5005E + 03	2.4366E + 03	2.5006E + 03	2.5004E + 03	2.5005E + 03	2.5004E + 03
	Mean	2.8539E + 03	3.8384E + 03	2.5320E + 03	2.9342E + 03	2.5117E + 03	2.5060E + 03	2.5006E + 03
	Std	5.8060E + 02	9.9703E + 02	9.4990E + 01	6.6684E + 02	4.1999E + 01	2.8112E + 01	9.4345E − 02
	Rank	5	7	4	6	3	2	1
F11	Best	2.9000E + 03	2.9000E + 03	2.9562E + 03	2.9111E + 03	2.9001E + 03	2.9351E + 03	2.9187E + 03
	Mean	2.9000E + 03	2.9005E + 03	3.0108E + 03	2.9914E + 03	2.9646E + 03	2.9560E + 03	2.9289E + 03
	Std	2.6504E − 11	5.2304E − 01	2.7854E + 01	1.1471E + 02	1.0591E + 02	1.1967E + 01	4.1663E + 00
	Rank	1	2	7	6	5	4	3
F12	Best	2.9351E + 03	2.9421E + 03	2.9357E + 03	2.9441E + 03	2.9436E + 03	2.9726E + 03	2.9342E + 03
	Mean	2.9622E + 03	2.9541E + 03	2.9521E + 03	2.9747E + 03	2.9769E + 03	2.9919E + 03	2.9446E + 03
	Std	3.0725E + 01	1.0488E + 01	8.2700E + 00	2.5387E + 01	1.8479E + 01	1.2531E + 01	5.9619E + 00
	Rank	4	3	2	5	6	7	1

**Table 18:** The statistics results of MSEDO and improved algorithms solving CEC2017 (D = 10).

No	Index	MSEDO	EDO	IRIME	EMTLBO	EOSMA	MTVSCA	APSM-jSO
F1	Best	1.0001E + 02	1.0014E + 02	1.0205E + 04	1.0691E + 02	3.0248E + 03	1.2993E + 05	1.0017E + 02
	Mean	1.0025E + 02	3.5109E + 03	4.0550E + 04	8.1397E + 02	2.5049E + 04	4.5422E + 05	1.0508E + 02
	Std	1.2520E + 00	2.7405E + 03	3.1790E + 04	8.6449E + 02	1.7962E + 04	2.8334E + 05	1.3583E + 01
	Rank	1	4	6	3	5	7	2
F2	Best	3.0000E + 02	1.0202E + 03	3.2729E + 02	3.0000E + 02	3.6337E + 02	3.2946E + 02	3.0000E + 02
	Mean	3.0000E + 02	3.1180E + 03	4.4477E + 02	3.0057E + 02	6.0548E + 02	4.4959E + 02	3.0000E + 02
	Std	9.9581E − 14	1.5322E + 03	1.2106E + 02	1.4864E + 00	1.7893E + 02	1.0654E + 02	4.0001E − 05
	Rank	1	7	4	3	6	5	2
F3	Best	4.0000E + 02	4.0243E + 02	4.0113E + 02	4.0000E + 02	4.0403E + 02	4.0342E + 02	4.0016E + 02
	Mean	4.0000E + 02	4.0477E + 02	4.0608E + 02	4.0137E + 02	4.0622E + 02	4.0551E + 02	4.0067E + 02
	Std	8.6802E − 10	7.3177E − 01	1.5949E + 00	8.7512E − 01	8.5274E − 01	8.7976E − 01	3.3692E − 01
	Rank	1	4	6	3	7	5	2
F4	Best	5.0398E + 02	5.1360E + 02	5.0400E + 02	5.0981E + 02	5.0740E + 02	5.1808E + 02	5.1214E + 02
	Mean	5.2172E + 02	5.2351E + 02	5.1095E + 02	5.2287E + 02	5.1416E + 02	5.2639E + 02	5.2218E + 02
	Std	2.3103E + 01	4.9301E + 00	3.8955E + 00	6.2810E + 00	3.2415E + 00	4.7777E + 00	5.3304E + 00
	Rank	3	6	1	5	2	7	4
F5	Best	6.0000E + 02	6.0000E + 02	6.0004E + 02	6.0000E + 02	6.0005E + 02	6.0056E + 02	6.0001E + 02
	Mean	6.0026E + 02	6.0010E + 02	6.0013E + 02	6.0000E + 02	6.0015E + 02	6.0110E + 02	6.0002E + 02
	Std	1.0890E + 00	3.0012E − 01	5.8763E − 02	8.1638E − 04	7.7838E − 02	3.4587E − 01	7.7000E − 03
	Rank	6	3	4	1	5	7	2
F6	Best	7.0533E + 02	7.2212E + 02	7.1485E + 02	7.2583E + 02	7.1753E + 02	7.2610E + 02	7.2899E + 02
	Mean	7.2163E + 02	7.3284E + 02	7.2190E + 02	7.3341E + 02	7.2520E + 02	7.4365E + 02	7.4020E + 02
	Std	9.1725E + 00	6.1432E + 00	4.3412E + 00	4.6087E + 00	5.1169E + 00	7.2382E + 00	5.2922E + 00
	Rank	1	4	2	5	3	7	6
F7	Best	8.0398E + 02	8.1182E + 02	8.0501E + 02	8.1812E + 02	8.0441E + 02	8.1909E + 02	8.1299E + 02
	Mean	8.2106E + 02	8.2262E + 02	8.1006E + 02	8.2385E + 02	8.1054E + 02	8.2932E + 02	8.2420E + 02
	Std	1.1172E + 01	4.6598E + 00	3.9939E + 00	3.4507E + 00	3.9845E + 00	5.1270E + 00	5.4153E + 00
	Rank	3	4	1	5	2	7	6
F8	Best	9.0000E + 02	9.0000E + 02	9.0001E + 02	9.0000E + 02	9.0001E + 02	9.0040E + 02	9.0000E + 02
	Mean	9.0009E + 02	9.0002E + 02	9.0011E + 02	9.0000E + 02	9.0019E + 02	9.0148E + 02	9.0000E + 02
	Std	1.9151E − 01	8.4851E − 02	9.9588E − 02	4.7410E − 07	3.3917E − 01	1.0610E + 00	1.6019E − 05
	Rank	4	3	5	1	6	7	2
F9	Best	1.1231E + 03	1.9125E + 03	1.0402E + 03	1.5009E + 03	1.1487E + 03	1.9119E + 03	1.9619E + 03
	Mean	1.7766E + 03	2.0972E + 03	1.3824E + 03	1.9506E + 03	1.8131E + 03	2.2446E + 03	2.3894E + 03
	Std	3.3407E + 02	1.0050E + 02	1.4741E + 02	2.2620E + 02	2.5059E + 02	1.6383E + 02	1.7615E + 02
	Rank	2	5	1	4	3	6	7
F10	Best	1.1000E + 03	1.1017E + 03	1.1032E + 03	1.1023E + 03	1.1020E + 03	1.1053E + 03	1.1037E + 03
	Mean	1.1058E + 03	1.1064E + 03	1.1106E + 03	1.1058E + 03	1.1069E + 03	1.1127E + 03	1.1062E + 03
	Std	4.9329E + 00	2.9064E + 00	5.0091E + 00	1.6813E + 00	2.5352E + 00	3.0254E + 00	1.3390E + 00
	Rank	1	4	6	2	5	7	3
F11	Best	1.2000E + 03	2.1599E + 04	6.6885E + 03	1.7078E + 03	2.9097E + 04	3.3775E + 03	1.2256E + 03
	Mean	1.2664E + 03	3.0129E + 05	4.7096E + 04	1.1038E + 04	1.3879E + 05	2.6483E + 04	1.4389E + 03
	Std	9.3930E + 01	3.6239E + 05	3.5952E + 04	1.2391E + 04	1.4650E + 05	1.2323E + 04	1.0064E + 02
	Rank	1	7	5	3	6	4	2
F12	Best	1.3003E + 03	1.3689E + 03	1.3298E + 03	1.3109E + 03	1.4814E + 03	1.3434E + 03	1.3058E + 03
	Mean	1.3039E + 03	5.0469E + 03	1.9937E + 03	1.5837E + 03	2.1828E + 03	1.3871E + 03	1.3123E + 03
	Std	3.3659E + 00	5.6742E + 03	6.9646E + 02	1.0410E + 03	4.9776E + 02	2.5605E + 01	3.3240E + 00
	Rank	1	7	5	4	6	3	2
F13	Best	1.4000E + 03	1.4458E + 03	1.4222E + 03	1.4033E + 03	1.4366E + 03	1.4163E + 03	1.4120E + 03
	Mean	1.4031E + 03	1.5549E + 03	1.4401E + 03	1.4291E + 03	1.4585E + 03	1.4273E + 03	1.4231E + 03
	Std	5.7833E + 00	2.2567E + 02	1.2243E + 01	1.1620E + 01	1.2233E + 01	3.1443E + 00	4.2495E + 00
	Rank	1	7	5	4	6	3	2
F14	Best	1.5000E + 03	1.5358E + 03	1.5080E + 03	1.5023E + 03	1.5417E + 03	1.5026E + 03	1.5023E + 03
	Mean	1.5014E + 03	1.7364E + 03	1.5343E + 03	1.5177E + 03	1.6086E + 03	1.5109E + 03	1.5038E + 03
	Std	5.7709E + 00	2.3632E + 02	2.2881E + 01	8.8837E + 00	4.7016E + 01	3.5207E + 00	8.5205E − 01
	Rank	1	7	5	4	6	3	2
F15	Best	1.6000E + 03	1.6036E + 03	1.6019E + 03	1.6027E + 03	1.6045E + 03	1.6102E + 03	1.6058E + 03
	Mean	1.7623E + 03	1.6352E + 03	1.6200E + 03	1.6371E + 03	1.6196E + 03	1.6490E + 03	1.6257E + 03
	Std	1.0048E + 02	3.4732E + 01	1.5310E + 01	4.7808E + 01	2.6964E + 01	2.0007E + 01	1.2732E + 01
	Rank	7	4	2	5	1	6	3
F16	Best	1.7024E + 03	1.7171E + 03	1.7017E + 03	1.7061E + 03	1.7267E + 03	1.7373E + 03	1.7382E + 03
	Mean	1.7208E + 03	1.7369E + 03	1.7170E + 03	1.7492E + 03	1.7397E + 03	1.7555E + 03	1.7543E + 03
	Std	2.7687E + 01	1.0117E + 01	1.0854E + 01	3.9521E + 01	1.0015E + 01	9.7489E + 00	9.9326E + 00
	Rank	2	3	1	5	4	7	6
F17	Best	1.8000E + 03	4.1404E + 03	1.9176E + 03	1.8070E + 03	2.4002E + 03	1.8323E + 03	1.8087E + 03
	Mean	1.8032E + 03	8.6641E + 03	5.0711E + 03	3.5999E + 03	5.1415E + 03	1.8676E + 03	1.8174E + 03
	Std	8.4084E + 00	4.6488E + 03	2.7717E + 03	6.2444E + 03	2.0079E + 03	2.3293E + 01	5.3867E + 00
	Rank	1	7	5	4	6	3	2
F18	Best	1.9000E + 03	1.9208E + 03	1.9026E + 03	1.9016E + 03	1.9172E + 03	1.9041E + 03	1.9020E + 03
	Mean	1.9015E + 03	1.9758E + 03	1.9284E + 03	1.9075E + 03	1.9553E + 03	1.9071E + 03	1.9037E + 03
	Std	6.7051E + 00	6.8381E + 01	3.7243E + 01	2.2867E + 00	2.7474E + 01	1.2184E + 00	9.8119E − 01
	Rank	1	7	5	4	6	3	2
F19	Best	2.0003E + 03	2.0243E + 03	2.0015E + 03	2.0181E + 03	2.0204E + 03	2.0356E + 03	2.0157E + 03
	Mean	2.0478E + 03	2.0436E + 03	2.0077E + 03	2.0319E + 03	2.0312E + 03	2.0512E + 03	2.0424E + 03
	Std	6.2270E + 01	9.9469E + 00	6.9248E + 00	7.6930E + 00	9.3423E + 00	8.6312E + 00	8.8439E + 00
	Rank	6	5	1	3	2	7	4
F20	Best	2.1000E + 03	2.2026E + 03	2.2001E + 03	2.1790E + 03	2.2006E + 03	2.2023E + 03	2.2000E + 03
	Mean	2.2855E + 03	2.2457E + 03	2.2253E + 03	2.2471E + 03	2.2257E + 03	2.2545E + 03	2.2741E + 03
	Std	5.9326E + 01	5.5662E + 01	4.5777E + 01	6.1522E + 01	4.5206E + 01	5.6767E + 01	6.0823E + 01
	Rank	7	3	1	4	2	5	6
F21	Best	2.3003E + 03	2.2197E + 03	2.2154E + 03	2.2116E + 03	2.2152E + 03	2.2229E + 03	2.2116E + 03
	Mean	2.3919E + 03	2.3221E + 03	2.2962E + 03	2.2973E + 03	2.2915E + 03	2.2973E + 03	2.2980E + 03
	Std	2.9812E + 02	1.4412E + 02	2.5584E + 01	1.6190E + 01	2.6826E + 01	2.6329E + 01	1.6369E + 01
	Rank	7	6	2	3	1	4	5
F22	Best	2.6055E + 03	2.6104E + 03	2.6068E + 03	2.6108E + 03	2.6060E + 03	2.6143E + 03	2.6089E + 03
	Mean	2.6162E + 03	2.6208E + 03	2.6166E + 03	2.6213E + 03	2.6130E + 03	2.6252E + 03	2.6186E + 03
	Std	6.3927E + 00	6.0258E + 00	4.7794E + 00	4.7039E + 00	4.0027E + 00	4.5240E + 00	5.4650E + 00
	Rank	2	5	3	6	1	7	4
F23	Best	2.5000E + 03	2.5092E + 03	2.5003E + 03	2.5000E + 03	2.5052E + 03	2.5213E + 03	2.5003E + 03
	Mean	2.7365E + 03	2.7101E + 03	2.6856E + 03	2.7290E + 03	2.6536E + 03	2.7116E + 03	2.7429E + 03
	Std	4.5287E + 01	8.7104E + 01	1.0705E + 02	7.6717E + 01	1.0675E + 02	8.3850E + 01	4.5988E + 01
	Rank	6	3	2	5	1	4	7
F24	Best	2.6000E + 03	2.6367E + 03	2.8978E + 03	2.8977E + 03	2.8979E + 03	2.8989E + 03	2.8977E + 03
	Mean	2.9062E + 03	2.9050E + 03	2.9221E + 03	2.9196E + 03	2.9160E + 03	2.9193E + 03	2.9166E + 03
	Std	6.2537E + 01	5.5194E + 01	2.3685E + 01	2.3327E + 01	2.0615E + 01	2.0713E + 01	2.3117E + 01
	Rank	2	1	7	6	3	5	4
F25	Best	2.8000E + 03	2.6019E + 03	2.9002E + 03	2.9000E + 03	2.8038E + 03	2.9018E + 03	2.9000E + 03
	Mean	3.3659E + 03	3.0345E + 03	2.9128E + 03	2.9016E + 03	2.8998E + 03	2.9057E + 03	2.9000E + 03
	Std	5.4672E + 02	3.8362E + 02	2.0694E + 01	8.6081E + 00	2.0018E + 01	2.9491E + 00	9.0717E − 04
	Rank	7	6	5	3	1	4	2
F26	Best	3.0889E + 03	3.0891E + 03	3.0890E + 03	3.0890E + 03	3.0895E + 03	3.0945E + 03	3.0890E + 03
	Mean	3.1003E + 03	3.0943E + 03	3.0903E + 03	3.0895E + 03	3.0908E + 03	3.0971E + 03	3.0894E + 03
	Std	1.7097E + 01	2.8280E + 00	1.0707E + 00	3.1071E − 01	1.6475E + 00	1.8498E + 00	2.2176E − 01
	Rank	7	5	3	2	4	6	1
F27	Best	3.1000E + 03	3.1000E + 03	3.1036E + 03	3.1000E + 03	3.1007E + 03	3.1104E + 03	3.1000E + 03
	Mean	3.2052E + 03	3.1300E + 03	3.1649E + 03	3.1651E + 03	3.1532E + 03	3.1494E + 03	3.2316E + 03
	Std	1.3135E + 02	3.3936E + 01	2.3416E + 01	1.2275E + 02	2.3364E + 01	2.4446E + 01	1.5283E + 02
	Rank	6	1	4	5	3	2	7
F28	Best	3.1293E + 03	3.1543E + 03	3.1354E + 03	3.1483E + 03	3.1411E + 03	3.1828E + 03	3.1613E + 03
	Mean	3.1842E + 03	3.1863E + 03	3.1575E + 03	3.1810E + 03	3.1731E + 03	3.2067E + 03	3.1876E + 03
	Std	6.6041E + 01	1.6812E + 01	1.2035E + 01	2.0323E + 01	1.8016E + 01	1.7703E + 01	1.4625E + 01
	Rank	4	5	1	3	2	7	6
F29	Best	3.3945E + 03	1.3839E + 04	3.8971E + 03	3.4288E + 03	4.8084E + 03	6.4675E + 03	3.3982E + 03
	Mean	3.0644E + 04	5.7709E + 04	7.5748E + 04	1.2472E + 05	3.8981E + 04	5.6473E + 04	8.8251E + 04
	Std	1.4920E + 05	4.5589E + 04	2.0520E + 05	2.7981E + 05	6.1430E + 04	1.0723E + 05	2.4886E + 05
	Rank	1	4	5	7	2	3	6

**Table 19:** The statistics results of MSEDO and improved algorithms solving CEC2017 (D = 30).

No	Index	MSEDO	EDO	IRIME	EMTLBO	EOSMA	MTVSCA	APSM-jSO
F1	Best	1.0003E + 02	3.0527E + 02	1.9455E + 06	1.0952E + 02	1.7380E + 06	9.5207E + 06	1.0143E + 02
	Mean	1.0148E + 02	3.0404E + 03	6.3471E + 06	4.1194E + 03	5.0245E + 06	2.0581E + 07	2.4479E + 03
	Std	4.1493E − 01	3.6727E + 03	3.6436E + 06	3.9376E + 03	2.6085E + 06	7.9304E + 06	1.5906E + 03
	Rank	1	3	6	4	5	7	2
F2	Best	3.0000E + 02	6.0706E + 04	2.3698E + 04	1.4028E + 03	2.2890E + 04	7.5232E + 03	3.0057E + 02
	Mean	3.0000E + 02	8.5772E + 04	4.0841E + 04	1.1092E + 04	3.7035E + 04	1.5136E + 04	3.0813E + 02
	Std	7.2411E − 07	1.3340E + 04	1.0114E + 04	9.7301E + 03	8.1396E + 03	3.7600E + 03	6.9358E + 00
	Rank	1	7	6	3	5	4	2
F3	Best	4.0000E + 02	4.0424E + 02	4.8891E + 02	4.0424E + 02	4.9319E + 02	4.8796E + 02	4.8434E + 02
	Mean	4.2235E + 02	4.9474E + 02	5.1259E + 02	4.8559E + 02	5.1740E + 02	5.3340E + 02	4.8730E + 02
	Std	2.9431E + 01	3.1250E + 01	1.8560E + 01	2.9083E + 01	1.4607E + 01	1.7076E + 01	5.1130E + 00
	Rank	1	4	5	2	6	7	3
F4	Best	5.4079E + 02	6.1971E + 02	5.4083E + 02	6.3977E + 02	5.5098E + 02	6.5008E + 02	6.3345E + 02
	Mean	5.8872E + 02	6.7574E + 02	5.6957E + 02	6.6974E + 02	5.8658E + 02	6.8320E + 02	6.5463E + 02
	Std	3.2737E + 01	2.0990E + 01	1.7542E + 01	1.1531E + 01	1.3620E + 01	1.0551E + 01	1.2133E + 01
	Rank	3	6	1	5	2	7	4
F5	Best	6.0000E + 02	6.0068E + 02	6.0078E + 02	6.0003E + 02	6.0111E + 02	6.0328E + 02	6.0001E + 02
	Mean	6.0197E + 02	6.0480E + 02	6.0126E + 02	6.0007E + 02	6.0186E + 02	6.0459E + 02	6.0001E + 02
	Std	2.5760E + 00	7.2877E + 00	3.4371E − 01	3.7826E − 02	7.4058E − 01	8.0502E − 01	4.5501E − 03
	Rank	5	7	3	2	4	6	1
F6	Best	7.8002E + 02	8.7720E + 02	7.8251E + 02	8.7716E + 02	8.0205E + 02	8.8659E + 02	8.5804E + 02
	Mean	8.4944E + 02	9.1222E + 02	8.2704E + 02	9.0226E + 02	8.4139E + 02	9.2709E + 02	8.8789E + 02
	Std	3.3425E + 01	1.9114E + 01	2.2101E + 01	1.2076E + 01	1.8087E + 01	1.4803E + 01	1.3220E + 01
	Rank	3	6	1	5	2	7	4
F7	Best	8.4776E + 02	9.2645E + 02	8.4231E + 02	9.3912E + 02	8.5116E + 02	9.5222E + 02	9.3104E + 02
	Mean	8.9299E + 02	9.7066E + 02	8.7485E + 02	9.6702E + 02	8.8327E + 02	9.8230E + 02	9.5325E + 02
	Std	2.9080E + 01	2.2237E + 01	2.1819E + 01	1.0947E + 01	1.9065E + 01	1.1001E + 01	1.1723E + 01
	Rank	3	6	1	5	2	7	4
F8	Best	9.0000E + 02	9.3694E + 02	9.1967E + 02	9.0000E + 02	9.1336E + 02	9.3014E + 02	9.0000E + 02
	Mean	1.0213E + 03	1.7221E + 03	9.9891E + 02	9.0265E + 02	9.5501E + 02	9.7137E + 02	9.0004E + 02
	Std	4.5338E + 02	7.2002E + 02	7.4287E + 01	3.2105E + 00	4.4261E + 01	2.8755E + 01	5.0404E − 02
	Rank	6	7	5	2	3	4	1
F9	Best	3.0658E + 03	5.8261E + 03	3.2316E + 03	6.2615E + 03	4.8739E + 03	7.4047E + 03	7.5919E + 03
	Mean	6.6245E + 03	6.4926E + 03	4.4398E + 03	7.5710E + 03	6.5564E + 03	7.8879E + 03	8.1556E + 03
	Std	1.5034E + 03	2.9803E + 02	5.5132E + 02	3.8593E + 02	5.6659E + 02	2.6352E + 02	3.2028E + 02
	Rank	4	2	1	5	3	6	7
F10	Best	1.1050E + 03	1.2366E + 03	1.1806E + 03	1.1424E + 03	1.2050E + 03	1.1982E + 03	1.1192E + 03
	Mean	1.1324E + 03	1.3140E + 03	1.2459E + 03	1.1921E + 03	1.2921E + 03	1.2565E + 03	1.1677E + 03
	Std	2.5799E + 01	5.4545E + 01	3.2330E + 01	3.0624E + 01	4.4486E + 01	3.1790E + 01	3.2513E + 01
	Rank	1	7	4	3	6	5	2
F11	Best	1.2134E + 03	5.9990E + 04	3.0297E + 05	1.5975E + 04	2.5821E + 05	5.6596E + 05	3.0061E + 03
	Mean	5.9259E + 03	4.3781E + 05	4.1116E + 06	9.2663E + 04	1.3955E + 06	2.0903E + 06	9.4382E + 03
	Std	8.2464E + 03	3.6357E + 05	2.6111E + 06	1.0606E + 05	1.4664E + 06	1.1189E + 06	5.0677E + 03
	Rank	1	4	7	3	5	6	2
F12	Best	1.3445E + 03	1.5918E + 03	7.9955E + 03	1.7602E + 03	7.4052E + 03	1.7265E + 04	1.4231E + 03
	Mean	1.1104E + 04	3.6597E + 04	4.3830E + 04	8.4102E + 03	4.5983E + 04	4.5274E + 04	1.4954E + 03
	Std	1.9693E + 04	1.8302E + 04	3.5036E + 04	1.0547E + 04	3.0503E + 04	1.9648E + 04	5.3014E + 01
	Rank	3	4	5	2	7	6	1
F13	Best	1.4363E + 03	7.3693E + 03	6.0297E + 03	1.4906E + 03	1.9949E + 03	1.5419E + 03	1.4601E + 03
	Mean	1.4503E + 03	2.9967E + 04	1.9488E + 04	4.5286E + 03	5.4228E + 03	1.5856E + 03	1.4724E + 03
	Std	8.7641E + 00	2.3039E + 04	1.2879E + 04	5.1341E + 03	2.7080E + 03	2.8309E + 01	6.8738E + 00
	Rank	1	7	6	4	5	3	2
F14	Best	1.5165E + 03	1.5789E + 03	2.8578E + 03	1.5896E + 03	8.0077E + 03	2.8950E + 03	1.5388E + 03
	Mean	2.8983E + 03	1.7367E + 04	1.1768E + 04	5.9544E + 03	2.7399E + 04	5.0899E + 03	1.5753E + 03
	Std	5.5076E + 03	1.0025E + 04	1.0729E + 04	8.7044E + 03	2.2370E + 04	1.5369E + 03	2.6464E + 01
	Rank	2	6	5	4	7	3	1
F15	Best	1.8149E + 03	2.6610E + 03	1.9113E + 03	1.9090E + 03	1.7665E + 03	2.7049E + 03	2.2697E + 03
	Mean	2.3016E + 03	3.1266E + 03	2.4887E + 03	2.9275E + 03	2.3936E + 03	3.0278E + 03	2.8859E + 03
	Std	3.3898E + 02	1.8984E + 02	2.3939E + 02	3.3903E + 02	2.7649E + 02	1.6030E + 02	2.2574E + 02
	Rank	1	7	3	5	2	6	4
F16	Best	1.7517E + 03	1.8166E + 03	1.7470E + 03	1.7852E + 03	1.8059E + 03	2.0447E + 03	1.9217E + 03
	Mean	2.0114E + 03	2.1527E + 03	2.0001E + 03	2.0407E + 03	1.9008E + 03	2.1877E + 03	2.0371E + 03
	Std	1.6424E + 02	1.7370E + 02	1.5811E + 02	1.5381E + 02	8.4186E + 01	8.4872E + 01	7.8300E + 01
	Rank	3	6	2	5	1	7	4
F17	Best	1.8264E + 03	1.2573E + 05	2.3130E + 04	2.3112E + 03	7.4623E + 04	7.5453E + 03	1.8433E + 03
	Mean	1.8318E + 03	5.7885E + 05	4.4146E + 05	7.6494E + 04	1.8756E + 05	1.8118E + 04	1.8877E + 03
	Std	1.4891E + 01	4.2539E + 05	4.6137E + 05	1.0797E + 05	9.8239E + 04	7.7314E + 03	3.2378E + 01
	Rank	1	7	6	4	5	3	2
F18	Best	1.9173E + 03	1.9786E + 03	2.2270E + 03	1.9399E + 03	2.1793E + 03	2.3043E + 03	1.9308E + 03
	Mean	2.6267E + 03	2.0098E + 04	1.3384E + 04	2.6312E + 03	1.2191E + 04	4.4668E + 03	1.9396E + 03
	Std	2.5069E + 03	1.6027E + 04	1.3687E + 04	1.5789E + 03	1.1325E + 04	2.7726E + 03	5.9716E + 00
	Rank	2	7	6	3	5	4	1
F19	Best	2.0526E + 03	2.4584E + 03	2.0651E + 03	2.1955E + 03	2.1119E + 03	2.4077E + 03	2.2883E + 03
	Mean	2.4441E + 03	2.7232E + 03	2.2838E + 03	2.5854E + 03	2.2808E + 03	2.6207E + 03	2.4812E + 03
	Std	2.5198E + 02	1.1965E + 02	1.5109E + 02	1.3582E + 02	9.2912E + 01	9.6627E + 01	9.7705E + 01
	Rank	3	7	2	5	1	6	4
F20	Best	2.3397E + 03	2.4326E + 03	2.3493E + 03	2.4313E + 03	2.3483E + 03	2.4587E + 03	2.4204E + 03
	Mean	2.3797E + 03	2.4700E + 03	2.3742E + 03	2.4569E + 03	2.3865E + 03	2.4761E + 03	2.4425E + 03
	Std	3.1585E + 01	2.2692E + 01	1.6101E + 01	1.1969E + 01	1.7716E + 01	1.0240E + 01	1.4725E + 01
	Rank	2	6	1	5	3	7	4
F21	Best	2.3000E + 03	7.1523E + 03	2.3148E + 03	2.3000E + 03	2.3125E + 03	2.3197E + 03	2.3000E + 03
	Mean	5.5608E + 03	7.7073E + 03	2.7648E + 03	2.5235E + 03	2.3164E + 03	2.3389E + 03	2.3000E + 03
	Std	2.7843E + 03	3.0644E + 02	1.1518E + 03	1.2214E + 03	3.4893E + 00	2.1177E + 01	2.5734E − 03
	Rank	6	7	5	4	2	3	1
F22	Best	2.6921E + 03	2.7734E + 03	2.7017E + 03	2.7287E + 03	2.6854E + 03	2.8085E + 03	2.7635E + 03
	Mean	2.7318E + 03	2.8320E + 03	2.7283E + 03	2.8017E + 03	2.7287E + 03	2.8381E + 03	2.7962E + 03
	Std	3.0304E + 01	2.5066E + 01	1.6120E + 01	1.9850E + 01	2.2084E + 01	1.1507E + 01	1.3198E + 01
	Rank	3	6	1	5	2	7	4
F23	Best	2.8464E + 03	2.9838E + 03	2.8636E + 03	2.9539E + 03	2.8534E + 03	2.9681E + 03	2.9366E + 03
	Mean	2.9011E + 03	3.0101E + 03	2.8968E + 03	2.9853E + 03	2.8854E + 03	3.0039E + 03	2.9622E + 03
	Std	3.6066E + 01	1.8030E + 01	2.5376E + 01	1.3924E + 01	1.8620E + 01	1.7794E + 01	1.0621E + 01
	Rank	3	7	2	5	1	6	4
F24	Best	2.8834E + 03	2.8838E + 03	2.8847E + 03	2.8835E + 03	2.8902E + 03	2.8970E + 03	2.8867E + 03
	Mean	2.8872E + 03	2.8879E + 03	2.9006E + 03	2.8890E + 03	2.9165E + 03	2.9111E + 03	2.8868E + 03
	Std	2.1901E + 00	2.1579E + 00	1.5937E + 01	1.0052E + 01	1.8144E + 01	1.1491E + 01	8.4512E − 02
	Rank	2	3	5	4	7	6	1
F25	Best	4.0280E + 03	5.1156E + 03	4.1471E + 03	2.8002E + 03	2.8620E + 03	2.9905E + 03	4.4528E + 03
	Mean	4.3714E + 03	5.5703E + 03	4.4222E + 03	4.6266E + 03	4.0574E + 03	5.2752E + 03	4.8500E + 03
	Std	2.2448E + 02	2.0210E + 02	1.6268E + 02	8.5764E + 02	5.2247E + 02	5.9747E + 02	1.7015E + 02
	Rank	2	7	3	4	1	6	5
F26	Best	3.1742E + 03	3.2132E + 03	3.2023E + 03	3.1726E + 03	3.2091E + 03	3.2404E + 03	3.1895E + 03
	Mean	3.2206E + 03	3.2391E + 03	3.2137E + 03	3.2051E + 03	3.2243E + 03	3.2628E + 03	3.2035E + 03
	Std	1.8197E + 01	1.3575E + 01	5.5233E + 00	1.3068E + 01	9.8919E + 00	1.2719E + 01	7.3106E + 00
	Rank	4	6	3	2	5	7	1
F27	Best	3.1000E + 03	3.1902E + 03	3.2268E + 03	3.1897E + 03	3.2167E + 03	3.2360E + 03	3.1801E + 03
	Mean	3.1318E + 03	3.2197E + 03	3.2731E + 03	3.2114E + 03	3.2554E + 03	3.2760E + 03	3.2106E + 03
	Std	5.4952E + 01	1.6162E + 01	4.1133E + 01	1.5649E + 01	2.6295E + 01	2.0956E + 01	1.5404E + 01
	Rank	1	4	6	3	5	7	2
F28	Best	3.2642E + 03	3.6779E + 03	3.3473E + 03	3.4942E + 03	3.5229E + 03	3.8489E + 03	3.6202E + 03
	Mean	3.4428E + 03	3.8739E + 03	3.6013E + 03	3.7913E + 03	3.7050E + 03	4.0933E + 03	3.7689E + 03
	Std	1.2868E + 02	1.2664E + 02	1.6099E + 02	1.7508E + 02	1.2254E + 02	1.3490E + 02	9.3808E + 01
	Rank	1	6	2	5	3	7	4
F29	Best	4.9516E + 03	1.1870E + 04	1.4755E + 04	5.6042E + 03	5.7801E + 04	2.9880E + 04	5.1123E + 03
	Mean	5.0560E + 03	2.7493E + 04	4.0645E + 04	1.1587E + 04	2.9813E + 05	8.9644E + 04	5.5411E + 03
	Std	6.7568E + 01	1.3012E + 04	2.7513E + 04	5.6530E + 03	2.5836E + 05	3.3241E + 04	3.1146E + 02
	Rank	1	4	5	3	7	6	2

**Table 20:** The statistics results of MSEDO and improved algorithms solving CEC2017 (D = 50).

No	Index	MSEDO	EDO	IRIME	EMTLBO	EOSMA	MTVSCA	APSM-jSO
F1	Best	1.0013E + 02	4.0728E + 03	1.9929E + 07	1.1865E + 03	3.4077E + 07	9.4498E + 07	1.2004E + 02
	Mean	3.2584E + 03	6.0924E + 04	4.6944E + 07	6.3506E + 03	7.3289E + 07	1.9989E + 08	2.3612E + 03
	Std	3.6554E + 03	7.3973E + 04	1.5287E + 07	5.8938E + 03	2.8295E + 07	7.5531E + 07	2.1902E + 03
	Rank	2	4	5	3	6	7	1
F2	Best	3.0000E + 02	1.4211E + 05	8.0547E + 04	1.4572E + 04	7.2343E + 04	3.1296E + 04	5.7612E + 02
	Mean	3.0000E + 02	1.9863E + 05	1.3524E + 05	3.6104E + 04	9.7762E + 04	5.2371E + 04	1.5275E + 03
	Std	1.5309E − 06	2.7624E + 04	2.7300E + 04	1.5530E + 04	1.2763E + 04	9.8659E + 03	6.8718E + 02
	Rank	1	7	6	3	5	4	2
F3	Best	4.0000E + 02	4.3639E + 02	4.9994E + 02	4.6901E + 02	5.5993E + 02	5.9580E + 02	4.3961E + 02
	Mean	4.2879E + 02	5.4903E + 02	6.1934E + 02	5.5832E + 02	6.4396E + 02	6.8608E + 02	5.3068E + 02
	Std	4.2571E + 01	4.4080E + 01	4.7821E + 01	4.8896E + 01	4.5426E + 01	3.6904E + 01	4.6253E + 01
	Rank	1	3	5	4	6	7	2
F4	Best	5.9950E + 02	8.0275E + 02	5.9651E + 02	8.3843E + 02	6.0478E + 02	8.3929E + 02	7.5731E + 02
	Mean	6.9071E + 02	8.8399E + 02	6.5671E + 02	8.6159E + 02	6.8570E + 02	8.7158E + 02	8.0446E + 02
	Std	7.7283E + 01	3.0885E + 01	2.7314E + 01	1.2855E + 01	3.3241E + 01	1.1646E + 01	1.4387E + 01
	Rank	3	7	1	5	2	6	4
F5	Best	6.0006E + 02	6.0399E + 02	6.0178E + 02	6.0019E + 02	6.0229E + 02	6.0485E + 02	6.0001E + 02
	Mean	6.0427E + 02	6.2258E + 02	6.0308E + 02	6.0033E + 02	6.0519E + 02	6.0745E + 02	6.0004E + 02
	Std	4.0040E + 00	1.7343E + 01	5.9598E − 01	1.3674E − 01	1.1794E + 00	1.2568E + 00	2.3045E-02
	Rank	4	7	3	2	5	6	1
F6	Best	8.6227E + 02	1.1062E + 03	9.1133E + 02	1.0490E + 03	9.1785E + 02	1.0992E + 03	1.0210E + 03
	Mean	1.0693E + 03	1.1951E + 03	9.8933E + 02	1.1086E + 03	9.9490E + 02	1.1454E + 03	1.0532E + 03
	Std	9.0640E + 01	4.8507E + 01	3.8783E + 01	2.0819E + 01	3.6740E + 01	2.2936E + 01	1.4845E + 01
	Rank	4	7	1	5	2	6	3
F7	Best	8.8557E + 02	1.0822E + 03	9.1288E + 02	1.1285E + 03	9.3901E + 02	1.1251E + 03	1.0684E + 03
	Mean	9.7726E + 02	1.1621E + 03	9.6598E + 02	1.1535E + 03	9.9201E + 02	1.1685E + 03	1.1002E + 03
	Std	7.2787E + 01	3.1966E + 01	3.2391E + 01	1.4161E + 01	3.7618E + 01	1.7050E + 01	1.6027E + 01
	Rank	2	6	1	5	3	7	4
F8	Best	9.0045E + 02	2.4541E + 03	1.1130E + 03	9.0348E + 02	1.1075E + 03	1.2851E + 03	9.0000E + 02
	Mean	1.8929E + 03	9.6788E + 03	1.7773E + 03	9.1611E + 02	1.5384E + 03	1.5978E + 03	9.0079E + 02
	Std	1.7664E + 03	3.8539E + 03	4.6776E + 02	1.1670E + 01	3.0399E + 02	2.4255E + 02	5.9370E − 01
	Rank	6	7	5	2	3	4	1
F9	Best	1.0398E + 04	1.0142E + 04	6.1121E + 03	1.0044E + 04	1.0020E + 04	1.3006E + 04	1.3312E + 04
	Mean	1.1151E + 04	1.1138E + 04	7.6974E + 03	1.3410E + 04	1.1677E + 04	1.3987E + 04	1.4165E + 04
	Std	3.1728E + 02	5.6569E + 02	7.7645E + 02	7.2513E + 02	8.7228E + 02	4.1155E + 02	3.9165E + 02
	Rank	3	2	1	5	4	6	7
F10	Best	1.1352E + 03	2.3384E + 03	1.3209E + 03	1.2617E + 03	1.4898E + 03	1.4454E + 03	1.1582E + 03
	Mean	1.2296E + 03	3.4394E + 03	1.4959E + 03	1.3200E + 03	1.6589E + 03	1.5203E + 03	1.1921E + 03
	Std	3.3379E + 01	6.5475E + 02	8.9650E + 01	3.9489E + 01	1.1864E + 02	5.2312E + 01	2.7810E + 01
	Rank	2	7	4	3	6	5	1
F11	Best	4.4898E + 03	1.9074E + 06	4.5701E + 06	9.1654E + 04	3.0669E + 06	7.3367E + 06	2.0120E + 04
	Mean	1.1372E + 04	4.8349E + 06	3.8998E + 07	1.0422E + 06	1.5294E + 07	2.0338E + 07	2.0513E + 05
	Std	1.2310E + 04	2.0424E + 06	1.9458E + 07	7.9235E + 05	9.6516E + 06	8.4642E + 06	1.6267E + 05
	Rank	1	4	7	3	5	6	2
F12	Best	1.4853E + 03	1.7861E + 03	4.5630E + 04	1.6597E + 03	2.7483E + 04	5.3425E + 04	2.4362E + 03
	Mean	6.0416E + 03	7.1538E + 03	1.1080E + 05	7.4310E + 03	7.2014E + 04	1.6265E + 05	3.0472E + 03
	Std	4.9066E + 03	6.1075E + 03	5.2680E + 04	6.0699E + 03	3.7010E + 04	1.1017E + 05	4.0682E + 02
	Rank	2	3	6	4	5	7	1
F13	Best	1.4788E + 03	2.2616E + 05	2.5519E + 04	1.6959E + 03	1.0697E + 04	1.8421E + 03	1.5235E + 03
	Mean	1.5040E + 03	4.8320E + 05	1.8345E + 05	2.0144E + 04	5.5242E + 04	2.5282E + 03	1.5581E + 03
	Std	8.0550E + 00	1.7139E + 05	1.1749E + 05	2.8198E + 04	3.2210E + 04	1.1347E + 03	1.6320E + 01
	Rank	1	7	6	4	5	3	2
F14	Best	1.6136E + 03	1.7445E + 03	9.2481E + 03	1.6900E + 03	5.8809E + 03	8.3216E + 03	1.7119E + 03
	Mean	3.2448E + 03	1.5307E + 04	2.4886E + 04	7.6466E + 03	1.7502E + 04	1.8225E + 04	1.8295E + 03
	Std	2.3423E + 03	8.1859E + 03	1.0524E + 04	6.6363E + 03	7.9513E + 03	6.0351E + 03	5.9797E + 01
	Rank	2	4	7	3	5	6	1
F15	Best	2.1484E + 03	4.0166E + 03	2.3984E + 03	2.2550E + 03	2.0987E + 03	3.8465E + 03	3.3840E + 03
	Mean	2.8262E + 03	4.5182E + 03	3.2774E + 03	4.0711E + 03	3.1023E + 03	4.4559E + 03	4.0132E + 03
	Std	4.8980E + 02	2.5510E + 02	4.1325E + 02	4.2804E + 02	4.3126E + 02	2.6405E + 02	2.9082E + 02
	Rank	1	7	3	5	2	6	4
F16	Best	2.1500E + 03	2.4026E + 03	2.3399E + 03	2.4751E + 03	2.1647E + 03	3.0585E + 03	2.8711E + 03
	Mean	2.8850E + 03	3.3866E + 03	2.8427E + 03	3.1535E + 03	2.6824E + 03	3.5709E + 03	3.2575E + 03
	Std	5.0863E + 02	2.9227E + 02	2.9510E + 02	4.2689E + 02	2.7990E + 02	1.6519E + 02	1.7410E + 02
	Rank	3	6	2	4	1	7	5
F17	Best	1.8420E + 03	5.5410E + 05	2.7961E + 05	1.5201E + 04	9.8933E + 04	2.2027E + 04	1.9273E + 03
	Mean	1.8536E + 03	2.1052E + 06	1.5024E + 06	2.4777E + 05	6.3429E + 05	6.0812E + 04	2.1150E + 03
	Std	7.8626E + 00	1.4635E + 06	1.2321E + 06	2.4200E + 05	3.5229E + 05	1.8325E + 04	9.6580E + 01
	Rank	1	7	6	4	5	3	2
F18	Best	1.9272E + 03	2.0132E + 03	3.0036E + 03	2.0394E + 03	4.0573E + 03	6.0730E + 03	1.9426E + 03
	Mean	2.3713E + 03	8.1301E + 03	1.6326E + 04	5.0344E + 03	1.4690E + 04	1.9672E + 04	1.9812E + 03
	Std	1.2144E + 03	1.0576E + 04	1.2996E + 04	5.3108E + 03	8.1726E + 03	9.1949E + 03	2.0624E + 01
	Rank	2	4	6	3	5	7	1
F19	Best	2.3849E + 03	3.2019E + 03	2.3970E + 03	3.3621E + 03	2.4583E + 03	3.5347E + 03	3.1523E + 03
	Mean	3.0903E + 03	3.6338E + 03	2.8762E + 03	3.6429E + 03	2.8467E + 03	3.7209E + 03	3.5465E + 03
	Std	3.1807E + 02	1.7678E + 02	2.7886E + 02	1.1204E + 02	1.8494E + 02	9.6887E + 01	1.9697E + 02
	Rank	3	5	2	6	1	7	4
F20	Best	2.3784E + 03	2.6312E + 03	2.3797E + 03	2.6048E + 03	2.4064E + 03	2.6064E + 03	2.5692E + 03
	Mean	2.4679E + 03	2.6817E + 03	2.4540E + 03	2.6424E + 03	2.4714E + 03	2.6623E + 03	2.6082E + 03
	Std	6.6396E + 01	3.6399E + 01	2.8553E + 01	1.4914E + 01	2.6217E + 01	1.8718E + 01	1.3827E + 01
	Rank	2	7	1	5	3	6	4
F21	Best	5.0645E + 03	1.1947E + 04	7.9953E + 03	3.0214E + 03	2.3478E + 03	5.1411E + 03	2.3036E + 03
	Mean	1.2716E + 04	1.3838E + 04	9.2551E + 03	1.3709E + 04	1.0577E + 04	1.4778E + 04	1.4107E + 04
	Std	1.4890E + 03	5.1002E + 02	7.8229E + 02	3.3506E + 03	4.6513E + 03	2.5672E + 03	4.0170E + 03
	Rank	3	5	1	4	2	7	6
F22	Best	2.7843E + 03	3.0764E + 03	2.8464E + 03	3.0130E + 03	2.8515E + 03	3.0700E + 03	2.9915E + 03
	Mean	2.8860E + 03	3.1481E + 03	2.9049E + 03	3.0571E + 03	2.9082E + 03	3.1168E + 03	3.0224E + 03
	Std	5.7869E + 01	3.1446E + 01	2.9077E + 01	2.3349E + 01	2.9642E + 01	1.9400E + 01	1.1848E + 01
	Rank	1	7	2	5	3	6	4
F23	Best	2.9958E + 03	3.2799E + 03	3.0124E + 03	3.1574E + 03	2.9867E + 03	3.2346E + 03	3.1578E + 03
	Mean	3.0790E + 03	3.3383E + 03	3.0731E + 03	3.2320E + 03	3.0399E + 03	3.2688E + 03	3.1844E + 03
	Std	8.1016E + 01	3.4741E + 01	3.9672E + 01	2.1948E + 01	2.7757E + 01	2.0994E + 01	1.3958E + 01
	Rank	3	7	2	5	1	6	4
F24	Best	2.9287E + 03	2.9882E + 03	3.0697E + 03	2.9908E + 03	3.0794E + 03	3.1145E + 03	2.9815E + 03
	Mean	3.0162E + 03	3.0582E + 03	3.1344E + 03	3.0629E + 03	3.1564E + 03	3.1613E + 03	3.0096E + 03
	Std	4.1608E + 01	2.5467E + 01	3.9313E + 01	2.9152E + 01	4.4993E + 01	2.6035E + 01	2.8832E + 01
	Rank	2	3	5	4	6	7	1
F25	Best	4.5769E + 03	7.0641E + 03	3.2119E + 03	2.9141E + 03	4.7029E + 03	6.7932E + 03	4.3535E + 03
	Mean	5.5286E + 03	7.7959E + 03	5.3289E + 03	5.9727E + 03	5.3690E + 03	7.4335E + 03	6.2053E + 03
	Std	4.8108E + 02	3.6907E + 02	5.7552E + 02	1.0576E + 03	3.8159E + 02	2.3590E + 02	4.4318E + 02
	Rank	3	7	1	4	2	6	5
F26	Best	3.2347E + 03	3.4426E + 03	3.2805E + 03	3.2367E + 03	3.3346E + 03	3.4472E + 03	3.2037E + 03
	Mean	3.3484E + 03	3.7059E + 03	3.3949E + 03	3.3071E + 03	3.4068E + 03	3.5905E + 03	3.2360E + 03
	Std	8.4625E + 01	1.6269E + 02	7.7771E + 01	6.8178E + 01	4.2624E + 01	7.0951E + 01	1.7738E + 01
	Rank	3	7	4	2	5	6	1
F27	Best	3.2534E + 03	3.2659E + 03	3.3048E + 03	3.2632E + 03	3.3725E + 03	3.4025E + 03	3.2591E + 03
	Mean	3.2811E + 03	3.3320E + 03	3.3886E + 03	3.3186E + 03	3.4798E + 03	3.5006E + 03	3.2675E + 03
	Std	2.2219E + 01	2.8095E + 01	3.5975E + 01	2.7233E + 01	6.6558E + 01	6.6860E + 01	1.6806E + 01
	Rank	2	4	5	3	6	7	1
F28	Best	3.2421E + 03	4.1706E + 03	3.4347E + 03	3.5558E + 03	3.7442E + 03	4.4608E + 03	3.9731E + 03
	Mean	3.7881E + 03	4.7416E + 03	3.9879E + 03	4.3356E + 03	4.1706E + 03	5.1490E + 03	4.2968E + 03
	Std	2.9583E + 02	4.1695E + 02	2.8650E + 02	5.0534E + 02	2.6266E + 02	2.2140E + 02	1.6947E + 02
	Rank	1	6	2	5	3	7	4
F29	Best	5.8888E + 05	2.1555E + 06	1.1284E + 06	6.8549E + 05	3.2597E + 06	6.5816E + 06	6.0194E + 05
	Mean	6.7410E + 05	3.2409E + 06	1.7978E + 06	1.0208E + 06	7.3756E + 06	1.3257E + 07	7.3520E + 05
	Std	1.0825E + 05	8.0587E + 05	3.9293E + 05	2.3229E + 05	3.1617E + 06	2.9488E + 06	1.0419E + 05
	Rank	1	5	4	3	6	7	2

**Table 21:** The statistics results of MSEDO and improved algorithms solving CEC2017 (D = 100).

No	Index	MSEDO	EDO	IRIME	EMTLBO	EOSMA	MTVSCA	APSM-jSO
F1	Best	1.0913E + 02	1.5586E + 06	3.1256E + 08	2.5725E + 05	5.8682E + 08	1.2723E + 09	1.6661E + 03
	Mean	5.5156E + 03	1.0813E + 07	6.8832E + 08	1.0042E + 06	1.3789E + 09	2.0768E + 09	7.3175E + 03
	Std	5.6516E + 03	1.0848E + 07	1.9380E + 08	5.7194E + 05	4.6942E + 08	4.0201E + 08	4.7919E + 03
	Rank	1	4	5	3	6	7	2
F2	Best	3.0000E + 02	4.2148E + 05	2.8337E + 05	7.9135E + 04	2.5109E + 05	1.2806E + 05	1.8131E + 04
	Mean	3.0000E + 02	5.2850E + 05	3.3700E + 05	1.5124E + 05	3.0517E + 05	1.5750E + 05	2.6257E + 04
	Std	2.8577E − 04	5.6824E + 04	3.6392E + 04	4.4020E + 04	3.1897E + 04	1.9416E + 04	4.9688E + 03
	Rank	1	7	6	3	5	4	2
F3	Best	4.0000E + 02	7.1766E + 02	8.1094E + 02	6.6176E + 02	9.3832E + 02	1.0226E + 03	6.0017E + 02
	Mean	4.3858E + 02	8.0698E + 02	1.0126E + 03	7.5216E + 02	1.1643E + 03	1.1519E + 03	6.3953E + 02
	Std	6.1311E + 01	4.7583E + 01	8.8872E + 01	5.3852E + 01	1.1289E + 02	6.9424E + 01	3.3330E + 01
	Rank	1	4	5	3	7	6	2
F4	Best	7.3083E + 02	1.3953E + 03	8.9376E + 02	7.5771E + 02	9.3486E + 02	1.3908E + 03	1.1399E + 03
	Mean	9.9878E + 02	1.4976E + 03	9.9350E + 02	1.3630E + 03	1.0463E + 03	1.4228E + 03	1.2019E + 03
	Std	1.8964E + 02	5.4242E + 01	5.2227E + 01	1.2013E + 02	5.6044E + 01	2.2065E + 01	2.8436E + 01
	Rank	2	7	1	5	3	6	4
F5	Best	6.0185E + 02	6.2317E + 02	6.1026E + 02	6.0146E + 02	6.1045E + 02	6.1205E + 02	6.0010E + 02
	Mean	6.1496E + 02	6.4858E + 02	6.1277E + 02	6.0284E + 02	6.1539E + 02	6.1457E + 02	6.0030E + 02
	Std	8.5638E + 00	2.0334E + 01	1.7154E + 00	8.4588E − 01	2.8878E + 00	1.5665E + 00	1.1959E − 01
	Rank	5	7	3	2	6	4	1
F6	Best	1.2853E + 03	1.8401E + 03	1.3449E + 03	1.6294E + 03	1.4820E + 03	1.6883E + 03	1.4933E + 03
	Mean	1.7735E + 03	2.0744E + 03	1.5296E + 03	1.7157E + 03	1.5772E + 03	1.8057E + 03	1.5308E + 03
	Std	2.7391E + 02	1.2875E + 02	8.4432E + 01	3.3968E + 01	9.0680E + 01	4.4974E + 01	1.8926E + 01
	Rank	5	7	1	4	3	6	2
F7	Best	1.0388E + 03	1.6774E + 03	1.1821E + 03	1.6148E + 03	1.2298E + 03	1.6400E + 03	1.4500E + 03
	Mean	1.3408E + 03	1.8237E + 03	1.2846E + 03	1.6772E + 03	1.3470E + 03	1.7187E + 03	1.5112E + 03
	Std	2.4984E + 02	7.4271E + 01	5.6702E + 01	3.6346E + 01	5.5532E + 01	4.2276E + 01	2.3552E + 01
	Rank	2	7	1	5	3	6	4
F8	Best	1.3888E + 03	2.9244E + 04	6.1615E + 03	1.0442E + 03	5.7756E + 03	4.4994E + 03	9.0452E + 02
	Mean	1.0011E + 04	4.7430E + 04	1.2937E + 04	1.4702E + 03	1.2026E + 04	7.1748E + 03	9.1976E + 02
	Std	8.1509E + 03	8.8267E + 03	3.8270E + 03	7.5481E + 02	3.6882E + 03	1.7339E + 03	8.2404E + 00
	Rank	4	7	6	2	5	3	1
F9	Best	2.5350E + 04	2.6317E + 04	1.4537E + 04	2.8202E + 04	1.9969E + 04	2.8674E + 04	2.9031E + 04
	Mean	2.7362E + 04	2.7406E + 04	1.7793E + 04	2.9267E + 04	2.4660E + 04	3.0372E + 04	3.0377E + 04
	Std	6.8810E + 02	5.2420E + 02	1.6433E + 03	4.3328E + 02	2.0912E + 03	5.8181E + 02	5.5518E + 02
	Rank	3	4	1	5	2	6	7
F10	Best	1.5587E + 03	3.3086E + 04	1.2695E + 04	2.1461E + 03	1.3628E + 04	5.8003E + 03	1.7692E + 03
	Mean	1.7078E + 03	7.2993E + 04	2.1610E + 04	4.0418E + 03	2.2993E + 04	7.8533E + 03	2.0312E + 03
	Std	1.0992E + 02	1.6438E + 04	6.5649E + 03	2.7820E + 03	5.5514E + 03	1.2437E + 03	1.4224E + 02
	Rank	1	7	5	3	6	4	2
F11	Best	2.5064E + 04	6.8305E + 06	7.1841E + 07	3.1922E + 06	5.6125E + 07	1.5580E + 08	8.8006E + 05
	Mean	5.3747E + 04	3.1281E + 07	2.8352E + 08	8.5528E + 06	1.7374E + 08	2.4317E + 08	1.9499E + 06
	Std	2.0065E + 04	1.6817E + 07	1.0795E + 08	5.1454E + 06	8.6863E + 07	7.3814E + 07	8.6653E + 05
	Rank	1	4	7	3	5	6	2
F12	Best	1.4692E + 03	2.4953E + 03	1.6318E + 05	2.0426E + 03	3.2045E + 04	9.7697E + 04	7.1548E + 03
	Mean	6.8615E + 03	5.1882E + 03	4.4893E + 05	6.0319E + 03	6.4659E + 04	1.8975E + 05	9.8050E + 03
	Std	5.0932E + 03	2.8914E + 03	2.2286E + 05	3.4184E + 03	2.0313E + 04	9.6047E + 04	1.8417E + 03
	Rank	3	1	7	2	5	6	4
F13	Best	1.6446E + 03	2.1961E + 06	5.3366E + 05	5.4432E + 04	5.1454E + 05	2.8857E + 04	1.6484E + 03
	Mean	1.6828E + 03	5.4510E + 06	2.0283E + 06	2.9349E + 05	1.1068E + 06	7.5514E + 04	1.7509E + 03
	Std	1.5503E + 01	2.2058E + 06	1.2223E + 06	2.4593E + 05	3.4758E + 05	3.7130E + 04	6.2761E + 01
	Rank	1	7	6	4	5	3	2
F14	Best	1.7211E + 03	1.8750E + 03	2.0781E + 04	1.8209E + 03	1.8311E + 04	2.4936E + 04	2.2031E + 03
	Mean	4.6235E + 03	4.8927E + 03	7.2323E + 04	4.4201E + 03	3.4441E + 04	3.9034E + 04	2.4244E + 03
	Std	4.0545E + 03	3.9883E + 03	3.5799E + 04	2.6426E + 03	1.2090E + 04	9.3190E + 03	1.3553E + 02
	Rank	3	4	7	2	5	6	1
F15	Best	2.2939E + 03	4.6297E + 03	4.8043E + 03	3.4921E + 03	4.3559E + 03	8.4630E + 03	7.1336E + 03
	Mean	4.9053E + 03	8.7882E + 03	5.8661E + 03	7.5153E + 03	5.6423E + 03	9.1550E + 03	8.0022E + 03
	Std	1.8254E + 03	1.0461E + 03	5.9112E + 02	1.8054E + 03	7.7914E + 02	2.5475E + 02	3.9823E + 02
	Rank	1	6	3	4	2	7	5
F16	Best	3.0024E + 03	5.2287E + 03	3.4842E + 03	3.3730E + 03	3.6098E + 03	6.1536E + 03	5.1965E + 03
	Mean	5.0339E + 03	6.3438E + 03	4.6703E + 03	4.8641E + 03	4.5880E + 03	6.5402E + 03	5.9881E + 03
	Std	1.0975E + 03	4.0154E + 02	4.7190E + 02	1.2870E + 03	5.0036E + 02	2.0559E + 02	3.1363E + 02
	Rank	4	6	2	3	1	7	5
F17	Best	1.9330E + 03	4.1028E + 06	7.6028E + 05	8.3418E + 04	5.3713E + 05	1.1501E + 05	2.7133E + 03
	Mean	1.9559E + 03	1.4862E + 07	2.6999E + 06	4.0762E + 05	1.5561E + 06	1.8335E + 05	4.6566E + 03
	Std	1.2776E + 01	6.4769E + 06	1.1106E + 06	2.4586E + 05	7.1023E + 05	5.3822E + 04	1.4695E + 03
	Rank	1	7	6	4	5	3	2
F18	Best	1.9946E + 03	2.1666E + 03	1.8775E + 04	2.1244E + 03	6.5657E + 03	3.8061E + 04	2.1138E + 03
	Mean	8.6803E + 03	7.5659E + 03	9.2854E + 04	6.2438E + 03	2.0788E + 04	1.0720E + 05	2.1674E + 03
	Std	6.6548E + 03	6.3150E + 03	7.0580E + 04	4.6681E + 03	1.1810E + 04	5.4700E + 04	4.4855E + 01
	Rank	4	3	6	2	5	7	1
F19	Best	5.3994E + 03	5.7532E + 03	3.7542E + 03	6.5481E + 03	4.1010E + 03	6.1592E + 03	5.9127E + 03
	Mean	6.4559E + 03	6.4822E + 03	4.6444E + 03	6.9490E + 03	4.8735E + 03	6.9539E + 03	6.7496E + 03
	Std	3.3349E + 02	4.1746E + 02	5.0048E + 02	2.0514E + 02	4.6762E + 02	2.5130E + 02	2.8902E + 02
	Rank	3	4	1	6	2	7	5
F20	Best	2.5762E + 03	3.2391E + 03	2.7025E + 03	3.0793E + 03	2.6997E + 03	3.1686E + 03	2.9762E + 03
	Mean	2.8963E + 03	3.3706E + 03	2.8129E + 03	3.1656E + 03	2.8090E + 03	3.2227E + 03	3.0253E + 03
	Std	2.4950E + 02	6.9273E + 01	6.1040E + 01	3.8068E + 01	5.4197E + 01	2.6842E + 01	2.4249E + 01
	Rank	3	7	2	5	1	6	4
F21	Best	2.8692E + 04	2.7228E + 04	1.7344E + 04	3.0436E + 04	2.2720E + 04	3.0886E + 04	3.0700E + 04
	Mean	2.9926E + 04	2.8952E + 04	2.0214E + 04	3.1433E + 04	2.6992E + 04	3.2602E + 04	3.2660E + 04
	Std	4.8877E + 02	6.7121E + 02	1.3198E + 03	5.5854E + 02	1.7241E + 03	6.9477E + 02	8.2253E + 02
	Rank	4	3	1	5	2	6	7
F22	Best	3.1244E + 03	3.7857E + 03	3.1517E + 03	3.0063E + 03	3.1791E + 03	3.6939E + 03	2.9614E + 03
	Mean	3.2641E + 03	3.9575E + 03	3.2344E + 03	3.0749E + 03	3.2741E + 03	3.7756E + 03	3.2743E + 03
	Std	8.5084E + 01	6.2468E + 01	4.5834E + 01	8.8989E + 01	5.3062E + 01	3.6845E + 01	2.1926E + 02
	Rank	3	7	2	1	4	6	5
F23	Best	3.4807E + 03	4.2957E + 03	3.6305E + 03	3.4732E + 03	3.6157E + 03	4.0976E + 03	3.3181E + 03
	Mean	3.7464E + 03	4.4823E + 03	3.7557E + 03	3.6628E + 03	3.7223E + 03	4.2238E + 03	3.4285E + 03
	Std	1.4054E + 02	8.6097E + 01	6.3187E + 01	1.7453E + 02	5.8383E + 01	4.7187E + 01	1.5524E + 02
	Rank	4	7	5	2	3	6	1
F24	Best	3.1372E + 03	3.3339E + 03	3.5509E + 03	3.2919E + 03	3.6424E + 03	3.7689E + 03	3.2276E + 03
	Mean	3.2328E + 03	3.4740E + 03	3.7326E + 03	3.4059E + 03	3.8807E + 03	3.9428E + 03	3.3102E + 03
	Std	5.4325E + 01	6.6301E + 01	7.2335E + 01	5.3184E + 01	1.0950E + 02	1.0578E + 02	3.0853E + 01
	Rank	1	4	5	3	6	7	2
F25	Best	8.5685E + 03	1.2147E + 04	9.7962E + 03	7.8622E + 03	9.2636E + 03	1.4046E + 04	5.9295E + 03
	Mean	1.0285E + 04	1.7055E + 04	1.0989E + 04	9.2728E + 03	1.0674E + 04	1.5021E + 04	6.7708E + 03
	Std	1.3210E + 03	1.1837E + 03	5.1435E + 02	1.6464E + 03	8.3845E + 02	4.4791E + 02	9.5689E + 02
	Rank	3	7	5	2	4	6	1
F26	Best	3.3863E + 03	3.6509E + 03	3.4574E + 03	3.3565E + 03	3.4338E + 03	3.6824E + 03	3.3170E + 03
	Mean	3.4910E + 03	3.8614E + 03	3.5513E + 03	3.4446E + 03	3.6595E + 03	3.8161E + 03	3.3481E + 03
	Std	7.8241E + 01	1.3643E + 02	6.6624E + 01	5.2872E + 01	6.7608E + 01	8.1023E + 01	2.0529E + 01
	Rank	3	7	4	2	5	6	1
F27	Best	3.2620E + 03	3.4953E + 03	3.6230E + 03	3.4610E + 03	3.9161E + 03	3.9751E + 03	3.3383E + 03
	Mean	3.3381E + 03	3.6254E + 03	3.7955E + 03	3.5222E + 03	4.1097E + 03	4.3352E + 03	3.4032E + 03
	Std	4.9672E + 01	7.6599E + 01	1.0481E + 02	4.4618E + 01	1.5141E + 02	2.0749E + 02	3.3950E + 01
	Rank	1	4	5	3	6	7	2
F28	Best	4.1747E + 03	5.9941E + 03	5.5900E + 03	4.8547E + 03	5.6151E + 03	8.2019E + 03	6.6339E + 03
	Mean	5.7039E + 03	8.4348E + 03	6.5300E + 03	7.2115E + 03	6.6947E + 03	9.1633E + 03	7.4893E + 03
	Std	7.2025E + 02	1.1455E + 03	4.7024E + 02	1.4517E + 03	5.6588E + 02	3.6830E + 02	4.1121E + 02
	Rank	1	6	2	4	3	7	5
F29	Best	5.8090E + 03	4.1378E + 04	9.0424E + 05	7.8260E + 03	5.1366E + 05	1.0810E + 06	1.0999E + 04
	Mean	4.3415E + 04	1.8021E + 05	2.2380E + 06	1.3900E + 04	1.7061E + 06	2.4739E + 06	1.7091E + 04
	Std	1.6219E + 05	1.3755E + 05	9.9383E + 05	4.5772E + 03	5.7704E + 05	8.0547E + 05	3.5625E + 03
	Rank	3	4	6	1	5	7	2

**Table 22:** The statistics results of MSEDO and improved algorithms solving CEC2022 (D = 10).

No	Index	MSEDO	EDO	IRIME	EMTLBO	EOSMA	MTVSCA	APSM-jSO
F1	Best	3.0000E + 02	8.7876E + 02	3.8362E + 02	3.0162E + 02	4.1176E + 02	3.3211E + 02	3.0006E + 02
	Mean	3.0000E + 02	2.2632E + 03	8.6200E + 02	6.8618E + 02	7.2111E + 02	3.9876E + 02	3.0025E + 02
	Std	4.3522E − 14	8.3485E + 02	3.8814E + 02	7.9209E + 02	1.7118E + 02	6.0430E + 01	1.8424E − 01
	Rank	1	7	6	4	5	3	2
F2	Best	4.0000E + 02	4.0000E + 02	4.0521E + 02	4.0005E + 02	4.0014E + 02	4.0091E + 02	4.0017E + 02
	Mean	4.0485E + 02	4.0503E + 02	4.0829E + 02	4.0502E + 02	4.0442E + 02	4.0559E + 02	4.0623E + 02
	Std	1.2778E + 01	3.3098E + 00	9.7598E − 01	3.7677E + 00	3.9534E + 00	3.2908E + 00	2.5301E + 00
	Rank	2	4	7	3	1	5	6
F3	Best	6.0000E + 02	6.0000E + 02	6.0014E + 02	6.0019E + 02	6.0010E + 02	6.0050E + 02	6.0018E + 02
	Mean	6.0056E + 02	6.0004E + 02	6.0040E + 02	6.0038E + 02	6.0026E + 02	6.0101E + 02	6.0037E + 02
	Std	1.4918E + 00	5.9272E − 02	1.9723E − 01	1.2562E − 01	1.6875E − 01	2.7086E − 01	1.1973E − 01
	Rank	6	1	5	4	2	7	3
F4	Best	8.0298E + 02	8.1318E + 02	8.0717E + 02	8.2090E + 02	8.0684E + 02	8.1302E + 02	8.1661E + 02
	Mean	8.1907E + 02	8.2380E + 02	8.1683E + 02	8.3418E + 02	8.1337E + 02	8.3226E + 02	8.3442E + 02
	Std	1.1159E + 01	5.6819E + 00	5.7137E + 00	5.0003E + 00	4.4926E + 00	5.6860E + 00	6.4262E + 00
	Rank	3	4	2	6	1	5	7
F5	Best	9.0000E + 02	9.0000E + 02	9.0016E + 02	9.0001E + 02	9.0001E + 02	9.0057E + 02	9.0001E + 02
	Mean	9.0035E + 02	9.0009E + 02	9.0081E + 02	9.0004E + 02	9.0029E + 02	9.0153E + 02	9.0007E + 02
	Std	1.1638E + 00	2.0934E − 01	8.4344E − 01	3.7436E − 02	2.9487E − 01	9.5126E − 01	5.3140E − 02
	Rank	5	3	6	1	4	7	2
F6	Best	1.8000E + 03	2.0045E + 03	1.9255E + 03	1.8180E + 03	3.6860E + 03	1.8672E + 03	1.8084E + 03
	Mean	1.8022E + 03	4.6887E + 03	6.5782E + 03	2.7257E + 03	1.1231E + 04	1.9699E + 03	1.8386E + 03
	Std	6.3734E + 00	1.8429E + 03	4.4505E + 03	1.7332E + 03	7.7952E + 03	9.4861E + 01	2.4486E + 01
	Rank	1	5	6	4	7	3	2
F7	Best	2.0000E + 03	2.0144E + 03	2.0037E + 03	2.0265E + 03	2.0079E + 03	2.0177E + 03	2.0271E + 03
	Mean	2.0149E + 03	2.0265E + 03	2.0207E + 03	2.0346E + 03	2.0246E + 03	2.0313E + 03	2.0389E + 03
	Std	2.2938E + 01	3.8551E + 00	4.0595E + 00	4.1402E + 00	6.2622E + 00	4.3953E + 00	4.1737E + 00
	Rank	1	4	2	6	3	5	7
F8	Best	2.2017E + 03	2.2113E + 03	2.2044E + 03	2.2135E + 03	2.2104E + 03	2.2207E + 03	2.2213E + 03
	Mean	2.2337E + 03	2.2228E + 03	2.2195E + 03	2.2265E + 03	2.2256E + 03	2.2257E + 03	2.2273E + 03
	Std	4.2763E + 01	3.3278E + 00	6.0772E + 00	3.9808E + 00	4.1996E + 00	2.1809E + 00	2.6160E + 00
	Rank	7	2	1	5	3	4	6
F9	Best	2.4000E + 03	2.5293E + 03	2.5293E + 03	2.5293E + 03	2.5293E + 03	2.5301E + 03	2.5293E + 03
	Mean	2.5207E + 03	2.5293E + 03	2.5293E + 03	2.5293E + 03	2.5295E + 03	2.5310E + 03	2.5293E + 03
	Std	3.2800E + 01	2.5059E − 02	4.2286E − 03	3.5699E − 03	1.8651E − 01	6.7311E − 01	1.7446E − 03
	Rank	1	5	4	3	6	7	2
F10	Best	2.4070E + 03	2.5003E + 03	2.5003E + 03	2.5003E + 03	2.5003E + 03	2.5004E + 03	2.5003E + 03
	Mean	2.5015E + 03	2.5004E + 03	2.5005E + 03	2.5005E + 03	2.5004E + 03	2.5005E + 03	2.5093E + 03
	Std	2.7193E + 01	5.7553E − 02	8.8604E − 02	7.0876E − 02	8.5714E − 02	8.6580E − 02	3.3611E + 01
	Rank	6	1	4	3	2	5	7
F11	Best	2.6000E + 03	2.6001E + 03	2.6381E + 03	2.6019E + 03	2.6165E + 03	2.7176E + 03	2.6066E + 03
	Mean	2.8487E + 03	2.8701E + 03	2.7215E + 03	2.6397E + 03	2.7554E + 03	2.8904E + 03	2.8700E + 03
	Std	1.1799E + 02	9.1419E + 01	3.6154E + 01	5.9596E + 01	1.2533E + 02	1.1498E + 02	1.0193E + 02
	Rank	4	6	2	1	3	7	5
F12	Best	2.8639E + 03	2.8602E + 03	2.8587E + 03	2.8608E + 03	2.8628E + 03	2.8648E + 03	2.8613E + 03
	Mean	2.8664E + 03	2.8643E + 03	2.8626E + 03	2.8625E + 03	2.8646E + 03	2.8659E + 03	2.8641E + 03
	Std	2.0092E + 00	1.2616E + 00	1.3313E + 00	8.4007E − 01	7.5619E − 01	6.4319E − 01	1.0741E + 00
	Rank	7	4	2	1	5	6	3

**Table 23:** The statistics results of MSEDO and improved algorithms solving CEC2022 (D = 20).

No	Index	MSEDO	EDO	IRIME	EMTLBO	EOSMA	MTVSCA	APSM-jSO
F1	Best	3.0000E + 02	9.6051E + 03	7.1132E + 03	8.5904E + 02	2.9015E + 03	8.8042E + 02	3.0089E + 02
	Mean	3.0000E + 02	2.1724E + 04	1.6314E + 04	4.5925E + 03	7.5927E + 03	2.5056E + 03	3.0758E + 02
	Std	9.0394E − 08	5.6737E + 03	5.1387E + 03	3.0504E + 03	2.4057E + 03	7.7629E + 02	9.6879E + 00
	Rank	1	7	6	4	5	3	2
F2	Best	4.0000E + 02	4.4490E + 02	4.4520E + 02	4.4911E + 02	4.4976E + 02	4.3665E + 02	4.4493E + 02
	Mean	4.3884E + 02	4.5124E + 02	4.5512E + 02	4.5844E + 02	4.6100E + 02	4.5745E + 02	4.4843E + 02
	Std	1.9476E + 01	7.4382E + 00	9.4449E + 00	1.1780E + 01	1.0052E + 01	8.3855E + 00	1.5186E + 00
	Rank	1	3	4	6	7	5	2
F3	Best	6.0000E + 02	6.0010E + 02	6.0069E + 02	6.0056E + 02	6.0059E + 02	6.0165E + 02	6.0021E + 02
	Mean	6.0061E + 02	6.0066E + 02	6.0116E + 02	6.0109E + 02	6.0128E + 02	6.0273E + 02	6.0039E + 02
	Std	1.5761E + 00	7.1989E − 01	3.1829E − 01	3.4333E − 01	4.8219E − 01	7.2885E − 01	9.3051E − 02
	Rank	2	3	5	4	6	7	1
F4	Best	8.2089E + 02	8.5664E + 02	8.2479E + 02	8.9142E + 02	8.2821E + 02	8.8373E + 02	8.8466E + 02
	Mean	8.4812E + 02	8.8172E + 02	8.5326E + 02	9.0952E + 02	8.4596E + 02	9.0459E + 02	9.0185E + 02
	Std	1.8245E + 01	1.2250E + 01	1.4687E + 01	9.3167E + 00	8.7483E + 00	9.3518E + 00	9.2147E + 00
	Rank	2	4	3	7	1	6	5
F5	Best	9.0000E + 02	9.0056E + 02	9.0654E + 02	9.0020E + 02	9.0092E + 02	9.0335E + 02	9.0004E + 02
	Mean	9.4602E + 02	9.2659E + 02	9.3656E + 02	9.0128E + 02	9.0955E + 02	9.1048E + 02	9.0016E + 02
	Std	1.5860E + 02	6.5319E + 01	3.0476E + 01	1.0739E + 00	7.4816E + 00	5.5227E + 00	6.8847E − 02
	Rank	7	5	6	2	3	4	1
F6	Best	1.8002E + 03	3.3424E + 03	3.8557E + 03	1.8460E + 03	5.0444E + 03	5.9699E + 03	1.9976E + 03
	Mean	1.8111E + 03	1.5245E + 04	2.5357E + 04	5.1833E + 03	4.4256E + 04	3.1041E + 04	2.3463E + 03
	Std	2.2581E + 01	1.2646E + 04	2.1089E + 04	3.8252E + 03	6.1985E + 04	1.7529E + 04	2.6416E + 02
	Rank	1	4	5	3	7	6	2
F7	Best	2.0149E + 03	2.0463E + 03	2.0269E + 03	2.0880E + 03	2.0343E + 03	2.0636E + 03	2.0774E + 03
	Mean	2.0907E + 03	2.0753E + 03	2.0388E + 03	2.1087E + 03	2.0544E + 03	2.0855E + 03	2.0964E + 03
	Std	8.7455E + 01	1.4856E + 01	8.6150E + 00	1.1457E + 01	9.1537E + 00	8.2009E + 00	1.1242E + 01
	Rank	5	3	1	7	2	4	6
F8	Best	2.2225E + 03	2.2272E + 03	2.2231E + 03	2.2285E + 03	2.2288E + 03	2.2307E + 03	2.2320E + 03
	Mean	2.2379E + 03	2.2302E + 03	2.2250E + 03	2.2584E + 03	2.2334E + 03	2.2365E + 03	2.2380E + 03
	Std	3.5363E + 01	1.6912E + 00	9.9341E − 01	5.3237E + 01	2.5095E + 00	2.9673E + 00	3.3697E + 00
	Rank	5	2	1	7	3	4	6
F9	Best	2.4808E + 03	2.4808E + 03	2.4808E + 03	2.4809E + 03	2.4811E + 03	2.4825E + 03	2.4808E + 03
	Mean	2.4808E + 03	2.4814E + 03	2.4812E + 03	2.4811E + 03	2.4819E + 03	2.4836E + 03	2.4808E + 03
	Std	6.7826E − 07	6.0194E − 01	3.5524E − 01	2.5370E − 01	5.1555E − 01	6.0588E − 01	5.3016E − 03
	Rank	1	5	4	3	6	7	2
F10	Best	2.5002E + 03	2.5005E + 03	2.5004E + 03	2.5005E + 03	2.5004E + 03	2.5006E + 03	2.5005E + 03
	Mean	2.8539E + 03	3.8384E + 03	2.5056E + 03	2.6403E + 03	2.5007E + 03	2.5123E + 03	2.5008E + 03
	Std	5.8060E + 02	9.9703E + 02	2.7337E + 01	6.8881E + 02	1.2790E − 01	4.2362E + 01	1.4307E − 01
	Rank	6	7	3	5	1	4	2
F11	Best	2.9000E + 03	2.9000E + 03	3.0298E + 03	2.9206E + 03	2.9720E + 03	3.2822E + 03	2.9102E + 03
	Mean	2.9000E + 03	2.9005E + 03	3.2945E + 03	2.9533E + 03	3.0363E + 03	3.5069E + 03	2.9235E + 03
	Std	2.6504E − 11	5.2304E − 01	8.5636E + 01	4.0785E + 01	4.6826E + 01	1.1098E + 02	6.2693E + 00
	Rank	1	2	6	4	5	7	3
F12	Best	2.9351E + 03	2.9421E + 03	2.9362E + 03	2.9376E + 03	2.9394E + 03	2.9691E + 03	2.9330E + 03
	Mean	2.9622E + 03	2.9541E + 03	2.9443E + 03	2.9458E + 03	2.9523E + 03	2.9832E + 03	2.9417E + 03
	Std	3.0725E + 01	1.0488E + 01	4.5961E + 00	5.5092E + 00	6.8248E + 00	1.0156E + 01	5.6545E + 00
	Rank	6	5	2	3	4	7	1

**Table 24:** The statistics results of MSEDO and competitors solving constrained engineering optimization.

**No**	Index	MSEDO	EDO	RIME	AE	MRFO	APSM − Jso	IRIME	EMTLBO
CE1	Best	1.2668E − 02	1.2692E − 02	1.3181E − 02	1.3552E − 02	1.2780E − 02	1.3310E − 02	1.2846E − 02	1.2908E − 02
	Mean	1.2681E − 02	2.8988E + 04	1.8121E − 02	1.6280E − 02	1.3870E − 02	1.6915E − 02	1.5274E − 02	1.4412E − 02
	Std	1.4231E − 05	1.5800E + 05	2.2194E − 03	1.5071E − 03	7.7632E − 04	2.2220E − 03	2.7399E − 03	1.3593E − 03
	Rank	1	8	7	5	2	6	4	3
CE2	Best	5.8701E + 03	6.2437E + 03	5.9598E + 03	7.6021E + 03	6.3297E + 03	7.9456E + 03	6.7770E + 03	6.6407E + 03
	Mean	5.8876E + 03	7.7030E + 03	7.3437E + 03	1.0786E + 04	7.0627E + 03	1.2331E + 04	1.0594E + 04	9.6043E + 03
	Std	4.2611E + 01	1.9944E + 03	2.1207E + 03	2.1038E + 03	5.3569E + 02	3.1795E + 03	2.1162E + 03	1.7634E + 03
	Rank	1	4	3	7	2	8	6	5
CE3	Best	2.6389E + 02	2.6389E + 02	2.6389E + 02	2.6389E + 02	2.6389E + 02	2.6389E + 02	2.6396E + 02	2.6389E + 02
	Mean	2.6389E + 02	Inf	2.6463E + 02	2.6422E + 02	2.6399E + 02	2.6453E + 02	2.6491E + 02	2.6432E + 02
	Std	5.5335E − 05	NaN	8.6336E − 01	3.0395E − 01	1.1155E − 01	5.1059E − 01	7.4696E − 01	4.7128E − 01
	Rank	1	8	6	3	2	5	7	4
CE4	Best	1.6928E + 00	1.7344E + 00	1.7197E + 00	1.8679E + 00	1.7423E + 00	1.7869E + 00	1.7935E + 00	1.7496E + 00
	Mean	1.6930E + 00	1.8453E + 00	1.9938E + 00	2.0230E + 00	1.8367E + 00	1.9441E + 00	2.1388E + 00	2.0961E + 00
	Std	1.0667E − 04	1.2522E − 01	2.4970E − 01	1.2275E − 01	7.2188E − 02	7.2575E − 02	2.0527E − 01	5.2775E − 01
	Rank	1	3	5	6	2	4	8	7
CE5	Best	2.9936E + 03	2.9962E + 03	2.9966E + 03	2.7953E + 03	3.0046E + 03	3.0275E + 03	3.0034E + 03	2.9950E + 03
	Mean	2.9936E + 03	3.0030E + 03	3.0091E + 03	3.0307E + 03	3.0149E + 03	3.0464E + 03	3.0150E + 03	3.0030E + 03
	Std	6.8770E − 03	3.9793E + 00	1.3163E + 01	5.9620E + 01	6.5711E + 00	1.8564E + 01	9.4815E + 00	4.4280E + 00
	Rank	1	2	4	7	5	8	6	3
CE6	Best	2.7009E − 12	9.9216E − 10	2.3078E − 11	2.3078E − 11	2.7009E − 12	2.7009E − 12	2.3078E − 11	2.7009E − 12
	Mean	2.3828E − 09	2.4409E − 02	4.5599E − 09	2.7860E − 09	9.5034E − 10	4.5379E − 09	3.8902E − 08	5.6213E − 09
	Std	5.7109E − 09	1.3369E-01	6.6495E − 09	3.2762E − 09	9.0318E − 10	7.2891E − 09	1.6199E − 07	8.7759E − 09
	Rank	2	8	5	3	1	4	7	6
CE7	Best	− 2.4358E + 05	− 2.4358E + 05	− 2.4358E + 05	− 2.0665E + 05	− 2.4145E + 05	− 2.4295E + 05	− 2.4358E + 05	− 2.4358E + 05
	Mean	− 2.4358E + 05	− 2.4353E + 05	− 2.4227E + 05	− 3.3497E + 04	− 2.3715E + 05	− 2.3792E + 05	− 2.4343E + 05	− 2.4358E + 05
	Std	1.8065E − 01	8.7865E + 01	2.7198E + 03	6.5500E + 04	3.7512E + 03	3.4820E + 03	3.9033E + 02	7.6725E − 01
	Rank	1	3	5	8	7	6	4	2
CE8	Best	1.3400E + 00	1.3502E + 00	1.3455E + 00	1.4007E + 00	1.3410E + 00	1.4286E + 00	1.3712E + 00	1.3777E + 00
	Mean	1.3400E + 00	5.2083E + 20	1.4055E + 00	1.5947E + 00	1.3505E + 00	1.6081E + 00	1.7281E + 00	1.6974E + 00
	Std	6.6771E − 08	2.8527E + 21	4.6013E − 02	9.8418E − 02	6.1893E − 03	1.2712E − 01	1.8028E − 01	2.3529E − 01
	Rank	1	8	3	4	2	5	7	6.0000E + 00
CE9	Best	3.9247E + 08	3.9247E + 08	3.9247E + 08	2.5187E + 08	3.9247E + 08	3.9247E + 08	3.9247E + 08	3.9247E + 08
	Mean	3.9247E + 08	3.9247E + 08	3.9247E + 08	2.5328E + 08	3.9247E + 08	3.9247E + 08	3.9247E + 08	3.9247E + 08
	Std	1.8187E − 07	1.8187E − 07	1.8187E − 07	3.7425E + 06	5.3206E + 02	6.9831E + 03	1.8187E − 07	1.8187E − 07
	Rank	2	2	2	1	7	8	2	2
CE10	Best	1.6086E + 01	1.6549E + 01	1.6333E + 01	2.0652E + 01	1.6717E + 01	1.7178E + 01	1.7220E + 01	1.6732E + 01
	Mean	1.6086E + 01	8.3410E + 10	1.7258E + 01	1.4850E + 02	1.8393E + 01	2.9160E + 01	2.2468E + 01	3.2971E + 01
	Std	8.4814E − 06	3.1743E + 11	5.1070E − 01	1.0528E + 02	2.0205E + 00	1.3542E + 01	6.6557E + 00	6.9061E + 01
	Rank	1	8	2	7	3	5	4	6
