# Supplementary material for: Multi-strategy enterprise development optimizer for numerical optimization and constrained problems
Source: Sci Rep. 2025 Mar 27;15:10538. doi: 10.1038/s41598-025-93754-3 (PMC11950178; doi:10.1038/s41598-025-93754-3)
Supplement: Supplementary file 1 — Supplementary Information. [file 41598_2025_93754_MOESM1_ESM.pdf]

SUPPLEMENTARY FILE FOR PAPER

Paper title:

Multi-Strategy Enterprise Development Optimizer for Numerical Optimization and Constrained Problems

Author:

Xinyu Cai [1], Weibin Wang [1, *] and Yijiang Wang [2]

Affiliation:

[1] College of Business, Jiaxing University; Jiaxing 314001, China.

[2] School of Labor and Human Resources, Renmin University of China, Beijing 100872, China

Tables:

Table S1. CEC 2017 test functions

Type	ID	CEC 2017 Function name	Search range	fmin
Unimodal	F1	Shifted and Rotated Bent Cigar Function	[-100,100]	100
	F2	Shifted and Rotated Zakharov Function	[-100,100]	300
Multimodal	F3	Shifted and Rotated Rosenbrock's Function	[-100,100]	400
	F4	Shifted and Rotated Rastrigin's Function	[-100,100]	500
	F5	Shifted and Rotated Expanded Scaffer's F6 Function	[-100,100]	600
	F6	Shifted and Rotated Lunacek Bi_Rastrigin Function	[-100,100]	700
	F7	Shifted and Rotated Non-Continuous Rastrigin's Function	[-100,100]	800
	F8	Shifted and Rotated Levy Function	[-100,100]	900
	F9	Shifted and Rotated Schwefel's Function	[-100,100]	1000
Hybrid	F10	Hybrid Function 1 (N=3)	[-100,100]	1100
	F11	Hybrid Function 2 (N=3)	[-100,100]	1200
	F12	Hybrid Function 3 (N=3)	[-100,100]	1300
	F13	Hybrid Function 4 (N=4)	[-100,100]	1400
	F14	Hybrid Function 5 (N=4)	[-100,100]	1500
	F15	Hybrid Function 6 (N=4)	[-100,100]	1600
	F16	Hybrid Function 6 (N=5)	[-100,100]	1700
	F17	Hybrid Function 6 (N=5)	[-100,100]	1800
	F18	Hybrid Function 6 (N=5)	[-100,100]	1900
	F19	Hybrid Function 6 (N=6)	[-100,100]	2000
Composition	F20	Composition Function 1 (N=3)	[-100,100]	2100
	F21	Composition Function 2 (N=3)	[-100,100]	2200
	F22	Composition Function 3 (N=4)	[-100,100]	2300
	F23	Composition Function 4 (N=4)	[-100,100]	2400

F24	Composition Function 5 (N=5)	[-100,100]	2500
F25	Composition Function 6 (N=5)	[-100,100]	2600
F26	Composition Function 7 (N=6)	[-100,100]	2700
F27	Composition Function 8 (N=6)	[-100,100]	2800
F28	Composition Function 9 (N=3)	[-100,100]	2900
F29	Composition Function 10 (N=3)	[-100,100]	3000

Table S2. CEC 2022 test functions

Type	ID	CEC 2022 Function name	Search range	f_{min}
Unimodal functions	F1	Shifted and full Rotated Zakharov Function	[-100,100]	300
Multimodal functions	F2	Shifted and full Rotated Rosenbrock's Function	[-100,100]	400
	F3	Shifted and full Rotated Expanded Schaffer's f6 Function	[-100,100]	600
	F4	Shifted and full Rotated Non-Continuous Rastrigin's Function	[-100,100]	800
	F5	Shifted and full Rotated Levy Function	[-100,100]	900
Hybrid functions	F6	Hybrid Function 1 (N = 3)	[-100,100]	1800
	F7	Hybrid Function 2 (N = 6	[-100,100]	2000
	F8	Hybrid Function 3 (N = 5)	[-100,100]	2200
Composition functions	F9	Composition Function 1 (N = 5)	[-100,100]	2300
	F10	Composition Function 2 (N = 4)	[-100,100]	2400
	F11	Composition Function 3 (N = 5)	[-100,100]	2600
	F12	Composition Function 4 (N = 6)	[-100,100]	2700

Table S3. The statistics results of MSEDO and basic algorithms solving CEC2017 (D=10)

No.	Index	MSEDO	EDO	RIME	ECO	MRFO	QIO	AE
F1	Best	1.0001E+02	1.0014E+02	7.1759E+03	1.0146E+02	1.0152E+02	2.1725E+03	1.0175E+02
	Mean	1.0025E+02	3.5109E+03	3.5879E+04	3.7647E+03	1.2944E+03	2.0785E+04	3.5687E+02
	Std	1.2520E+00	2.7405E+03	2.1751E+04	3.8188E+03	1.3843E+03	2.1482E+04	3.5630E+02
	Rank	1	4	7	5	3	6	2
F2	Best	3.0000E+02	1.0202E+03	3.0027E+02	3.0000E+02	3.0002E+02	3.3928E+02	3.0229E+02
	Mean	3.0000E+02	3.1180E+03	3.0140E+02	3.0014E+02	3.0028E+02	5.5043E+02	3.1035E+02
	Std	9.9581E-14	1.5322E+03	7.9329E-01	1.9823E-01	3.7541E-01	1.6905E+02	7.1284E+00
	Rank	1	7	4	2	3	6	5
F3	Best	4.0000E+02	4.0243E+02	4.0011E+02	4.0003E+02	4.0001E+02	4.0033E+02	4.0422E+02
	Mean	4.0000E+02	4.0477E+02	4.0786E+02	4.0680E+02	4.0594E+02	4.0488E+02	4.0467E+02
	Std	8.6802E-10	7.3177E-01	1.2133E+01	1.2953E+01	1.2775E+01	2.1774E+00	2.1146E-01
	Rank	1	3	7	6	5	4	2
F4	Best	5.0398E+02	5.1360E+02	5.0401E+02	5.0913E+02	5.0398E+02	5.0225E+02	5.0092E+02
	Mean	5.2172E+02	5.2351E+02	5.1355E+02	5.2323E+02	5.2008E+02	5.1777E+02	5.2110E+02
	Std	2.3103E+01	4.9301E+00	6.2207E+00	1.0744E+01	9.4699E+00	6.8669E+00	9.1780E+00
	Rank	5	7	1	6	3	2	4
F5	Best	6.0000E+02	6.0000E+02	6.0007E+02	6.0017E+02	6.0000E+02	6.0023E+02	6.0000E+02
	Mean	6.0026E+02	6.0010E+02	6.0022E+02	6.0744E+02	6.0005E+02	6.0060E+02	6.0000E+02
	Std	1.0890E+00	3.0012E-01	9.0413E-02	5.9070E+00	2.1475E-01	3.4784E-01	9.8908E-04
	Rank	5	3	4	7	2	6	1
F6	Best	7.0533E+02	7.2212E+02	7.0898E+02	7.2037E+02	7.1462E+02	7.2096E+02	7.1953E+02
	Mean	7.2163E+02	7.3284E+02	7.2278E+02	7.4163E+02	7.3282E+02	7.4014E+02	7.3530E+02
	Std	9.1725E+00	6.1432E+00	6.5389E+00	1.6724E+01	8.2380E+00	6.8364E+00	5.7373E+00
	Rank	1	4	2	7	3	6	5
F7	Best	8.0398E+02	8.1182E+02	8.0799E+02	8.0598E+02	8.0597E+02	8.0298E+02	8.0074E+02
	Mean	8.2106E+02	8.2262E+02	8.1379E+02	8.2220E+02	8.1724E+02	8.1272E+02	8.1836E+02

	Std	1.1172E+01	4.6598E+00	4.1310E+00	7.1955E+00	7.0681E+00	5.2485E+00	9.7398E+00
	Rank	5	7	2	6	3	1	4
F8	Best	9.0000E+02	9.0000E+02	9.0001E+02	9.0000E+02	9.0000E+02	9.0002E+02	9.0000E+02
	Mean	9.0009E+02	9.0002E+02	9.0025E+02	9.2885E+02	9.0006E+02	9.0019E+02	9.0000E+02
	Std	1.9151E-01	8.4851E-02	2.9480E-01	3.8848E+01	1.5084E-01	1.8035E-01	3.5628E-06
	Rank	4	2	6	7	3	5	1
F9	Best	1.1231E+03	1.9125E+03	1.1303E+03	1.2546E+03	1.2404E+03	1.4893E+03	1.9099E+03
	Mean	1.7766E+03	2.0972E+03	1.4579E+03	1.7255E+03	1.6927E+03	2.3299E+03	2.3707E+03
	Std	3.3407E+02	1.0050E+02	2.5502E+02	2.9233E+02	2.6921E+02	3.0748E+02	1.8050E+02
	Rank	4	5	1	3	2	6	7
F10	Best	1.1000E+03	1.1017E+03	1.1029E+03	1.1078E+03	1.1014E+03	1.1052E+03	1.1042E+03
	Mean	1.1058E+03	1.1064E+03	1.1123E+03	1.1362E+03	1.1081E+03	1.1102E+03	1.1074E+03
	Std	4.9329E+00	2.9064E+00	6.0384E+00	4.5102E+01	4.5870E+00	3.7743E+00	2.0807E+00
	Rank	1	2	6	7	4	5	3
F11	Best	1.2000E+03	2.1599E+04	1.0347E+04	2.2292E+03	2.8316E+03	7.4045E+03	6.4496E+03
	Mean	1.2664E+03	3.0129E+05	1.8735E+05	2.1422E+04	1.4341E+04	3.9582E+04	1.6894E+04
	Std	9.3930E+01	3.6239E+05	3.5720E+05	1.8320E+04	1.0465E+04	3.5059E+04	4.7285E+03
	Rank	1	7	6	4	2	5	3
F12	Best	1.3003E+03	1.3689E+03	1.3418E+03	1.5918E+03	1.3540E+03	1.3161E+03	1.3792E+03
	Mean	1.3039E+03	5.0469E+03	8.7790E+03	2.1893E+03	2.8525E+03	1.3353E+03	1.5788E+03
	Std	3.3659E+00	5.6742E+03	7.6455E+03	5.9496E+02	1.7069E+03	1.2175E+01	9.1140E+01
	Rank	1	6	7	4	5	2	3
F13	Best	1.4000E+03	1.4458E+03	1.4076E+03	1.4271E+03	1.4410E+03	1.4117E+03	1.4288E+03
	Mean	1.4031E+03	1.5549E+03	2.1627E+03	1.4798E+03	1.4927E+03	1.4227E+03	1.4411E+03
	Std	5.7833E+00	2.2567E+02	1.6563E+03	3.0417E+01	2.8265E+01	5.0204E+00	5.4244E+00
	Rank	1	6	7	4	5	2	3
F14	Best	1.5000E+03	1.5358E+03	1.5076E+03	1.5191E+03	1.5494E+03	1.5030E+03	1.5221E+03
	Mean	1.5014E+03	1.7364E+03	2.8904E+03	1.6340E+03	1.8289E+03	1.5081E+03	1.5414E+03
	Std	5.7709E+00	2.3632E+02	2.5843E+03	7.4486E+01	4.0742E+02	2.7037E+00	1.0479E+01
	Rank	1	5	7	4	6	2	3
F15	Best	1.6000E+03	1.6036E+03	1.6017E+03	1.6023E+03	1.6012E+03	1.6022E+03	1.6078E+03
	Mean	1.7623E+03	1.6352E+03	1.6646E+03	1.6849E+03	1.6837E+03	1.6498E+03	1.6423E+03
	Std	1.0048E+02	3.4732E+01	8.5690E+01	6.1668E+01	1.0147E+02	6.1285E+01	1.9373E+01
	Rank	7	1	4	6	5	3	2
F16	Best	1.7024E+03	1.7171E+03	1.7034E+03	1.7221E+03	1.7021E+03	1.7282E+03	1.7470E+03
	Mean	1.7208E+03	1.7369E+03	1.7448E+03	1.7545E+03	1.7332E+03	1.7505E+03	1.7693E+03
	Std	2.7687E+01	1.0117E+01	4.2459E+01	1.5166E+01	1.7072E+01	1.4698E+01	1.0719E+01
	Rank	1	3	4	6	2	5	7
F17	Best	1.8000E+03	4.1404E+03	1.8504E+03	1.8942E+03	2.0448E+03	1.8233E+03	1.9154E+03
	Mean	1.8032E+03	8.6641E+03	1.4029E+04	4.4660E+03	6.7194E+03	1.8335E+03	2.1203E+03
	Std	8.4084E+00	4.6488E+03	9.3918E+03	4.8197E+03	4.5662E+03	9.6080E+00	1.2341E+02
	Rank	1	6	7	4	5	2	3
F18	Best	1.9000E+03	1.9208E+03	1.9063E+03	1.9099E+03	1.9437E+03	1.9038E+03	1.9072E+03
	Mean	1.9015E+03	1.9758E+03	2.5450E+03	1.9552E+03	2.1147E+03	1.9056E+03	1.9166E+03
	Std	6.7051E+00	6.8381E+01	1.1042E+03	3.8374E+01	2.4171E+02	9.3842E-01	5.3919E+00
	Rank	1	5	7	4	6	2	3
F19	Best	2.0003E+03	2.0243E+03	2.0004E+03	2.0231E+03	2.0013E+03	2.0188E+03	2.0342E+03
	Mean	2.0478E+03	2.0436E+03	2.0170E+03	2.0676E+03	2.0214E+03	2.0422E+03	2.0535E+03
	Std	6.2270E+01	9.9469E+00	1.0933E+01	3.1634E+01	1.2639E+01	1.4614E+01	1.0846E+01

	Rank	5	4	1	7	2	3	6
F20	Best	2.1000E+03	2.2026E+03	2.1001E+03	2.2002E+03	2.2000E+03	2.2004E+03	2.2015E+03
	Mean	2.2855E+03	2.2457E+03	2.2452E+03	2.2243E+03	2.2294E+03	2.2106E+03	2.2759E+03
	Std	5.9326E+01	5.5662E+01	6.4433E+01	4.8349E+01	5.1012E+01	3.0392E+01	5.7710E+01
	Rank	7	5	4	2	3	1	6
F21	Best	2.3003E+03	2.2197E+03	2.2002E+03	2.2000E+03	2.2328E+03	2.2003E+03	2.3000E+03
	Mean	2.3919E+03	2.3221E+03	2.2923E+03	2.2810E+03	2.2982E+03	2.2951E+03	2.3000E+03
	Std	2.9812E+02	1.4412E+02	2.9375E+01	3.5131E+01	1.6446E+01	2.9251E+01	1.6330E-02
	Rank	7	6	2	1	4	3	5
F22	Best	2.6055E+03	2.6104E+03	2.6057E+03	2.6088E+03	2.6060E+03	2.6030E+03	2.6000E+03
	Mean	2.6162E+03	2.6208E+03	2.6146E+03	2.6228E+03	2.6183E+03	2.6152E+03	2.6139E+03
	Std	6.3927E+00	6.0258E+00	5.2787E+00	1.0209E+01	9.9894E+00	9.1539E+00	1.0328E+01
	Rank	4	6	2	7	5	3	1
F23	Best	2.5000E+03	2.5092E+03	2.5002E+03	2.5002E+03	2.5000E+03	2.5007E+03	2.5653E+03
	Mean	2.7365E+03	2.7101E+03	2.6865E+03	2.7267E+03	2.7267E+03	2.6656E+03	2.7346E+03
	Std	4.5287E+01	8.7104E+01	1.0789E+02	7.7404E+01	6.4044E+01	1.0963E+02	3.3652E+01
	Rank	7	3	2	5	4	1	6
F24	Best	2.6000E+03	2.6367E+03	2.8978E+03	2.6005E+03	2.8977E+03	2.8978E+03	2.8983E+03
	Mean	2.9062E+03	2.9050E+03	2.9353E+03	2.9175E+03	2.9138E+03	2.9177E+03	2.9374E+03
	Std	6.2537E+01	5.5194E+01	2.0500E+01	6.4410E+01	2.2419E+01	2.2684E+01	1.7725E+01
	Rank	2	1	6	4	3	5	7
F25	Best	2.8000E+03	2.6019E+03	2.8058E+03	2.8009E+03	2.8000E+03	2.8026E+03	2.9000E+03
	Mean	3.3659E+03	3.0345E+03	2.9064E+03	2.9565E+03	2.8984E+03	2.9020E+03	2.9000E+03
	Std	5.4672E+02	3.8362E+02	4.0183E+01	7.0871E+01	4.5126E+01	2.1759E+01	6.0437E-03
	Rank	7	6	4	5	1	3	2
F26	Best	3.0889E+03	3.0891E+03	3.0893E+03	3.0893E+03	3.0896E+03	3.0905E+03	3.0895E+03
	Mean	3.1003E+03	3.0943E+03	3.0957E+03	3.0939E+03	3.1036E+03	3.0983E+03	3.0913E+03
	Std	1.7097E+01	2.8280E+00	9.4598E+00	3.4982E+00	1.8246E+01	3.0988E+00	2.2581E+00
	Rank	6	3	4	2	7	5	1
F27	Best	3.1000E+03	3.1000E+03	3.1004E+03	3.1001E+03	3.1000E+03	3.1007E+03	3.1000E+03
	Mean	3.2052E+03	3.1300E+03	3.1978E+03	3.2890E+03	3.2235E+03	3.1558E+03	3.1070E+03
	Std	1.3135E+02	3.3936E+01	9.3661E+01	1.4280E+02	1.4344E+02	8.2713E+01	1.6675E+01
	Rank	5	2	4	7	6	3	1
F28	Best	3.1293E+03	3.1543E+03	3.1402E+03	3.1445E+03	3.1473E+03	3.1584E+03	3.1702E+03
	Mean	3.1842E+03	3.1863E+03	3.1778E+03	3.2082E+03	3.1916E+03	3.1996E+03	3.1986E+03
	Std	6.6041E+01	1.6812E+01	2.8551E+01	4.3985E+01	2.6607E+01	2.0587E+01	1.4668E+01
	Rank	2	3	1	7	4	6	5
F29	Best	3.3945E+03	1.3839E+04	3.7232E+03	3.5547E+03	4.3751E+03	5.0412E+03	5.8828E+03
	Mean	3.0644E+04	5.7709E+04	5.6301E+04	1.8563E+05	3.8093E+05	2.0385E+05	5.5625E+04
	Std	1.4920E+05	4.5589E+04	1.4916E+05	5.0809E+05	6.1755E+05	4.0759E+05	7.1338E+04
	Rank	1	4	3	5	7	6	2

Table S4. The statistics results of MSEDO and basic algorithms solving CEC2017 (D=30)

No.	Index	MSEDO	EDO	RIME	ECO	MRFO	QIO	AE
F1	Best	1.0003E+02	3.0527E+02	4.9665E+05	8.1707E+04	9.0817E+02	4.1892E+06	5.7916E+02
	Mean	1.0148E+02	3.0404E+03	1.6954E+06	4.4234E+05	6.3096E+03	8.4807E+06	1.9964E+03
	Std	4.1493E-01	3.6727E+03	9.8409E+05	3.3145E+05	4.6497E+03	3.0371E+06	6.5929E+02
	Rank	1	3	6	5	4	7	2
F2	Best	3.0000E+02	6.0706E+04	2.0915E+03	2.2586E+03	4.6619E+03	3.3664E+04	3.5574E+03

	Mean	3.0000E+02	8.5772E+04	4.7443E+03	8.1312E+03	8.5537E+03	5.2848E+04	8.1368E+03
	Std	7.2411E-07	1.3340E+04	1.8712E+03	4.0344E+03	2.7772E+03	7.6591E+03	2.6268E+03
	Rank	1	7	2	3	5	6	4
F3	Best	4.0000E+02	4.0424E+02	4.7408E+02	4.6482E+02	4.0661E+02	4.7077E+02	4.8630E+02
	Mean	4.2235E+02	4.9474E+02	5.0510E+02	5.0628E+02	5.0342E+02	5.3218E+02	5.1113E+02
	Std	2.9431E+01	3.1250E+01	2.0318E+01	1.7569E+01	2.7837E+01	2.5368E+01	1.0680E+01
	Rank	1	2	4	5	3	7	6
F4	Best	5.4079E+02	6.1971E+02	5.4528E+02	5.7371E+02	5.7761E+02	5.3945E+02	5.1309E+02
	Mean	5.8872E+02	6.7574E+02	5.7123E+02	6.6124E+02	6.3920E+02	6.0573E+02	6.2968E+02
	Std	3.2737E+01	2.0990E+01	1.3785E+01	4.5002E+01	3.9586E+01	3.2983E+01	5.9231E+01
	Rank	2	7	1	6	5	3	4
F5	Best	6.0000E+02	6.0068E+02	6.0070E+02	6.2182E+02	6.0041E+02	6.0287E+02	6.0002E+02
	Mean	6.0197E+02	6.0480E+02	6.0242E+02	6.3792E+02	6.0776E+02	6.0593E+02	6.0011E+02
	Std	2.5760E+00	7.2877E+00	8.7951E-01	9.4947E+00	9.2432E+00	3.5449E+00	7.1555E-02
	Rank	2	4	3	7	6	5	1
F6	Best	7.8002E+02	8.7720E+02	7.7744E+02	8.4215E+02	8.2349E+02	8.5221E+02	7.3755E+02
	Mean	8.4944E+02	9.1222E+02	8.1476E+02	9.8149E+02	9.0730E+02	9.2740E+02	8.7622E+02
	Std	3.3425E+01	1.9114E+01	1.9271E+01	7.7454E+01	6.3124E+01	2.9356E+01	3.8522E+01
	Rank	2	5	1	7	4	6	3
F7	Best	8.4776E+02	9.2645E+02	8.3475E+02	8.8565E+02	8.7562E+02	8.5108E+02	8.0954E+02
	Mean	8.9299E+02	9.7066E+02	8.6629E+02	9.3538E+02	9.2263E+02	8.8331E+02	9.2462E+02
	Std	2.9080E+01	2.2237E+01	1.6614E+01	2.9142E+01	2.5885E+01	2.6946E+01	5.6326E+01
	Rank	3	7	1	6	4	2	5
F8	Best	9.0000E+02	9.3694E+02	9.1849E+02	1.9594E+03	1.3416E+03	9.3047E+02	9.0000E+02
	Mean	1.0213E+03	1.7221E+03	1.0526E+03	3.7405E+03	3.2109E+03	1.0173E+03	9.0017E+02
	Std	4.5338E+02	7.2002E+02	1.2965E+02	1.0700E+03	1.0937E+03	1.1029E+02	2.5125E-01
	Rank	3	5	4	7	6	2	1
F9	Best	3.0658E+03	5.8261E+03	3.4299E+03	3.5898E+03	3.2537E+03	7.6177E+03	7.5316E+03
	Mean	6.6245E+03	6.4926E+03	4.2437E+03	5.3387E+03	4.6202E+03	8.4038E+03	8.2587E+03
	Std	1.5034E+03	2.9803E+02	4.9563E+02	8.1536E+02	6.5728E+02	3.2512E+02	2.7881E+02
	Rank	5	4	1	3	2	7	6
F10	Best	1.1050E+03	1.2366E+03	1.1989E+03	1.1724E+03	1.1450E+03	1.1699E+03	1.1586E+03
	Mean	1.1324E+03	1.3140E+03	1.2801E+03	1.2458E+03	1.2073E+03	1.2729E+03	1.1948E+03
	Std	2.5799E+01	5.4545E+01	4.2880E+01	4.7336E+01	4.0701E+01	3.6874E+01	3.1249E+01
	Rank	1	7	6	4	3	5	2
F11	Best	1.2134E+03	5.9990E+04	1.7053E+06	2.9667E+05	8.3785E+04	2.2602E+05	9.9675E+04
	Mean	5.9259E+03	4.3781E+05	1.0968E+07	1.5561E+06	7.2075E+05	2.6155E+06	2.6421E+05
	Std	8.2464E+03	3.6357E+05	9.0111E+06	9.7778E+05	6.4968E+05	1.7200E+06	1.4006E+05
	Rank	1	3	7	5	4	6	2
F12	Best	1.3445E+03	1.5918E+03	4.0666E+04	4.7579E+03	1.4479E+03	3.5211E+03	3.2353E+03
	Mean	1.1104E+04	3.6597E+04	1.1828E+05	2.5774E+04	1.0107E+04	8.7020E+03	8.2747E+03
	Std	1.9693E+04	1.8302E+04	8.1327E+04	2.3129E+04	1.2793E+04	5.3190E+03	2.8114E+03
	Rank	4	6	7	5	3	2	1
F13	Best	1.4363E+03	7.3693E+03	2.9189E+03	1.6024E+03	3.2584E+03	1.9589E+03	1.6105E+03
	Mean	1.4503E+03	2.9967E+04	3.2661E+04	2.1432E+03	9.4956E+03	1.0472E+04	1.6918E+03
	Std	8.7641E+00	2.3039E+04	2.5367E+04	1.0825E+03	6.7868E+03	8.0811E+03	5.2765E+01
	Rank	1	6	7	3	4	5	2
F14	Best	1.5165E+03	1.5789E+03	5.2173E+03	2.0751E+03	1.5880E+03	1.8023E+03	1.7508E+03
	Mean	2.8983E+03	1.7367E+04	3.5193E+04	1.5639E+04	8.4558E+03	6.5005E+03	2.6741E+03

	Std	5.5076E+03	1.0025E+04	1.6569E+04	1.1669E+04	8.6697E+03	5.3347E+03	7.9073E+02
	Rank	2	6	7	5	4	3	1
F15	Best	1.8149E+03	2.6610E+03	1.8440E+03	2.0966E+03	1.8550E+03	1.9971E+03	2.8130E+03
	Mean	2.3016E+03	3.1266E+03	2.3816E+03	2.7909E+03	2.4499E+03	2.5874E+03	3.0482E+03
	Std	3.3898E+02	1.8984E+02	2.1073E+02	2.6120E+02	3.0025E+02	4.0725E+02	1.3749E+02
	Rank	1	7	2	5	3	4	6
F16	Best	1.7517E+03	1.8166E+03	1.7600E+03	1.8346E+03	1.7947E+03	1.7708E+03	1.9591E+03
	Mean	2.0114E+03	2.1527E+03	1.9380E+03	2.1920E+03	2.0641E+03	1.9101E+03	2.1233E+03
	Std	1.6424E+02	1.7370E+02	1.3525E+02	2.5094E+02	1.4351E+02	1.0259E+02	1.0539E+02
	Rank	3	6	2	7	4	1	5
F17	Best	1.8264E+03	1.2573E+05	9.6483E+04	1.5786E+04	2.6692E+04	1.2233E+05	2.0912E+04
	Mean	1.8318E+03	5.7885E+05	5.1071E+05	7.2081E+04	2.1363E+05	3.3394E+05	3.5593E+04
	Std	1.4891E+01	4.2539E+05	4.0557E+05	6.0600E+04	2.1356E+05	1.7963E+05	9.5223E+03
	Rank	1	7	6	3	4	5	2
F18	Best	1.9173E+03	1.9786E+03	3.1953E+03	2.1611E+03	1.9990E+03	2.0601E+03	2.1474E+03
	Mean	2.6267E+03	2.0098E+04	3.6880E+04	1.0007E+04	1.3049E+04	7.5037E+03	5.3387E+03
	Std	2.5069E+03	1.6027E+04	4.1988E+04	1.1430E+04	1.3109E+04	6.2450E+03	2.3411E+03
	Rank	1	6	7	4	5	3	2
F19	Best	2.0526E+03	2.4584E+03	2.0997E+03	2.2386E+03	2.1602E+03	2.1302E+03	2.4319E+03
	Mean	2.4441E+03	2.7232E+03	2.3739E+03	2.4451E+03	2.3578E+03	2.3664E+03	2.5863E+03
	Std	2.5198E+02	1.1965E+02	1.5669E+02	1.4128E+02	1.4651E+02	1.5147E+02	9.3510E+01
	Rank	4	7	3	5	1	2	6
F20	Best	2.3397E+03	2.4326E+03	2.3458E+03	2.3810E+03	2.2089E+03	2.3399E+03	2.3082E+03
	Mean	2.3797E+03	2.4700E+03	2.3784E+03	2.4444E+03	2.3823E+03	2.3965E+03	2.4155E+03
	Std	3.1585E+01	2.2692E+01	2.0224E+01	4.1382E+01	4.1824E+01	3.4369E+01	6.0758E+01
	Rank	2	7	1	6	3	4	5
F21	Best	2.3000E+03	7.1523E+03	2.3065E+03	2.3058E+03	2.3001E+03	2.3172E+03	2.3000E+03
	Mean	5.5608E+03	7.7073E+03	3.2820E+03	3.6549E+03	2.3008E+03	2.3211E+03	2.3002E+03
	Std	2.7843E+03	3.0644E+02	1.6763E+03	2.2776E+03	1.0476E+00	2.9761E+00	6.1332E-01
	Rank	6	7	4	5	2	3	1
F22	Best	2.6921E+03	2.7734E+03	2.6859E+03	2.7661E+03	2.6974E+03	2.6975E+03	2.6530E+03
	Mean	2.7318E+03	2.8320E+03	2.7256E+03	2.8395E+03	2.7598E+03	2.7703E+03	2.7178E+03
	Std	3.0304E+01	2.5066E+01	1.8817E+01	4.8940E+01	3.0671E+01	3.1670E+01	6.2891E+01
	Rank	3	6	2	7	4	5	1
F23	Best	2.8464E+03	2.9838E+03	2.8541E+03	2.9018E+03	2.8898E+03	2.8612E+03	2.8258E+03
	Mean	2.9011E+03	3.0101E+03	2.9025E+03	2.9771E+03	2.9434E+03	2.9198E+03	2.9281E+03
	Std	3.6066E+01	1.8030E+01	2.5863E+01	4.8847E+01	3.3712E+01	3.4980E+01	6.2813E+01
	Rank	1	7	2	6	5	3	4
F24	Best	2.8834E+03	2.8838E+03	2.8862E+03	2.8841E+03	2.8849E+03	2.8921E+03	2.8871E+03
	Mean	2.8872E+03	2.8879E+03	2.8962E+03	2.9070E+03	2.9016E+03	2.9206E+03	2.8877E+03
	Std	2.1901E+00	2.1579E+00	1.4599E+01	2.0207E+01	1.4822E+01	2.0611E+01	3.4355E-01
	Rank	1	3	4	6	5	7	2
F25	Best	4.0280E+03	5.1156E+03	2.9075E+03	2.8380E+03	2.8015E+03	2.8610E+03	3.6797E+03
	Mean	4.3714E+03	5.5703E+03	4.4844E+03	5.4099E+03	4.5780E+03	4.5991E+03	3.8243E+03
	Std	2.2448E+02	2.0210E+02	3.7750E+02	8.5690E+02	1.0312E+03	7.3758E+02	2.6358E+02
	Rank	2	7	3	6	4	5	1
F26	Best	3.1742E+03	3.2132E+03	3.2022E+03	3.2091E+03	3.1989E+03	3.2371E+03	3.1975E+03
	Mean	3.2206E+03	3.2391E+03	3.2176E+03	3.2524E+03	3.2572E+03	3.2764E+03	3.2118E+03
	Std	1.8197E+01	1.3575E+01	9.1648E+00	2.9959E+01	1.9148E+01	1.8478E+01	6.0569E+00

	Rank	3	4	2	5	6	7	1
F27	Best	3.1000E+03	3.1902E+03	3.2255E+03	3.2013E+03	3.1225E+03	3.2414E+03	3.1977E+03
	Mean	3.1318E+03	3.2197E+03	3.2703E+03	3.2563E+03	3.2307E+03	3.2937E+03	3.2110E+03
	Std	5.4952E+01	1.6162E+01	3.7359E+01	2.8210E+01	2.9823E+01	3.9531E+01	1.1908E+01
	Rank	1	3	6	5	4	7	2
F28	Best	3.2642E+03	3.6779E+03	3.4981E+03	3.6039E+03	3.4601E+03	3.5066E+03	3.5816E+03
	Mean	3.4428E+03	3.8739E+03	3.7542E+03	4.1510E+03	3.7798E+03	3.6910E+03	3.8335E+03
	Std	1.2868E+02	1.2664E+02	1.9143E+02	2.6096E+02	2.0075E+02	1.6123E+02	8.7887E+01
	Rank	1	6	3	7	4	2	5
F29	Best	4.9516E+03	1.1870E+04	6.4998E+04	8.7855E+03	7.8723E+03	2.7071E+04	8.9763E+03
	Mean	5.0560E+03	2.7493E+04	6.1923E+05	4.1057E+04	1.6487E+04	9.3183E+04	1.5160E+04
	Std	6.7568E+01	1.3012E+04	5.5510E+05	5.5685E+04	8.4552E+03	6.1468E+04	5.1644E+03
	Rank	1	4	7	5	3	6	2

Table S5. The statistics results of MSEDO and basic algorithms solving CEC2017 (D=50)

No.	Index	MSEDO	EDO	RIME	ECO	MRFO	QIO	AE
F1	Best	1.0013E+02	4.0728E+03	4.0942E+06	5.0154E+06	1.1063E+05	5.1162E+07	2.7238E+03
	Mean	3.2584E+03	6.0924E+04	8.9937E+06	1.6263E+07	3.1968E+05	8.9428E+07	5.0636E+03
	Std	3.6554E+03	7.3973E+04	3.8162E+06	1.6940E+07	1.5546E+05	2.4975E+07	1.5976E+03
	Rank	1	3	5	6	4	7	2
F2	Best	3.0000E+02	1.4211E+05	2.4222E+04	1.8507E+04	6.2300E+04	9.7159E+04	1.9134E+04
	Mean	3.0000E+02	1.9863E+05	3.9210E+04	2.9561E+04	8.7309E+04	1.3341E+05	2.8298E+04
	Std	1.5309E-06	2.7624E+04	1.0451E+04	7.2863E+03	1.4925E+04	1.7400E+04	5.7629E+03
	Rank	1	7	4	3	5	6	2
F3	Best	4.0000E+02	4.3639E+02	5.3557E+02	5.0429E+02	4.5184E+02	6.0860E+02	5.2407E+02
	Mean	4.2879E+02	5.4903E+02	6.1327E+02	6.0427E+02	5.7759E+02	6.8439E+02	5.9190E+02
	Std	4.2571E+01	4.4080E+01	4.2098E+01	5.6488E+01	5.3633E+01	4.1570E+01	2.9937E+01
	Rank	1	2	6	5	3	7	4
F4	Best	5.9950E+02	8.0275E+02	5.8959E+02	6.9025E+02	6.9702E+02	6.2420E+02	5.3390E+02
	Mean	6.9071E+02	8.8399E+02	6.5161E+02	7.9418E+02	7.9664E+02	7.1130E+02	7.4621E+02
	Std	7.7283E+01	3.0885E+01	3.1434E+01	4.7059E+01	5.2915E+01	4.9676E+01	1.3392E+02
	Rank	2	7	1	5	6	3	4
F5	Best	6.0006E+02	6.0399E+02	6.0333E+02	6.4206E+02	6.0573E+02	6.0774E+02	6.0004E+02
	Mean	6.0427E+02	6.2258E+02	6.0648E+02	6.5277E+02	6.2652E+02	6.1385E+02	6.0018E+02
	Std	4.0040E+00	1.7343E+01	2.2562E+00	5.9100E+00	1.1606E+01	4.1509E+00	1.0660E-01
	Rank	2	5	3	7	6	4	1
F6	Best	8.6227E+02	1.1062E+03	8.8880E+02	1.1024E+03	9.7004E+02	1.0444E+03	7.7622E+02
	Mean	1.0693E+03	1.1951E+03	9.6319E+02	1.3251E+03	1.1555E+03	1.1550E+03	1.0261E+03
	Std	9.0640E+01	4.8507E+01	3.5837E+01	1.2596E+02	1.0712E+02	4.1092E+01	1.1316E+02
	Rank	3	6	1	7	5	4	2
F7	Best	8.8557E+02	1.0822E+03	8.8216E+02	9.6136E+02	9.3635E+02	9.4303E+02	8.2835E+02
	Mean	9.7726E+02	1.1621E+03	9.4138E+02	1.0835E+03	1.0854E+03	1.0044E+03	9.6907E+02
	Std	7.2787E+01	3.1966E+01	2.5019E+01	5.1539E+01	7.2747E+01	3.4748E+01	1.3824E+02
	Rank	3	7	1	5	6	4	2
F8	Best	9.0045E+02	2.4541E+03	1.2929E+03	6.3566E+03	6.9692E+03	1.4217E+03	9.0010E+02
	Mean	1.8929E+03	9.6788E+03	2.4205E+03	1.2034E+04	1.2392E+04	3.0953E+03	9.0265E+02
	Std	1.7664E+03	3.8539E+03	1.1650E+03	2.9699E+03	3.9282E+03	1.2892E+03	1.8581E+00
	Rank	2	5	3	6	7	4	1
F9	Best	1.0398E+04	1.0142E+04	5.0340E+03	6.4800E+03	5.7195E+03	1.2599E+04	1.2999E+04

	Mean	1.1151E+04	1.1138E+04	7.2050E+03	8.5900E+03	6.9221E+03	1.4519E+04	1.4424E+04
	Std	3.1728E+02	5.6569E+02	7.5479E+02	1.1849E+03	8.0247E+02	5.8957E+02	4.4780E+02
	Rank	5	4	2	3	1	7	6
F10	Best	1.1352E+03	2.3384E+03	1.3762E+03	1.2560E+03	1.2224E+03	1.3485E+03	1.2035E+03
	Mean	1.2296E+03	3.4394E+03	1.5182E+03	1.3887E+03	1.3297E+03	1.5074E+03	1.2730E+03
	Std	3.3379E+01	6.5475E+02	7.9586E+01	7.2790E+01	4.9108E+01	8.2711E+01	2.9723E+01
	Rank	1	7	6	4	3	5	2
F11	Best	4.4898E+03	1.9074E+06	8.7356E+06	4.7842E+06	9.2330E+05	1.2930E+07	9.4488E+05
	Mean	1.1372E+04	4.8349E+06	7.2983E+07	1.5629E+07	3.2195E+06	2.2923E+07	1.8442E+06
	Std	1.2310E+04	2.0424E+06	4.3226E+07	8.5469E+06	1.9674E+06	8.6180E+06	3.4724E+05
	Rank	1	4	7	5	3	6	2
F12	Best	1.4853E+03	1.7861E+03	9.8501E+04	2.6943E+04	1.7091E+03	1.4239E+04	5.5991E+03
	Mean	6.0416E+03	7.1538E+03	2.2851E+05	1.0368E+05	7.3440E+03	3.3914E+04	8.6114E+03
	Std	4.9066E+03	6.1075E+03	1.0842E+05	4.9962E+04	5.9630E+03	1.4380E+04	1.4337E+03
	Rank	1	2	7	6	3	5	4
F13	Best	1.4788E+03	2.2616E+05	1.9494E+04	2.1738E+03	6.4097E+03	3.8262E+04	2.3399E+03
	Mean	1.5040E+03	4.8320E+05	1.5931E+05	3.9491E+04	7.1892E+04	1.3174E+05	4.3126E+03
	Std	8.0550E+00	1.7139E+05	9.0870E+04	4.2010E+04	6.0629E+04	9.3739E+04	1.9599E+03
	Rank	1	7	6	3	4	5	2
F14	Best	1.6136E+03	1.7445E+03	2.0475E+04	6.4458E+03	1.6950E+03	2.3711E+03	1.9744E+03
	Mean	3.2448E+03	1.5307E+04	8.5959E+04	3.1271E+04	9.2206E+03	5.0251E+03	2.3730E+03
	Std	2.3423E+03	8.1859E+03	4.5829E+04	1.8345E+04	7.1836E+03	3.1623E+03	4.2803E+02
	Rank	2	5	7	6	4	3	1
F15	Best	2.1484E+03	4.0166E+03	2.7179E+03	2.9462E+03	2.4310E+03	1.9543E+03	2.2413E+03
	Mean	2.8262E+03	4.5182E+03	3.3113E+03	3.5876E+03	3.2781E+03	2.8174E+03	3.5771E+03
	Std	4.8980E+02	2.5510E+02	3.7940E+02	4.2754E+02	4.1983E+02	3.0530E+02	5.0605E+02
	Rank	2	7	4	6	3	1	5
F16	Best	2.1500E+03	2.4026E+03	2.2767E+03	2.6813E+03	2.2927E+03	2.1402E+03	2.7791E+03
	Mean	2.8850E+03	3.3866E+03	2.8955E+03	3.3383E+03	2.9951E+03	2.7795E+03	3.4096E+03
	Std	5.0863E+02	2.9227E+02	3.0382E+02	3.2671E+02	3.7720E+02	2.7503E+02	2.6266E+02
	Rank	2	6	3	5	4	1	7
F17	Best	1.8420E+03	5.5410E+05	1.4321E+05	6.2846E+04	6.3386E+04	2.1426E+05	3.0516E+04
	Mean	1.8536E+03	2.1052E+06	2.0388E+06	3.7692E+05	8.8274E+05	1.1939E+06	7.0679E+04
	Std	7.8626E+00	1.4635E+06	1.4102E+06	3.4184E+05	9.5758E+05	7.7393E+05	2.6814E+04
	Rank	1	7	6	3	4	5	2
F18	Best	1.9272E+03	2.0132E+03	3.4395E+04	7.1826E+03	1.9855E+03	2.4487E+03	7.9224E+03
	Mean	2.3713E+03	8.1301E+03	2.5526E+05	3.6568E+04	1.4841E+04	1.4363E+04	1.1894E+04
	Std	1.2144E+03	1.0576E+04	2.4679E+05	1.6259E+04	1.1284E+04	7.9816E+03	2.2635E+03
	Rank	1	2	7	6	5	4	3
F19	Best	2.3849E+03	3.2019E+03	2.3247E+03	2.5307E+03	2.3469E+03	2.3220E+03	3.2256E+03
	Mean	3.0903E+03	3.6338E+03	2.9331E+03	3.0978E+03	2.9374E+03	3.2159E+03	3.6512E+03
	Std	3.1807E+02	1.7678E+02	2.9137E+02	2.5546E+02	2.9479E+02	4.3567E+02	1.3115E+02
	Rank	3	6	1	4	2	5	7
F20	Best	2.3784E+03	2.6312E+03	2.3978E+03	2.5082E+03	2.4370E+03	2.4368E+03	2.3310E+03
	Mean	2.4679E+03	2.6817E+03	2.4555E+03	2.6050E+03	2.5281E+03	2.4990E+03	2.5239E+03
	Std	6.6396E+01	3.6399E+01	3.5462E+01	7.9856E+01	4.4709E+01	3.9331E+01	1.2150E+02
	Rank	2	7	1	6	5	3	4
F21	Best	5.0645E+03	1.1947E+04	7.1354E+03	2.3446E+03	2.3053E+03	2.3831E+03	2.3043E+03
	Mean	1.2716E+04	1.3838E+04	8.6816E+03	1.0257E+04	8.5635E+03	5.1204E+03	1.1029E+04

	Std	1.4890E+03	5.1002E+02	8.8790E+02	1.8897E+03	1.9697E+03	5.1221E+03	6.3533E+03
	Rank	6	7	3	4	2	1	5
F22	Best	2.7843E+03	3.0764E+03	2.8545E+03	2.9635E+03	2.8597E+03	2.9507E+03	2.7543E+03
	Mean	2.8860E+03	3.1481E+03	2.9074E+03	3.1189E+03	2.9933E+03	3.0484E+03	2.8216E+03
	Std	5.7869E+01	3.1446E+01	4.4851E+01	9.8067E+01	7.7754E+01	6.3117E+01	1.0767E+02
	Rank	2	7	3	6	4	5	1
F23	Best	2.9958E+03	3.2799E+03	2.9977E+03	3.1305E+03	3.1158E+03	3.1023E+03	2.9096E+03
	Mean	3.0790E+03	3.3383E+03	3.0663E+03	3.3164E+03	3.2096E+03	3.1965E+03	3.0098E+03
	Std	8.1016E+01	3.4741E+01	3.5088E+01	1.0888E+02	6.6464E+01	6.4632E+01	1.2157E+02
	Rank	3	7	2	6	5	4	1
F24	Best	2.9287E+03	2.9882E+03	3.0302E+03	3.0555E+03	3.0450E+03	3.1166E+03	3.0319E+03
	Mean	3.0162E+03	3.0582E+03	3.0767E+03	3.1214E+03	3.1020E+03	3.1743E+03	3.0697E+03
	Std	4.1608E+01	2.5467E+01	3.5373E+01	3.7653E+01	2.6867E+01	3.7754E+01	2.2321E+01
	Rank	1	2	4	6	5	7	3
F25	Best	4.5769E+03	7.0641E+03	2.9306E+03	3.3588E+03	2.9190E+03	3.7619E+03	4.0030E+03
	Mean	5.5286E+03	7.7959E+03	5.4178E+03	7.6329E+03	5.6057E+03	6.3967E+03	4.5166E+03
	Std	4.8108E+02	3.6907E+02	6.7026E+02	1.5626E+03	3.2068E+03	1.3603E+03	7.1364E+02
	Rank	3	7	2	6	4	5	1
F26	Best	3.2347E+03	3.4426E+03	3.3109E+03	3.2851E+03	3.3039E+03	3.4450E+03	3.2344E+03
	Mean	3.3484E+03	3.7059E+03	3.4109E+03	3.5747E+03	3.5889E+03	3.6573E+03	3.2715E+03
	Std	8.4625E+01	1.6269E+02	6.6990E+01	1.5928E+02	1.4470E+02	9.4629E+01	3.6527E+01
	Rank	2	7	3	4	5	6	1
F27	Best	3.2534E+03	3.2659E+03	3.3147E+03	3.3228E+03	3.3044E+03	3.4174E+03	3.2779E+03
	Mean	3.2811E+03	3.3320E+03	3.3681E+03	3.7564E+03	3.3797E+03	3.5042E+03	3.3232E+03
	Std	2.2219E+01	2.8095E+01	3.5308E+01	1.3404E+03	4.1989E+01	5.9588E+01	1.7519E+01
	Rank	1	3	4	7	5	6	2
F28	Best	3.2421E+03	4.1706E+03	3.6958E+03	4.3705E+03	3.6540E+03	3.7721E+03	3.7553E+03
	Mean	3.7881E+03	4.7416E+03	4.3267E+03	5.0770E+03	4.2837E+03	4.2574E+03	4.4075E+03
	Std	2.9583E+02	4.1695E+02	3.3820E+02	4.0968E+02	4.2850E+02	3.2001E+02	3.0526E+02
	Rank	1	6	4	7	3	2	5
F29	Best	5.8888E+05	2.1555E+06	1.8459E+07	1.8390E+06	1.5162E+06	5.5971E+06	8.1430E+05
	Mean	6.7410E+05	3.2409E+06	2.9521E+07	3.5372E+06	2.1479E+06	8.1373E+06	9.9018E+05
	Std	1.0825E+05	8.0587E+05	7.4476E+06	1.2685E+06	5.5297E+05	1.7537E+06	9.7545E+04
	Rank	1	4	7	5	3	6	2

Table S6. The statistics results of MSEDO and basic algorithms solving CEC2017 (D=100)

No.	Index	MSEDO	EDO	RIME	ECO	MRFO	QIO	AE
F1	Best	1.0913E+02	1.5586E+06	5.8311E+07	1.5411E+08	1.9143E+07	7.5725E+08	8.0534E+04
	Mean	5.5156E+03	1.0813E+07	9.7914E+07	4.7562E+08	5.8840E+07	9.7929E+08	1.3473E+05
	Std	5.6516E+03	1.0848E+07	2.6887E+07	3.2221E+08	3.6589E+07	1.4752E+08	4.8400E+04
	Rank	1	3	5	6	4	7	2
F2	Best	3.0000E+02	4.2148E+05	1.6879E+05	9.7872E+04	1.8450E+05	2.7605E+05	9.2869E+04
	Mean	3.0000E+02	5.2850E+05	2.2829E+05	1.3274E+05	2.2519E+05	3.3825E+05	1.1439E+05
	Std	2.8577E-04	5.6824E+04	3.1423E+04	1.7417E+04	2.3741E+04	2.6862E+04	1.1974E+04
	Rank	1	7	5	3	4	6	2
F3	Best	4.0000E+02	7.1766E+02	7.3673E+02	8.4797E+02	7.9343E+02	1.0037E+03	6.6685E+02
	Mean	4.3858E+02	8.0698E+02	8.5483E+02	9.9967E+02	9.4433E+02	1.1635E+03	7.1318E+02
	Std	6.1311E+01	4.7583E+01	4.9589E+01	9.8631E+01	6.2070E+01	1.0782E+02	2.4904E+01
	Rank	1	3	4	6	5	7	2

F4	Best	7.3083E+02	1.3953E+03	7.9975E+02	1.0636E+03	1.0447E+03	9.8287E+02	6.1579E+02
	Mean	9.9878E+02	1.4976E+03	9.0143E+02	1.2240E+03	1.2281E+03	1.0970E+03	9.9634E+02
	Std	1.8964E+02	5.4242E+01	5.6901E+01	7.9473E+01	8.9004E+01	6.2350E+01	3.3185E+02
	Rank	3	7	1	5	6	4	2
F5	Best	6.0185E+02	6.2317E+02	6.1061E+02	6.4513E+02	6.3374E+02	6.2059E+02	6.0054E+02
	Mean	6.1496E+02	6.4858E+02	6.1834E+02	6.6050E+02	6.4933E+02	6.3055E+02	6.0104E+02
	Std	8.5638E+00	2.0334E+01	4.1175E+00	7.4530E+00	7.7386E+00	4.9834E+00	3.7643E-01
	Rank	2	5	3	7	6	4	1
F6	Best	1.2853E+03	1.8401E+03	1.2616E+03	2.1159E+03	1.7230E+03	1.6947E+03	9.5795E+02
	Mean	1.7735E+03	2.0744E+03	1.4879E+03	2.6222E+03	2.1756E+03	1.8208E+03	1.4610E+03
	Std	2.7391E+02	1.2875E+02	1.0785E+02	2.7924E+02	3.0670E+02	6.7802E+01	2.7398E+02
	Rank	3	5	2	7	6	4	1
F7	Best	1.0388E+03	1.6774E+03	1.0870E+03	1.3713E+03	1.4377E+03	1.3240E+03	9.0862E+02
	Mean	1.3408E+03	1.8237E+03	1.1884E+03	1.5884E+03	1.6435E+03	1.4176E+03	1.1677E+03
	Std	2.4984E+02	7.4271E+01	5.8567E+01	1.2821E+02	1.1084E+02	5.1143E+01	3.0800E+02
	Rank	3	7	2	5	6	4	1
F8	Best	1.3888E+03	2.9244E+04	4.6869E+03	2.2976E+04	2.1231E+04	8.3680E+03	9.1372E+02
	Mean	1.0011E+04	4.7430E+04	1.0562E+04	2.9195E+04	2.7784E+04	1.8275E+04	9.6043E+02
	Std	8.1509E+03	8.8267E+03	5.5093E+03	3.5803E+03	6.8676E+03	7.0921E+03	3.0940E+01
	Rank	2	7	3	6	5	4	1
F9	Best	2.5350E+04	2.6317E+04	1.3887E+04	1.4547E+04	1.2289E+04	2.1984E+04	2.9512E+04
	Mean	2.7362E+04	2.7406E+04	1.6120E+04	1.8652E+04	1.5814E+04	3.0366E+04	3.0866E+04
	Std	6.8810E+02	5.2420E+02	1.1889E+03	2.1633E+03	1.9600E+03	2.3589E+03	6.4150E+02
	Rank	4	5	2	3	1	6	7
F10	Best	1.5587E+03	3.3086E+04	3.4724E+03	3.1519E+03	1.4272E+04	2.2898E+04	3.6631E+03
	Mean	1.7078E+03	7.2993E+04	4.1840E+03	4.4960E+03	2.4469E+04	3.9297E+04	4.6929E+03
	Std	1.0992E+02	1.6438E+04	4.6972E+02	8.6236E+02	5.7590E+03	9.2391E+03	5.6254E+02
	Rank	1	7	2	3	5	6	4
F11	Best	2.5064E+04	6.8305E+06	1.7585E+08	4.2019E+07	1.3513E+07	1.2524E+08	6.7073E+06
	Mean	5.3747E+04	3.1281E+07	5.5025E+08	8.8989E+07	3.9554E+07	2.4345E+08	1.2892E+07
	Std	2.0065E+04	1.6817E+07	2.4688E+08	3.6541E+07	1.6554E+07	6.2197E+07	3.4017E+06
	Rank	1	3	7	5	4	6	2
F12	Best	1.4692E+03	2.4953E+03	1.4784E+05	1.2863E+04	2.6094E+03	4.6194E+04	1.2626E+04
	Mean	6.8615E+03	5.1882E+03	5.7472E+05	2.4955E+04	9.4972E+03	9.0204E+04	1.8049E+04
	Std	5.0932E+03	2.8914E+03	1.6566E+06	1.2332E+04	8.1315E+03	2.6270E+04	2.6042E+03
	Rank	2	1	7	5	3	6	4
F13	Best	1.6446E+03	2.1961E+06	9.1692E+05	1.3602E+05	2.9790E+05	3.6436E+05	9.7411E+04
	Mean	1.6828E+03	5.4510E+06	2.4834E+06	4.7103E+05	9.2209E+05	1.0823E+06	1.7225E+05
	Std	1.5503E+01	2.2058E+06	9.1303E+05	3.2440E+05	3.8324E+05	4.8796E+05	5.8081E+04
	Rank	1	7	6	3	4	5	2
F14	Best	1.7211E+03	1.8750E+03	5.6477E+04	4.0960E+03	1.9801E+03	5.2701E+03	4.3676E+03
	Mean	4.6235E+03	4.8927E+03	1.2339E+05	1.1988E+04	5.1322E+03	9.5661E+03	6.7341E+03
	Std	4.0545E+03	3.9883E+03	5.6918E+04	1.2969E+04	3.7994E+03	3.8931E+03	1.8006E+03
	Rank	1	2	7	6	3	5	4
F15	Best	2.2939E+03	4.6297E+03	4.3429E+03	5.0486E+03	4.6804E+03	3.7927E+03	4.1901E+03
	Mean	4.9053E+03	8.7882E+03	5.8414E+03	6.4050E+03	5.5825E+03	5.2791E+03	7.1651E+03
	Std	1.8254E+03	1.0461E+03	8.1127E+02	6.3387E+02	6.5659E+02	7.2474E+02	1.5670E+03
	Rank	1	7	4	5	3	2	6
F16	Best	3.0024E+03	5.2287E+03	4.1605E+03	4.4199E+03	3.4657E+03	3.2585E+03	4.9996E+03

	Mean	5.0339E+03	6.3438E+03	5.0755E+03	5.4174E+03	4.7540E+03	4.2761E+03	6.2764E+03
	Std	1.0975E+03	4.0154E+02	4.7454E+02	5.5729E+02	6.5591E+02	5.2634E+02	4.8147E+02
	Rank	3	7	4	5	2	1	6
F17	Best	1.9330E+03	4.1028E+06	1.8888E+06	2.6014E+05	3.2469E+05	9.1623E+05	1.6992E+05
	Mean	1.9559E+03	1.4862E+07	4.3441E+06	6.9038E+05	1.4717E+06	1.9872E+06	3.0589E+05
	Std	1.2776E+01	6.4769E+06	1.7747E+06	2.6894E+05	7.2611E+05	7.7524E+05	8.2470E+04
	Rank	1	7	6	3	4	5	2
F18	Best	1.9946E+03	2.1666E+03	2.6555E+05	2.8044E+03	2.0591E+03	4.1461E+03	2.5095E+03
	Mean	8.6803E+03	7.5659E+03	4.2879E+06	8.7625E+03	5.7425E+03	9.2471E+03	3.1946E+03
	Std	6.6548E+03	6.3150E+03	3.1510E+06	6.4952E+03	4.0952E+03	5.0124E+03	4.4180E+02
	Rank	4	3	7	5	2	6	1
F19	Best	5.3994E+03	5.7532E+03	3.9966E+03	4.3563E+03	3.9227E+03	4.0479E+03	6.3832E+03
	Mean	6.4559E+03	6.4822E+03	5.0868E+03	5.3302E+03	4.9854E+03	6.4784E+03	6.7777E+03
	Std	3.3349E+02	4.1746E+02	4.9442E+02	4.7541E+02	5.6984E+02	1.0229E+03	1.8412E+02
	Rank	4	6	2	3	1	5	7
F20	Best	2.5762E+03	3.2391E+03	2.6308E+03	2.9000E+03	2.7563E+03	2.8194E+03	2.4250E+03
	Mean	2.8963E+03	3.3706E+03	2.7566E+03	3.2185E+03	2.9707E+03	2.9725E+03	2.7866E+03
	Std	2.4950E+02	6.9273E+01	7.9581E+01	1.5008E+02	9.6051E+01	1.0288E+02	3.1021E+02
	Rank	3	7	1	6	4	5	2
F21	Best	2.8692E+04	2.7228E+04	1.5322E+04	1.8956E+04	1.6321E+04	2.6843E+03	3.1436E+04
	Mean	2.9926E+04	2.8952E+04	1.8504E+04	2.2166E+04	1.8898E+04	2.2947E+04	3.2861E+04
	Std	4.8877E+02	6.7121E+02	1.2801E+03	1.8540E+03	1.6258E+03	1.3552E+04	6.4630E+02
	Rank	6	5	1	3	2	4	7
F22	Best	3.1244E+03	3.7857E+03	3.1513E+03	3.4708E+03	3.3901E+03	3.6536E+03	2.9476E+03
	Mean	3.2641E+03	3.9575E+03	3.2361E+03	3.7434E+03	3.5948E+03	3.8092E+03	2.9914E+03
	Std	8.5084E+01	6.2468E+01	5.0307E+01	1.4142E+02	1.0194E+02	9.7606E+01	2.1882E+01
	Rank	3	7	2	5	4	6	1
F23	Best	3.4807E+03	4.2957E+03	3.5775E+03	4.1642E+03	4.0862E+03	4.1648E+03	3.3577E+03
	Mean	3.7464E+03	4.4823E+03	3.7230E+03	4.4475E+03	4.3156E+03	4.3999E+03	3.3969E+03
	Std	1.4054E+02	8.6097E+01	6.8044E+01	1.9161E+02	1.7241E+02	1.0993E+02	2.5997E+01
	Rank	3	7	2	6	4	5	1
F24	Best	3.1372E+03	3.3339E+03	3.3921E+03	3.5784E+03	3.4910E+03	3.6195E+03	3.3000E+03
	Mean	3.2328E+03	3.4740E+03	3.5797E+03	3.7090E+03	3.6257E+03	3.8143E+03	3.4051E+03
	Std	5.4325E+01	6.6301E+01	7.9151E+01	7.9112E+01	6.6125E+01	9.7170E+01	3.6854E+01
	Rank	1	3	4	6	5	7	2
F25	Best	8.5685E+03	1.2147E+04	9.1177E+03	1.5050E+04	7.4637E+03	1.2763E+04	6.6797E+03
	Mean	1.0285E+04	1.7055E+04	1.0420E+04	1.7998E+04	1.8897E+04	1.5042E+04	7.3263E+03
	Std	1.3210E+03	1.1837E+03	8.3763E+02	2.0166E+03	3.5315E+03	1.9215E+03	2.6829E+02
	Rank	2	5	3	6	7	4	1
F26	Best	3.3863E+03	3.6509E+03	3.4695E+03	3.5011E+03	3.7409E+03	3.7390E+03	3.3397E+03
	Mean	3.4910E+03	3.8614E+03	3.5951E+03	3.7992E+03	3.8875E+03	3.9302E+03	3.3926E+03
	Std	7.8241E+01	1.3643E+02	6.9800E+01	1.7880E+02	1.1956E+02	9.0190E+01	2.4649E+01
	Rank	2	5	3	4	6	7	1
F27	Best	3.2620E+03	3.4953E+03	3.5531E+03	3.5926E+03	3.6320E+03	3.7438E+03	3.4344E+03
	Mean	3.3381E+03	3.6254E+03	3.6536E+03	3.7442E+03	3.7977E+03	3.9399E+03	3.4753E+03
	Std	4.9672E+01	7.6599E+01	5.6245E+01	7.2996E+01	9.8708E+01	1.0351E+02	1.7654E+01
	Rank	1	3	4	5	6	7	2
F28	Best	4.1747E+03	5.9941E+03	5.8327E+03	6.8130E+03	5.9467E+03	5.7146E+03	5.9040E+03
	Mean	5.7039E+03	8.4348E+03	7.3018E+03	7.8147E+03	7.0116E+03	6.8711E+03	7.7459E+03

	Std	7.2025E+02	1.1455E+03	5.3835E+02	5.9645E+02	6.4379E+02	5.2793E+02	8.5837E+02
	Rank	1	7	4	6	3	2	5
F29	Best	5.8090E+03	4.1378E+04	1.8817E+07	1.2781E+05	2.4307E+04	8.9442E+05	8.9647E+04
	Mean	4.3415E+04	1.8021E+05	4.7295E+07	4.7825E+05	1.0681E+05	1.8244E+06	1.8640E+05
	Std	1.6219E+05	1.3755E+05	1.6593E+07	2.7946E+05	7.5390E+04	7.2162E+05	6.2047E+04
	Rank	1	3	7	5	2	6	4

Table S7. The statistics results of MSEDO and basic algorithms solving CEC2022 (D=10)

No.	Index	MSEDO	EDO	RIME	ECO	MRFO	QIO	AE
F1	Best	3.0000E+02	8.7876E+02	3.0003E+02	3.0000E+02	3.0687E+02	3.7014E+02	3.6336E+02
	Mean	3.0000E+02	2.2632E+03	3.0093E+02	3.0017E+02	3.4589E+02	6.8992E+02	4.5810E+02
	Std	4.3522E-14	8.3485E+02	9.2844E-01	2.8774E-01	3.6215E+01	2.5012E+02	9.1790E+01
	Rank	1	7	3	2	4	6	5
F2	Best	4.0000E+02	4.0000E+02	4.0010E+02	4.0000E+02	4.0000E+02	4.0000E+02	4.0006E+02
	Mean	4.0485E+02	4.0503E+02	4.0773E+02	4.0893E+02	4.0895E+02	4.0976E+02	4.0555E+02
	Std	1.2778E+01	3.3098E+00	1.3006E+01	1.1777E+01	2.0607E+01	2.0321E+01	4.0250E+00
	Rank	1	2	4	5	6	7	3
F3	Best	6.0000E+02	6.0000E+02	6.0005E+02	6.0015E+02	6.0000E+02	6.0014E+02	6.0002E+02
	Mean	6.0056E+02	6.0004E+02	6.0023E+02	6.0600E+02	6.0006E+02	6.0059E+02	6.0003E+02
	Std	1.4918E+00	5.9272E-02	1.6339E-01	5.8912E+00	2.5772E-01	3.4657E-01	9.0982E-03
	Rank	5	2	4	7	3	6	1
F4	Best	8.0298E+02	8.1318E+02	8.0500E+02	8.0697E+02	8.0597E+02	8.0307E+02	8.0444E+02
	Mean	8.1907E+02	8.2380E+02	8.1727E+02	8.2236E+02	8.1844E+02	8.1540E+02	8.2322E+02
	Std	1.1159E+01	5.6819E+00	7.1954E+00	1.0853E+01	7.2727E+00	7.7032E+00	8.8875E+00
	Rank	4	7	2	5	3	1	6
F5	Best	9.0000E+02	9.0000E+02	9.0001E+02	9.0054E+02	9.0000E+02	9.0001E+02	9.0000E+02
	Mean	9.0035E+02	9.0009E+02	9.0021E+02	9.7620E+02	9.0129E+02	9.0016E+02	9.0000E+02
	Std	1.1638E+00	2.0934E-01	2.7217E-01	1.0343E+02	4.7911E+00	1.8524E-01	5.0601E-04
	Rank	5	2	4	7	6	3	1
F6	Best	1.8000E+03	2.0045E+03	1.9232E+03	1.8429E+03	1.8240E+03	1.8458E+03	1.9679E+03
	Mean	1.8022E+03	4.6887E+03	3.6348E+03	2.3752E+03	3.2919E+03	2.0586E+03	2.3071E+03
	Std	6.3734E+00	1.8429E+03	1.8261E+03	1.3399E+03	1.3804E+03	3.0099E+02	2.7356E+02
	Rank	1	7	6	4	5	2	3
F7	Best	2.0000E+03	2.0144E+03	2.0005E+03	2.0059E+03	2.0015E+03	2.0136E+03	2.0221E+03
	Mean	2.0149E+03	2.0265E+03	2.0173E+03	2.0351E+03	2.0141E+03	2.0268E+03	2.0366E+03
	Std	2.2938E+01	3.8551E+00	8.3008E+00	1.6600E+01	8.6399E+00	6.2885E+00	5.8898E+00
	Rank	2	4	3	6	1	5	7
F8	Best	2.2017E+03	2.2113E+03	2.2012E+03	2.2069E+03	2.2012E+03	2.2137E+03	2.2245E+03
	Mean	2.2337E+03	2.2228E+03	2.2183E+03	2.2243E+03	2.2231E+03	2.2267E+03	2.2289E+03
	Std	4.2763E+01	3.3278E+00	7.5104E+00	6.9006E+00	7.4220E+00	4.4143E+00	1.8376E+00
	Rank	7	2	1	4	3	5	6
F9	Best	2.4000E+03	2.5293E+03	2.5293E+03	2.5293E+03	2.5293E+03	2.5301E+03	2.5293E+03
	Mean	2.5207E+03	2.5293E+03	2.5293E+03	2.5342E+03	2.5294E+03	2.5312E+03	2.5293E+03
	Std	3.2800E+01	2.5059E-02	4.5358E-03	2.6826E+01	6.2637E-02	9.6843E-01	3.4177E-03
	Rank	1	4	2	7	5	6	3
F10	Best	2.4070E+03	2.5003E+03	2.5002E+03	2.5003E+03	2.5002E+03	2.5003E+03	2.5002E+03
	Mean	2.5015E+03	2.5004E+03	2.5077E+03	2.5284E+03	2.5046E+03	2.5005E+03	2.5004E+03
	Std	2.7193E+01	5.7553E-02	2.7924E+01	5.1441E+01	2.2893E+01	1.1043E-01	7.8214E-02
	Rank	4	1	6	7	5	3	2

F11	Best	2.6000E+03	2.6001E+03	2.6099E+03	2.6001E+03	2.6000E+03	2.6021E+03	2.7550E+03
	Mean	2.8487E+03	2.8701E+03	2.8816E+03	2.7151E+03	2.6970E+03	2.6277E+03	2.8979E+03
	Std	1.1799E+02	9.1419E+01	1.3127E+02	1.7803E+02	1.5245E+02	4.9151E+01	2.7010E+01
	Rank	4	5	6	3	2	1	7
F12	Best	2.8639E+03	2.8602E+03	2.8597E+03	2.8614E+03	2.8627E+03	2.8639E+03	2.8638E+03
	Mean	2.8664E+03	2.8643E+03	2.8641E+03	2.8639E+03	2.8676E+03	2.8671E+03	2.8649E+03
	Std	2.0092E+00	1.2616E+00	1.6810E+00	1.3868E+00	3.9393E+00	1.9174E+00	2.0063E-01
	Rank	5	3	2	1	7	6	4

Table S8. The statistics results of MSEDO and basic algorithms solving CEC2022 (D=20)

No.	Index	MSEDO	EDO	RIME	ECO	MRFO	QIO	AE
F1	Best	3.0000E+02	9.6051E+03	3.3493E+02	3.2946E+02	1.7475E+03	5.9728E+03	1.7094E+03
	Mean	3.0000E+02	2.1724E+04	3.8781E+02	6.2205E+02	5.6839E+03	9.9115E+03	2.8656E+03
	Std	9.0394E-08	5.6737E+03	3.6110E+01	3.0272E+02	2.2734E+03	2.5759E+03	8.1008E+02
	Rank	1	7	2	3	5	6	4
F2	Best	4.0000E+02	4.4490E+02	4.0114E+02	4.2936E+02	4.0128E+02	4.5154E+02	4.4911E+02
	Mean	4.3884E+02	4.5124E+02	4.4962E+02	4.6118E+02	4.5087E+02	4.6814E+02	4.4925E+02
	Std	1.9476E+01	7.4382E+00	1.6928E+01	2.5822E+01	1.6836E+01	1.8290E+01	1.2588E-01
	Rank	1	5	3	6	4	7	2
F3	Best	6.0000E+02	6.0010E+02	6.0043E+02	6.0883E+02	6.0003E+02	6.0118E+02	6.0007E+02
	Mean	6.0061E+02	6.0066E+02	6.0109E+02	6.2337E+02	6.0189E+02	6.0283E+02	6.0016E+02
	Std	1.5761E+00	7.1989E-01	5.2992E-01	9.8771E+00	3.4688E+00	1.0261E+00	1.0616E-01
	Rank	2	3	4	7	5	6	1
F4	Best	8.2089E+02	8.5664E+02	8.1818E+02	8.4191E+02	8.2985E+02	8.1573E+02	8.5004E+02
	Mean	8.4812E+02	8.8172E+02	8.4313E+02	8.7035E+02	8.5648E+02	8.4994E+02	8.9028E+02
	Std	1.8245E+01	1.2250E+01	1.3821E+01	1.8988E+01	1.5435E+01	2.3230E+01	1.1610E+01
	Rank	2	6	1	5	4	3	7
F5	Best	9.0000E+02	9.0056E+02	9.0110E+02	1.0249E+03	9.0091E+02	9.0103E+02	9.0000E+02
	Mean	9.4602E+02	9.2659E+02	9.2635E+02	1.8896E+03	1.3077E+03	9.0981E+02	9.0009E+02
	Std	1.5860E+02	6.5319E+01	6.4275E+01	4.4870E+02	4.8346E+02	1.0655E+01	1.4000E-01
	Rank	5	4	3	7	6	2	1
F6	Best	1.8002E+03	3.3424E+03	3.3343E+03	2.0418E+03	1.9694E+03	2.3326E+03	2.0253E+03
	Mean	1.8111E+03	1.5245E+04	1.5149E+04	1.0482E+04	6.7608E+03	9.1048E+03	2.7727E+03
	Std	2.2581E+01	1.2646E+04	7.4080E+03	7.1222E+03	4.4931E+03	5.5442E+03	6.7150E+02
	Rank	1	7	6	5	3	4	2
F7	Best	2.0149E+03	2.0463E+03	2.0253E+03	2.0489E+03	2.0247E+03	2.0432E+03	2.0815E+03
	Mean	2.0907E+03	2.0753E+03	2.0372E+03	2.1060E+03	2.0489E+03	2.0597E+03	2.0979E+03
	Std	8.7455E+01	1.4856E+01	9.2467E+00	3.5368E+01	1.7744E+01	1.3338E+01	7.7018E+00
	Rank	5	4	1	7	2	3	6
F8	Best	2.2225E+03	2.2272E+03	2.2222E+03	2.2259E+03	2.2242E+03	2.2289E+03	2.2298E+03
	Mean	2.2379E+03	2.2302E+03	2.2280E+03	2.2432E+03	2.2319E+03	2.2355E+03	2.2372E+03
	Std	3.5363E+01	1.6912E+00	3.7648E+00	3.3297E+01	2.2142E+01	3.5405E+00	3.2958E+00
	Rank	6	2	1	7	3	4	5
F9	Best	2.4808E+03	2.4808E+03	2.4808E+03	2.4808E+03	2.4811E+03	2.4830E+03	2.4808E+03
	Mean	2.4808E+03	2.4814E+03	2.4811E+03	2.4809E+03	2.4817E+03	2.4868E+03	2.4809E+03
	Std	6.7826E-07	6.0194E-01	2.1965E-01	1.1575E-01	3.7736E-01	3.0092E+00	5.7867E-02
	Rank	1	5	4	2	6	7	3
F10	Best	2.5002E+03	2.5005E+03	2.4366E+03	2.5006E+03	2.5004E+03	2.5005E+03	2.5004E+03
	Mean	2.8539E+03	3.8384E+03	2.5320E+03	2.9342E+03	2.5117E+03	2.5060E+03	2.5006E+03

	Std	5.8060E+02	9.9703E+02	9.4990E+01	6.6684E+02	4.1999E+01	2.8112E+01	9.4345E-02
	Rank	5	7	4	6	3	2	1
F11	Best	2.9000E+03	2.9000E+03	2.9562E+03	2.9111E+03	2.9001E+03	2.9351E+03	2.9187E+03
	Mean	2.9000E+03	2.9005E+03	3.0108E+03	2.9914E+03	2.9646E+03	2.9560E+03	2.9289E+03
	Std	2.6504E-11	5.2304E-01	2.7854E+01	1.1471E+02	1.0591E+02	1.1967E+01	4.1663E+00
	Rank	1	2	7	6	5	4	3
F12	Best	2.9351E+03	2.9421E+03	2.9357E+03	2.9441E+03	2.9436E+03	2.9726E+03	2.9342E+03
	Mean	2.9622E+03	2.9541E+03	2.9521E+03	2.9747E+03	2.9769E+03	2.9919E+03	2.9446E+03
	Std	3.0725E+01	1.0488E+01	8.2700E+00	2.5387E+01	1.8479E+01	1.2531E+01	5.9619E+00
	Rank	4	3	2	5	6	7	1

Table S9. The statistics results of MSEDO and improved algorithms solving CEC2017 (D=10)

No.	Index	MSEDO	EDO	IRIME	EMTLBO	EOSMA	MTVSCA	APSM-jSO
F1	Best	1.0001E+02	1.0014E+02	1.0205E+04	1.0691E+02	3.0248E+03	1.2993E+05	1.0017E+02
	Mean	1.0025E+02	3.5109E+03	4.0550E+04	8.1397E+02	2.5049E+04	4.5422E+05	1.0508E+02
	Std	1.2520E+00	2.7405E+03	3.1790E+04	8.6449E+02	1.7962E+04	2.8334E+05	1.3583E+01
	Rank	1	4	6	3	5	7	2
F2	Best	3.0000E+02	1.0202E+03	3.2729E+02	3.0000E+02	3.6337E+02	3.2946E+02	3.0000E+02
	Mean	3.0000E+02	3.1180E+03	4.4477E+02	3.0057E+02	6.0548E+02	4.4959E+02	3.0000E+02
	Std	9.9581E-14	1.5322E+03	1.2106E+02	1.4864E+00	1.7893E+02	1.0654E+02	4.0001E-05
	Rank	1	7	4	3	6	5	2
F3	Best	4.0000E+02	4.0243E+02	4.0113E+02	4.0000E+02	4.0403E+02	4.0342E+02	4.0016E+02
	Mean	4.0000E+02	4.0477E+02	4.0608E+02	4.0137E+02	4.0622E+02	4.0551E+02	4.0067E+02
	Std	8.6802E-10	7.3177E-01	1.5949E+00	8.7512E-01	8.5274E-01	8.7976E-01	3.3692E-01
	Rank	1	4	6	3	7	5	2
F4	Best	5.0398E+02	5.1360E+02	5.0400E+02	5.0981E+02	5.0740E+02	5.1808E+02	5.1214E+02
	Mean	5.2172E+02	5.2351E+02	5.1095E+02	5.2287E+02	5.1416E+02	5.2639E+02	5.2218E+02
	Std	2.3103E+01	4.9301E+00	3.8955E+00	6.2810E+00	3.2415E+00	4.7777E+00	5.3304E+00
	Rank	3	6	1	5	2	7	4
F5	Best	6.0000E+02	6.0000E+02	6.0004E+02	6.0000E+02	6.0005E+02	6.0056E+02	6.0001E+02
	Mean	6.0026E+02	6.0010E+02	6.0013E+02	6.0000E+02	6.0015E+02	6.0110E+02	6.0002E+02
	Std	1.0890E+00	3.0012E-01	5.8763E-02	8.1638E-04	7.7838E-02	3.4587E-01	7.7000E-03
	Rank	6	3	4	1	5	7	2
F6	Best	7.0533E+02	7.2212E+02	7.1485E+02	7.2583E+02	7.1753E+02	7.2610E+02	7.2899E+02
	Mean	7.2163E+02	7.3284E+02	7.2190E+02	7.3341E+02	7.2520E+02	7.4365E+02	7.4020E+02
	Std	9.1725E+00	6.1432E+00	4.3412E+00	4.6087E+00	5.1169E+00	7.2382E+00	5.2922E+00
	Rank	1	4	2	5	3	7	6
F7	Best	8.0398E+02	8.1182E+02	8.0501E+02	8.1812E+02	8.0441E+02	8.1909E+02	8.1299E+02
	Mean	8.2106E+02	8.2262E+02	8.1006E+02	8.2385E+02	8.1054E+02	8.2932E+02	8.2420E+02
	Std	1.1172E+01	4.6598E+00	3.9939E+00	3.4507E+00	3.9845E+00	5.1270E+00	5.4153E+00
	Rank	3	4	1	5	2	7	6
F8	Best	9.0000E+02	9.0000E+02	9.0001E+02	9.0000E+02	9.0001E+02	9.0040E+02	9.0000E+02
	Mean	9.0009E+02	9.0002E+02	9.0011E+02	9.0000E+02	9.0019E+02	9.0148E+02	9.0000E+02
	Std	1.9151E-01	8.4851E-02	9.9588E-02	4.7410E-07	3.3917E-01	1.0610E+00	1.6019E-05
	Rank	4	3	5	1	6	7	2
F9	Best	1.1231E+03	1.9125E+03	1.0402E+03	1.5009E+03	1.1487E+03	1.9119E+03	1.9619E+03
	Mean	1.7766E+03	2.0972E+03	1.3824E+03	1.9506E+03	1.8131E+03	2.2446E+03	2.3894E+03
	Std	3.3407E+02	1.0050E+02	1.4741E+02	2.2620E+02	2.5059E+02	1.6383E+02	1.7615E+02
	Rank	2	5	1	4	3	6	7

F10	Best	1.1000E+03	1.1017E+03	1.1032E+03	1.1023E+03	1.1020E+03	1.1053E+03	1.1037E+03
	Mean	1.1058E+03	1.1064E+03	1.1106E+03	1.1058E+03	1.1069E+03	1.1127E+03	1.1062E+03
	Std	4.9329E+00	2.9064E+00	5.0091E+00	1.6813E+00	2.5352E+00	3.0254E+00	1.3390E+00
	Rank	1	4	6	2	5	7	3
F11	Best	1.2000E+03	2.1599E+04	6.6885E+03	1.7078E+03	2.9097E+04	3.3775E+03	1.2256E+03
	Mean	1.2664E+03	3.0129E+05	4.7096E+04	1.1038E+04	1.3879E+05	2.6483E+04	1.4389E+03
	Std	9.3930E+01	3.6239E+05	3.5952E+04	1.2391E+04	1.4650E+05	1.2323E+04	1.0064E+02
	Rank	1	7	5	3	6	4	2
F12	Best	1.3003E+03	1.3689E+03	1.3298E+03	1.3109E+03	1.4814E+03	1.3434E+03	1.3058E+03
	Mean	1.3039E+03	5.0469E+03	1.9937E+03	1.5837E+03	2.1828E+03	1.3871E+03	1.3123E+03
	Std	3.3659E+00	5.6742E+03	6.9646E+02	1.0410E+03	4.9776E+02	2.5605E+01	3.3240E+00
	Rank	1	7	5	4	6	3	2
F13	Best	1.4000E+03	1.4458E+03	1.4222E+03	1.4033E+03	1.4366E+03	1.4163E+03	1.4120E+03
	Mean	1.4031E+03	1.5549E+03	1.4401E+03	1.4291E+03	1.4585E+03	1.4273E+03	1.4231E+03
	Std	5.7833E+00	2.2567E+02	1.2243E+01	1.1620E+01	1.2233E+01	3.1443E+00	4.2495E+00
	Rank	1	7	5	4	6	3	2
F14	Best	1.5000E+03	1.5358E+03	1.5080E+03	1.5023E+03	1.5417E+03	1.5026E+03	1.5023E+03
	Mean	1.5014E+03	1.7364E+03	1.5343E+03	1.5177E+03	1.6086E+03	1.5109E+03	1.5038E+03
	Std	5.7709E+00	2.3632E+02	2.2881E+01	8.8837E+00	4.7016E+01	3.5207E+00	8.5205E-01
	Rank	1	7	5	4	6	3	2
F15	Best	1.6000E+03	1.6036E+03	1.6019E+03	1.6027E+03	1.6045E+03	1.6102E+03	1.6058E+03
	Mean	1.7623E+03	1.6352E+03	1.6200E+03	1.6371E+03	1.6196E+03	1.6490E+03	1.6257E+03
	Std	1.0048E+02	3.4732E+01	1.5310E+01	4.7808E+01	2.6964E+01	2.0007E+01	1.2732E+01
	Rank	7	4	2	5	1	6	3
F16	Best	1.7024E+03	1.7171E+03	1.7017E+03	1.7061E+03	1.7267E+03	1.7373E+03	1.7382E+03
	Mean	1.7208E+03	1.7369E+03	1.7170E+03	1.7492E+03	1.7397E+03	1.7555E+03	1.7543E+03
	Std	2.7687E+01	1.0117E+01	1.0854E+01	3.9521E+01	1.0015E+01	9.7489E+00	9.9326E+00
	Rank	2	3	1	5	4	7	6
F17	Best	1.8000E+03	4.1404E+03	1.9176E+03	1.8070E+03	2.4002E+03	1.8323E+03	1.8087E+03
	Mean	1.8032E+03	8.6641E+03	5.0711E+03	3.5999E+03	5.1415E+03	1.8676E+03	1.8174E+03
	Std	8.4084E+00	4.6488E+03	2.7717E+03	6.2444E+03	2.0079E+03	2.3293E+01	5.3867E+00
	Rank	1	7	5	4	6	3	2
F18	Best	1.9000E+03	1.9208E+03	1.9026E+03	1.9016E+03	1.9172E+03	1.9041E+03	1.9020E+03
	Mean	1.9015E+03	1.9758E+03	1.9284E+03	1.9075E+03	1.9553E+03	1.9071E+03	1.9037E+03
	Std	6.7051E+00	6.8381E+01	3.7243E+01	2.2867E+00	2.7474E+01	1.2184E+00	9.8119E-01
	Rank	1	7	5	4	6	3	2
F19	Best	2.0003E+03	2.0243E+03	2.0015E+03	2.0181E+03	2.0204E+03	2.0356E+03	2.0157E+03
	Mean	2.0478E+03	2.0436E+03	2.0077E+03	2.0319E+03	2.0312E+03	2.0512E+03	2.0424E+03
	Std	6.2270E+01	9.9469E+00	6.9248E+00	7.6930E+00	9.3423E+00	8.6312E+00	8.8439E+00
	Rank	6	5	1	3	2	7	4
F20	Best	2.1000E+03	2.2026E+03	2.2001E+03	2.1790E+03	2.2006E+03	2.2023E+03	2.2000E+03
	Mean	2.2855E+03	2.2457E+03	2.2253E+03	2.2471E+03	2.2257E+03	2.2545E+03	2.2741E+03
	Std	5.9326E+01	5.5662E+01	4.5777E+01	6.1522E+01	4.5206E+01	5.6767E+01	6.0823E+01
	Rank	7	3	1	4	2	5	6
F21	Best	2.3003E+03	2.2197E+03	2.2154E+03	2.2116E+03	2.2152E+03	2.2229E+03	2.2116E+03
	Mean	2.3919E+03	2.3221E+03	2.2962E+03	2.2973E+03	2.2915E+03	2.2973E+03	2.2980E+03
	Std	2.9812E+02	1.4412E+02	2.5584E+01	1.6190E+01	2.6826E+01	2.6329E+01	1.6369E+01
	Rank	7	6	2	3	1	4	5
F22	Best	2.6055E+03	2.6104E+03	2.6068E+03	2.6108E+03	2.6060E+03	2.6143E+03	2.6089E+03

	Mean	2.6162E+03	2.6208E+03	2.6166E+03	2.6213E+03	2.6130E+03	2.6252E+03	2.6186E+03
	Std	6.3927E+00	6.0258E+00	4.7794E+00	4.7039E+00	4.0027E+00	4.5240E+00	5.4650E+00
	Rank	2	5	3	6	1	7	4
F23	Best	2.5000E+03	2.5092E+03	2.5003E+03	2.5000E+03	2.5052E+03	2.5213E+03	2.5003E+03
	Mean	2.7365E+03	2.7101E+03	2.6856E+03	2.7290E+03	2.6536E+03	2.7116E+03	2.7429E+03
	Std	4.5287E+01	8.7104E+01	1.0705E+02	7.6717E+01	1.0675E+02	8.3850E+01	4.5988E+01
	Rank	6	3	2	5	1	4	7
F24	Best	2.6000E+03	2.6367E+03	2.8978E+03	2.8977E+03	2.8979E+03	2.8989E+03	2.8977E+03
	Mean	2.9062E+03	2.9050E+03	2.9221E+03	2.9196E+03	2.9160E+03	2.9193E+03	2.9166E+03
	Std	6.2537E+01	5.5194E+01	2.3685E+01	2.3327E+01	2.0615E+01	2.0713E+01	2.3117E+01
	Rank	2	1	7	6	3	5	4
F25	Best	2.8000E+03	2.6019E+03	2.9002E+03	2.9000E+03	2.8038E+03	2.9018E+03	2.9000E+03
	Mean	3.3659E+03	3.0345E+03	2.9128E+03	2.9016E+03	2.8998E+03	2.9057E+03	2.9000E+03
	Std	5.4672E+02	3.8362E+02	2.0694E+01	8.6081E+00	2.0018E+01	2.9491E+00	9.0717E-04
	Rank	7	6	5	3	1	4	2
F26	Best	3.0889E+03	3.0891E+03	3.0890E+03	3.0890E+03	3.0895E+03	3.0945E+03	3.0890E+03
	Mean	3.1003E+03	3.0943E+03	3.0903E+03	3.0895E+03	3.0908E+03	3.0971E+03	3.0894E+03
	Std	1.7097E+01	2.8280E+00	1.0707E+00	3.1071E-01	1.6475E+00	1.8498E+00	2.2176E-01
	Rank	7	5	3	2	4	6	1
F27	Best	3.1000E+03	3.1000E+03	3.1036E+03	3.1000E+03	3.1007E+03	3.1104E+03	3.1000E+03
	Mean	3.2052E+03	3.1300E+03	3.1649E+03	3.1651E+03	3.1532E+03	3.1494E+03	3.2316E+03
	Std	1.3135E+02	3.3936E+01	2.3416E+01	1.2275E+02	2.3364E+01	2.4446E+01	1.5283E+02
	Rank	6	1	4	5	3	2	7
F28	Best	3.1293E+03	3.1543E+03	3.1354E+03	3.1483E+03	3.1411E+03	3.1828E+03	3.1613E+03
	Mean	3.1842E+03	3.1863E+03	3.1575E+03	3.1810E+03	3.1731E+03	3.2067E+03	3.1876E+03
	Std	6.6041E+01	1.6812E+01	1.2035E+01	2.0323E+01	1.8016E+01	1.7703E+01	1.4625E+01
	Rank	4	5	1	3	2	7	6
F29	Best	3.3945E+03	1.3839E+04	3.8971E+03	3.4288E+03	4.8084E+03	6.4675E+03	3.3982E+03
	Mean	3.0644E+04	5.7709E+04	7.5748E+04	1.2472E+05	3.8981E+04	5.6473E+04	8.8251E+04
	Std	1.4920E+05	4.5589E+04	2.0520E+05	2.7981E+05	6.1430E+04	1.0723E+05	2.4886E+05
	Rank	1	4	5	7	2	3	6

Table S10. The statistics results of MSEDO and improved algorithms solving CEC2017 (D=30)

No.	Index	MSEDO	EDO	IRIME	EMTLBO	EOSMA	MTVSCA	APSM-jSO
F1	Best	1.0003E+02	3.0527E+02	1.9455E+06	1.0952E+02	1.7380E+06	9.5207E+06	1.0143E+02
	Mean	1.0148E+02	3.0404E+03	6.3471E+06	4.1194E+03	5.0245E+06	2.0581E+07	2.4479E+03
	Std	4.1493E-01	3.6727E+03	3.6436E+06	3.9376E+03	2.6085E+06	7.9304E+06	1.5906E+03
	Rank	1	3	6	4	5	7	2
F2	Best	3.0000E+02	6.0706E+04	2.3698E+04	1.4028E+03	2.2890E+04	7.5232E+03	3.0057E+02
	Mean	3.0000E+02	8.5772E+04	4.0841E+04	1.1092E+04	3.7035E+04	1.5136E+04	3.0813E+02
	Std	7.2411E-07	1.3340E+04	1.0114E+04	9.7301E+03	8.1396E+03	3.7600E+03	6.9358E+00
	Rank	1	7	6	3	5	4	2
F3	Best	4.0000E+02	4.0424E+02	4.8891E+02	4.0424E+02	4.9319E+02	4.8796E+02	4.8434E+02
	Mean	4.2235E+02	4.9474E+02	5.1259E+02	4.8559E+02	5.1740E+02	5.3340E+02	4.8730E+02
	Std	2.9431E+01	3.1250E+01	1.8560E+01	2.9083E+01	1.4607E+01	1.7076E+01	5.1130E+00
	Rank	1	4	5	2	6	7	3
F4	Best	5.4079E+02	6.1971E+02	5.4083E+02	6.3977E+02	5.5098E+02	6.5008E+02	6.3345E+02
	Mean	5.8872E+02	6.7574E+02	5.6957E+02	6.6974E+02	5.8658E+02	6.8320E+02	6.5463E+02
	Std	3.2737E+01	2.0990E+01	1.7542E+01	1.1531E+01	1.3620E+01	1.0551E+01	1.2133E+01

	Rank	3	6	1	5	2	7	4
F5	Best	6.0000E+02	6.0068E+02	6.0078E+02	6.0003E+02	6.0111E+02	6.0328E+02	6.0001E+02
	Mean	6.0197E+02	6.0480E+02	6.0126E+02	6.0007E+02	6.0186E+02	6.0459E+02	6.0001E+02
	Std	2.5760E+00	7.2877E+00	3.4371E-01	3.7826E-02	7.4058E-01	8.0502E-01	4.5501E-03
	Rank	5	7	3	2	4	6	1
F6	Best	7.8002E+02	8.7720E+02	7.8251E+02	8.7716E+02	8.0205E+02	8.8659E+02	8.5804E+02
	Mean	8.4944E+02	9.1222E+02	8.2704E+02	9.0226E+02	8.4139E+02	9.2709E+02	8.8789E+02
	Std	3.3425E+01	1.9114E+01	2.2101E+01	1.2076E+01	1.8087E+01	1.4803E+01	1.3220E+01
	Rank	3	6	1	5	2	7	4
F7	Best	8.4776E+02	9.2645E+02	8.4231E+02	9.3912E+02	8.5116E+02	9.5222E+02	9.3104E+02
	Mean	8.9299E+02	9.7066E+02	8.7485E+02	9.6702E+02	8.8327E+02	9.8230E+02	9.5325E+02
	Std	2.9080E+01	2.2237E+01	2.1819E+01	1.0947E+01	1.9065E+01	1.1001E+01	1.1723E+01
	Rank	3	6	1	5	2	7	4
F8	Best	9.0000E+02	9.3694E+02	9.1967E+02	9.0000E+02	9.1336E+02	9.3014E+02	9.0000E+02
	Mean	1.0213E+03	1.7221E+03	9.9891E+02	9.0265E+02	9.5501E+02	9.7137E+02	9.0004E+02
	Std	4.5338E+02	7.2002E+02	7.4287E+01	3.2105E+00	4.4261E+01	2.8755E+01	5.0404E-02
	Rank	6	7	5	2	3	4	1
F9	Best	3.0658E+03	5.8261E+03	3.2316E+03	6.2615E+03	4.8739E+03	7.4047E+03	7.5919E+03
	Mean	6.6245E+03	6.4926E+03	4.4398E+03	7.5710E+03	6.5564E+03	7.8879E+03	8.1556E+03
	Std	1.5034E+03	2.9803E+02	5.5132E+02	3.8593E+02	5.6659E+02	2.6352E+02	3.2028E+02
	Rank	4	2	1	5	3	6	7
F10	Best	1.1050E+03	1.2366E+03	1.1806E+03	1.1424E+03	1.2050E+03	1.1982E+03	1.1192E+03
	Mean	1.1324E+03	1.3140E+03	1.2459E+03	1.1921E+03	1.2921E+03	1.2565E+03	1.1677E+03
	Std	2.5799E+01	5.4545E+01	3.2330E+01	3.0624E+01	4.4486E+01	3.1790E+01	3.2513E+01
	Rank	1	7	4	3	6	5	2
F11	Best	1.2134E+03	5.9990E+04	3.0297E+05	1.5975E+04	2.5821E+05	5.6596E+05	3.0061E+03
	Mean	5.9259E+03	4.3781E+05	4.1116E+06	9.2663E+04	1.3955E+06	2.0903E+06	9.4382E+03
	Std	8.2464E+03	3.6357E+05	2.6111E+06	1.0606E+05	1.4664E+06	1.1189E+06	5.0677E+03
	Rank	1	4	7	3	5	6	2
F12	Best	1.3445E+03	1.5918E+03	7.9955E+03	1.7602E+03	7.4052E+03	1.7265E+04	1.4231E+03
	Mean	1.1104E+04	3.6597E+04	4.3830E+04	8.4102E+03	4.5983E+04	4.5274E+04	1.4954E+03
	Std	1.9693E+04	1.8302E+04	3.5036E+04	1.0547E+04	3.0503E+04	1.9648E+04	5.3014E+01
	Rank	3	4	5	2	7	6	1
F13	Best	1.4363E+03	7.3693E+03	6.0297E+03	1.4906E+03	1.9949E+03	1.5419E+03	1.4601E+03
	Mean	1.4503E+03	2.9967E+04	1.9488E+04	4.5286E+03	5.4228E+03	1.5856E+03	1.4724E+03
	Std	8.7641E+00	2.3039E+04	1.2879E+04	5.1341E+03	2.7080E+03	2.8309E+01	6.8738E+00
	Rank	1	7	6	4	5	3	2
F14	Best	1.5165E+03	1.5789E+03	2.8578E+03	1.5896E+03	8.0077E+03	2.8950E+03	1.5388E+03
	Mean	2.8983E+03	1.7367E+04	1.1768E+04	5.9544E+03	2.7399E+04	5.0899E+03	1.5753E+03
	Std	5.5076E+03	1.0025E+04	1.0729E+04	8.7044E+03	2.2370E+04	1.5369E+03	2.6464E+01
	Rank	2	6	5	4	7	3	1
F15	Best	1.8149E+03	2.6610E+03	1.9113E+03	1.9090E+03	1.7665E+03	2.7049E+03	2.2697E+03
	Mean	2.3016E+03	3.1266E+03	2.4887E+03	2.9275E+03	2.3936E+03	3.0278E+03	2.8859E+03
	Std	3.3898E+02	1.8984E+02	2.3939E+02	3.3903E+02	2.7649E+02	1.6030E+02	2.2574E+02
	Rank	1	7	3	5	2	6	4
F16	Best	1.7517E+03	1.8166E+03	1.7470E+03	1.7852E+03	1.8059E+03	2.0447E+03	1.9217E+03
	Mean	2.0114E+03	2.1527E+03	2.0001E+03	2.0407E+03	1.9008E+03	2.1877E+03	2.0371E+03
	Std	1.6424E+02	1.7370E+02	1.5811E+02	1.5381E+02	8.4186E+01	8.4872E+01	7.8300E+01
	Rank	3	6	2	5	1	7	4

F17	Best	1.8264E+03	1.2573E+05	2.3130E+04	2.3112E+03	7.4623E+04	7.5453E+03	1.8433E+03
	Mean	1.8318E+03	5.7885E+05	4.4146E+05	7.6494E+04	1.8756E+05	1.8118E+04	1.8877E+03
	Std	1.4891E+01	4.2539E+05	4.6137E+05	1.0797E+05	9.8239E+04	7.7314E+03	3.2378E+01
	Rank	1	7	6	4	5	3	2
F18	Best	1.9173E+03	1.9786E+03	2.2270E+03	1.9399E+03	2.1793E+03	2.3043E+03	1.9308E+03
	Mean	2.6267E+03	2.0098E+04	1.3384E+04	2.6312E+03	1.2191E+04	4.4668E+03	1.9396E+03
	Std	2.5069E+03	1.6027E+04	1.3687E+04	1.5789E+03	1.1325E+04	2.7726E+03	5.9716E+00
	Rank	2	7	6	3	5	4	1
F19	Best	2.0526E+03	2.4584E+03	2.0651E+03	2.1955E+03	2.1119E+03	2.4077E+03	2.2883E+03
	Mean	2.4441E+03	2.7232E+03	2.2838E+03	2.5854E+03	2.2808E+03	2.6207E+03	2.4812E+03
	Std	2.5198E+02	1.1965E+02	1.5109E+02	1.3582E+02	9.2912E+01	9.6627E+01	9.7705E+01
	Rank	3	7	2	5	1	6	4
F20	Best	2.3397E+03	2.4326E+03	2.3493E+03	2.4313E+03	2.3483E+03	2.4587E+03	2.4204E+03
	Mean	2.3797E+03	2.4700E+03	2.3742E+03	2.4569E+03	2.3865E+03	2.4761E+03	2.4425E+03
	Std	3.1585E+01	2.2692E+01	1.6101E+01	1.1969E+01	1.7716E+01	1.0240E+01	1.4725E+01
	Rank	2	6	1	5	3	7	4
F21	Best	2.3000E+03	7.1523E+03	2.3148E+03	2.3000E+03	2.3125E+03	2.3197E+03	2.3000E+03
	Mean	5.5608E+03	7.7073E+03	2.7648E+03	2.5235E+03	2.3164E+03	2.3389E+03	2.3000E+03
	Std	2.7843E+03	3.0644E+02	1.1518E+03	1.2214E+03	3.4893E+00	2.1177E+01	2.5734E-03
	Rank	6	7	5	4	2	3	1
F22	Best	2.6921E+03	2.7734E+03	2.7017E+03	2.7287E+03	2.6854E+03	2.8085E+03	2.7635E+03
	Mean	2.7318E+03	2.8320E+03	2.7283E+03	2.8017E+03	2.7287E+03	2.8381E+03	2.7962E+03
	Std	3.0304E+01	2.5066E+01	1.6120E+01	1.9850E+01	2.2084E+01	1.1507E+01	1.3198E+01
	Rank	3	6	1	5	2	7	4
F23	Best	2.8464E+03	2.9838E+03	2.8636E+03	2.9539E+03	2.8534E+03	2.9681E+03	2.9366E+03
	Mean	2.9011E+03	3.0101E+03	2.8968E+03	2.9853E+03	2.8854E+03	3.0039E+03	2.9622E+03
	Std	3.6066E+01	1.8030E+01	2.5376E+01	1.3924E+01	1.8620E+01	1.7794E+01	1.0621E+01
	Rank	3	7	2	5	1	6	4
F24	Best	2.8834E+03	2.8838E+03	2.8847E+03	2.8835E+03	2.8902E+03	2.8970E+03	2.8867E+03
	Mean	2.8872E+03	2.8879E+03	2.9006E+03	2.8890E+03	2.9165E+03	2.9111E+03	2.8868E+03
	Std	2.1901E+00	2.1579E+00	1.5937E+01	1.0052E+01	1.8144E+01	1.1491E+01	8.4512E-02
	Rank	2	3	5	4	7	6	1
F25	Best	4.0280E+03	5.1156E+03	4.1471E+03	2.8002E+03	2.8620E+03	2.9905E+03	4.4528E+03
	Mean	4.3714E+03	5.5703E+03	4.4222E+03	4.6266E+03	4.0574E+03	5.2752E+03	4.8500E+03
	Std	2.2448E+02	2.0210E+02	1.6268E+02	8.5764E+02	5.2247E+02	5.9747E+02	1.7015E+02
	Rank	2	7	3	4	1	6	5
F26	Best	3.1742E+03	3.2132E+03	3.2023E+03	3.1726E+03	3.2091E+03	3.2404E+03	3.1895E+03
	Mean	3.2206E+03	3.2391E+03	3.2137E+03	3.2051E+03	3.2243E+03	3.2628E+03	3.2035E+03
	Std	1.8197E+01	1.3575E+01	5.5233E+00	1.3068E+01	9.8919E+00	1.2719E+01	7.3106E+00
	Rank	4	6	3	2	5	7	1
F27	Best	3.1000E+03	3.1902E+03	3.2268E+03	3.1897E+03	3.2167E+03	3.2360E+03	3.1801E+03
	Mean	3.1318E+03	3.2197E+03	3.2731E+03	3.2114E+03	3.2554E+03	3.2760E+03	3.2106E+03
	Std	5.4952E+01	1.6162E+01	4.1133E+01	1.5649E+01	2.6295E+01	2.0956E+01	1.5404E+01
	Rank	1	4	6	3	5	7	2
F28	Best	3.2642E+03	3.6779E+03	3.3473E+03	3.4942E+03	3.5229E+03	3.8489E+03	3.6202E+03
	Mean	3.4428E+03	3.8739E+03	3.6013E+03	3.7913E+03	3.7050E+03	4.0933E+03	3.7689E+03
	Std	1.2868E+02	1.2664E+02	1.6099E+02	1.7508E+02	1.2254E+02	1.3490E+02	9.3808E+01
	Rank	1	6	2	5	3	7	4
F29	Best	4.9516E+03	1.1870E+04	1.4755E+04	5.6042E+03	5.7801E+04	2.9880E+04	5.1123E+03

	Mean	5.0560E+03	2.7493E+04	4.0645E+04	1.1587E+04	2.9813E+05	8.9644E+04	5.5411E+03
	Std	6.7568E+01	1.3012E+04	2.7513E+04	5.6530E+03	2.5836E+05	3.3241E+04	3.1146E+02
	Rank	1	4	5	3	7	6	2

Table S11. The statistics results of MSEDO and improved algorithms solving CEC2017 (D=50)

No.	Index	MSEDO	EDO	IRIME	EMTLBO	EOSMA	MTVSCA	APSM-jSO
F1	Best	1.0013E+02	4.0728E+03	1.9929E+07	1.1865E+03	3.4077E+07	9.4498E+07	1.2004E+02
	Mean	3.2584E+03	6.0924E+04	4.6944E+07	6.3506E+03	7.3289E+07	1.9989E+08	2.3612E+03
	Std	3.6554E+03	7.3973E+04	1.5287E+07	5.8938E+03	2.8295E+07	7.5531E+07	2.1902E+03
	Rank	2	4	5	3	6	7	1
F2	Best	3.0000E+02	1.4211E+05	8.0547E+04	1.4572E+04	7.2343E+04	3.1296E+04	5.7612E+02
	Mean	3.0000E+02	1.9863E+05	1.3524E+05	3.6104E+04	9.7762E+04	5.2371E+04	1.5275E+03
	Std	1.5309E-06	2.7624E+04	2.7300E+04	1.5530E+04	1.2763E+04	9.8659E+03	6.8718E+02
	Rank	1	7	6	3	5	4	2
F3	Best	4.0000E+02	4.3639E+02	4.9994E+02	4.6901E+02	5.5993E+02	5.9580E+02	4.3961E+02
	Mean	4.2879E+02	5.4903E+02	6.1934E+02	5.5832E+02	6.4396E+02	6.8608E+02	5.3068E+02
	Std	4.2571E+01	4.4080E+01	4.7821E+01	4.8896E+01	4.5426E+01	3.6904E+01	4.6253E+01
	Rank	1	3	5	4	6	7	2
F4	Best	5.9950E+02	8.0275E+02	5.9651E+02	8.3843E+02	6.0478E+02	8.3929E+02	7.5731E+02
	Mean	6.9071E+02	8.8399E+02	6.5671E+02	8.6159E+02	6.8570E+02	8.7158E+02	8.0446E+02
	Std	7.7283E+01	3.0885E+01	2.7314E+01	1.2855E+01	3.3241E+01	1.1646E+01	1.4387E+01
	Rank	3	7	1	5	2	6	4
F5	Best	6.0006E+02	6.0399E+02	6.0178E+02	6.0019E+02	6.0229E+02	6.0485E+02	6.0001E+02
	Mean	6.0427E+02	6.2258E+02	6.0308E+02	6.0033E+02	6.0519E+02	6.0745E+02	6.0004E+02
	Std	4.0040E+00	1.7343E+01	5.9598E-01	1.3674E-01	1.1794E+00	1.2568E+00	2.3045E-02
	Rank	4	7	3	2	5	6	1
F6	Best	8.6227E+02	1.1062E+03	9.1133E+02	1.0490E+03	9.1785E+02	1.0992E+03	1.0210E+03
	Mean	1.0693E+03	1.1951E+03	9.8933E+02	1.1086E+03	9.9490E+02	1.1454E+03	1.0532E+03
	Std	9.0640E+01	4.8507E+01	3.8783E+01	2.0819E+01	3.6740E+01	2.2936E+01	1.4845E+01
	Rank	4	7	1	5	2	6	3
F7	Best	8.8557E+02	1.0822E+03	9.1288E+02	1.1285E+03	9.3901E+02	1.1251E+03	1.0684E+03
	Mean	9.7726E+02	1.1621E+03	9.6598E+02	1.1535E+03	9.9201E+02	1.1685E+03	1.1002E+03
	Std	7.2787E+01	3.1966E+01	3.2391E+01	1.4161E+01	3.7618E+01	1.7050E+01	1.6027E+01
	Rank	2	6	1	5	3	7	4
F8	Best	9.0045E+02	2.4541E+03	1.1130E+03	9.0348E+02	1.1075E+03	1.2851E+03	9.0000E+02
	Mean	1.8929E+03	9.6788E+03	1.7773E+03	9.1611E+02	1.5384E+03	1.5978E+03	9.0079E+02
	Std	1.7664E+03	3.8539E+03	4.6776E+02	1.1670E+01	3.0399E+02	2.4255E+02	5.9370E-01
	Rank	6	7	5	2	3	4	1
F9	Best	1.0398E+04	1.0142E+04	6.1121E+03	1.0044E+04	1.0020E+04	1.3006E+04	1.3312E+04
	Mean	1.1151E+04	1.1138E+04	7.6974E+03	1.3410E+04	1.1677E+04	1.3987E+04	1.4165E+04
	Std	3.1728E+02	5.6569E+02	7.7645E+02	7.2513E+02	8.7228E+02	4.1155E+02	3.9165E+02
	Rank	3	2	1	5	4	6	7
F10	Best	1.1352E+03	2.3384E+03	1.3209E+03	1.2617E+03	1.4898E+03	1.4454E+03	1.1582E+03
	Mean	1.2296E+03	3.4394E+03	1.4959E+03	1.3200E+03	1.6589E+03	1.5203E+03	1.1921E+03
	Std	3.3379E+01	6.5475E+02	8.9650E+01	3.9489E+01	1.1864E+02	5.2312E+01	2.7810E+01
	Rank	2	7	4	3	6	5	1
F11	Best	4.4898E+03	1.9074E+06	4.5701E+06	9.1654E+04	3.0669E+06	7.3367E+06	2.0120E+04
	Mean	1.1372E+04	4.8349E+06	3.8998E+07	1.0422E+06	1.5294E+07	2.0338E+07	2.0513E+05
	Std	1.2310E+04	2.0424E+06	1.9458E+07	7.9235E+05	9.6516E+06	8.4642E+06	1.6267E+05

	Rank	1	4	7	3	5	6	2
F12	Best	1.4853E+03	1.7861E+03	4.5630E+04	1.6597E+03	2.7483E+04	5.3425E+04	2.4362E+03
	Mean	6.0416E+03	7.1538E+03	1.1080E+05	7.4310E+03	7.2014E+04	1.6265E+05	3.0472E+03
	Std	4.9066E+03	6.1075E+03	5.2680E+04	6.0699E+03	3.7010E+04	1.1017E+05	4.0682E+02
	Rank	2	3	6	4	5	7	1
F13	Best	1.4788E+03	2.2616E+05	2.5519E+04	1.6959E+03	1.0697E+04	1.8421E+03	1.5235E+03
	Mean	1.5040E+03	4.8320E+05	1.8345E+05	2.0144E+04	5.5242E+04	2.5282E+03	1.5581E+03
	Std	8.0550E+00	1.7139E+05	1.1749E+05	2.8198E+04	3.2210E+04	1.1347E+03	1.6320E+01
	Rank	1	7	6	4	5	3	2
F14	Best	1.6136E+03	1.7445E+03	9.2481E+03	1.6900E+03	5.8809E+03	8.3216E+03	1.7119E+03
	Mean	3.2448E+03	1.5307E+04	2.4886E+04	7.6466E+03	1.7502E+04	1.8225E+04	1.8295E+03
	Std	2.3423E+03	8.1859E+03	1.0524E+04	6.6363E+03	7.9513E+03	6.0351E+03	5.9797E+01
	Rank	2	4	7	3	5	6	1
F15	Best	2.1484E+03	4.0166E+03	2.3984E+03	2.2550E+03	2.0987E+03	3.8465E+03	3.3840E+03
	Mean	2.8262E+03	4.5182E+03	3.2774E+03	4.0711E+03	3.1023E+03	4.4559E+03	4.0132E+03
	Std	4.8980E+02	2.5510E+02	4.1325E+02	4.2804E+02	4.3126E+02	2.6405E+02	2.9082E+02
	Rank	1	7	3	5	2	6	4
F16	Best	2.1500E+03	2.4026E+03	2.3399E+03	2.4751E+03	2.1647E+03	3.0585E+03	2.8711E+03
	Mean	2.8850E+03	3.3866E+03	2.8427E+03	3.1535E+03	2.6824E+03	3.5709E+03	3.2575E+03
	Std	5.0863E+02	2.9227E+02	2.9510E+02	4.2689E+02	2.7990E+02	1.6519E+02	1.7410E+02
	Rank	3	6	2	4	1	7	5
F17	Best	1.8420E+03	5.5410E+05	2.7961E+05	1.5201E+04	9.8933E+04	2.2027E+04	1.9273E+03
	Mean	1.8536E+03	2.1052E+06	1.5024E+06	2.4777E+05	6.3429E+05	6.0812E+04	2.1150E+03
	Std	7.8626E+00	1.4635E+06	1.2321E+06	2.4200E+05	3.5229E+05	1.8325E+04	9.6580E+01
	Rank	1	7	6	4	5	3	2
F18	Best	1.9272E+03	2.0132E+03	3.0036E+03	2.0394E+03	4.0573E+03	6.0730E+03	1.9426E+03
	Mean	2.3713E+03	8.1301E+03	1.6326E+04	5.0344E+03	1.4690E+04	1.9672E+04	1.9812E+03
	Std	1.2144E+03	1.0576E+04	1.2996E+04	5.3108E+03	8.1726E+03	9.1949E+03	2.0624E+01
	Rank	2	4	6	3	5	7	1
F19	Best	2.3849E+03	3.2019E+03	2.3970E+03	3.3621E+03	2.4583E+03	3.5347E+03	3.1523E+03
	Mean	3.0903E+03	3.6338E+03	2.8762E+03	3.6429E+03	2.8467E+03	3.7209E+03	3.5465E+03
	Std	3.1807E+02	1.7678E+02	2.7886E+02	1.1204E+02	1.8494E+02	9.6887E+01	1.9697E+02
	Rank	3	5	2	6	1	7	4
F20	Best	2.3784E+03	2.6312E+03	2.3797E+03	2.6048E+03	2.4064E+03	2.6064E+03	2.5692E+03
	Mean	2.4679E+03	2.6817E+03	2.4540E+03	2.6424E+03	2.4714E+03	2.6623E+03	2.6082E+03
	Std	6.6396E+01	3.6399E+01	2.8553E+01	1.4914E+01	2.6217E+01	1.8718E+01	1.3827E+01
	Rank	2	7	1	5	3	6	4
F21	Best	5.0645E+03	1.1947E+04	7.9953E+03	3.0214E+03	2.3478E+03	5.1411E+03	2.3036E+03
	Mean	1.2716E+04	1.3838E+04	9.2551E+03	1.3709E+04	1.0577E+04	1.4778E+04	1.4107E+04
	Std	1.4890E+03	5.1002E+02	7.8229E+02	3.3506E+03	4.6513E+03	2.5672E+03	4.0170E+03
	Rank	3	5	1	4	2	7	6
F22	Best	2.7843E+03	3.0764E+03	2.8464E+03	3.0130E+03	2.8515E+03	3.0700E+03	2.9915E+03
	Mean	2.8860E+03	3.1481E+03	2.9049E+03	3.0571E+03	2.9082E+03	3.1168E+03	3.0224E+03
	Std	5.7869E+01	3.1446E+01	2.9077E+01	2.3349E+01	2.9642E+01	1.9400E+01	1.1848E+01
	Rank	1	7	2	5	3	6	4
F23	Best	2.9958E+03	3.2799E+03	3.0124E+03	3.1574E+03	2.9867E+03	3.2346E+03	3.1578E+03
	Mean	3.0790E+03	3.3383E+03	3.0731E+03	3.2320E+03	3.0399E+03	3.2688E+03	3.1844E+03
	Std	8.1016E+01	3.4741E+01	3.9672E+01	2.1948E+01	2.7757E+01	2.0994E+01	1.3958E+01
	Rank	3	7	2	5	1	6	4

F24	Best	2.9287E+03	2.9882E+03	3.0697E+03	2.9908E+03	3.0794E+03	3.1145E+03	2.9815E+03
	Mean	3.0162E+03	3.0582E+03	3.1344E+03	3.0629E+03	3.1564E+03	3.1613E+03	3.0096E+03
	Std	4.1608E+01	2.5467E+01	3.9313E+01	2.9152E+01	4.4993E+01	2.6035E+01	2.8832E+01
	Rank	2	3	5	4	6	7	1
F25	Best	4.5769E+03	7.0641E+03	3.2119E+03	2.9141E+03	4.7029E+03	6.7932E+03	4.3535E+03
	Mean	5.5286E+03	7.7959E+03	5.3289E+03	5.9727E+03	5.3690E+03	7.4335E+03	6.2053E+03
	Std	4.8108E+02	3.6907E+02	5.7552E+02	1.0576E+03	3.8159E+02	2.3590E+02	4.4318E+02
	Rank	3	7	1	4	2	6	5
F26	Best	3.2347E+03	3.4426E+03	3.2805E+03	3.2367E+03	3.3346E+03	3.4472E+03	3.2037E+03
	Mean	3.3484E+03	3.7059E+03	3.3949E+03	3.3071E+03	3.4068E+03	3.5905E+03	3.2360E+03
	Std	8.4625E+01	1.6269E+02	7.7771E+01	6.8178E+01	4.2624E+01	7.0951E+01	1.7738E+01
	Rank	3	7	4	2	5	6	1
F27	Best	3.2534E+03	3.2659E+03	3.3048E+03	3.2632E+03	3.3725E+03	3.4025E+03	3.2591E+03
	Mean	3.2811E+03	3.3320E+03	3.3886E+03	3.3186E+03	3.4798E+03	3.5006E+03	3.2675E+03
	Std	2.2219E+01	2.8095E+01	3.5975E+01	2.7233E+01	6.6558E+01	6.6860E+01	1.6806E+01
	Rank	2	4	5	3	6	7	1
F28	Best	3.2421E+03	4.1706E+03	3.4347E+03	3.5558E+03	3.7442E+03	4.4608E+03	3.9731E+03
	Mean	3.7881E+03	4.7416E+03	3.9879E+03	4.3356E+03	4.1706E+03	5.1490E+03	4.2968E+03
	Std	2.9583E+02	4.1695E+02	2.8650E+02	5.0534E+02	2.6266E+02	2.2140E+02	1.6947E+02
	Rank	1	6	2	5	3	7	4
F29	Best	5.8888E+05	2.1555E+06	1.1284E+06	6.8549E+05	3.2597E+06	6.5816E+06	6.0194E+05
	Mean	6.7410E+05	3.2409E+06	1.7978E+06	1.0208E+06	7.3756E+06	1.3257E+07	7.3520E+05
	Std	1.0825E+05	8.0587E+05	3.9293E+05	2.3229E+05	3.1617E+06	2.9488E+06	1.0419E+05
	Rank	1	5	4	3	6	7	2

Table S12. The statistics results of MSEDO and improved algorithms solving CEC2017 (D=100)

No.	Index	MSEDO	EDO	IRIME	EMTLBO	EOSMA	MTVSCA	APSM-jSO
F1	Best	1.0913E+02	1.5586E+06	3.1256E+08	2.5725E+05	5.8682E+08	1.2723E+09	1.6661E+03
	Mean	5.5156E+03	1.0813E+07	6.8832E+08	1.0042E+06	1.3789E+09	2.0768E+09	7.3175E+03
	Std	5.6516E+03	1.0848E+07	1.9380E+08	5.7194E+05	4.6942E+08	4.0201E+08	4.7919E+03
	Rank	1	4	5	3	6	7	2
F2	Best	3.0000E+02	4.2148E+05	2.8337E+05	7.9135E+04	2.5109E+05	1.2806E+05	1.8131E+04
	Mean	3.0000E+02	5.2850E+05	3.3700E+05	1.5124E+05	3.0517E+05	1.5750E+05	2.6257E+04
	Std	2.8577E-04	5.6824E+04	3.6392E+04	4.4020E+04	3.1897E+04	1.9416E+04	4.9688E+03
	Rank	1	7	6	3	5	4	2
F3	Best	4.0000E+02	7.1766E+02	8.1094E+02	6.6176E+02	9.3832E+02	1.0226E+03	6.0017E+02
	Mean	4.3858E+02	8.0698E+02	1.0126E+03	7.5216E+02	1.1643E+03	1.1519E+03	6.3953E+02
	Std	6.1311E+01	4.7583E+01	8.8872E+01	5.3852E+01	1.1289E+02	6.9424E+01	3.3330E+01
	Rank	1	4	5	3	7	6	2
F4	Best	7.3083E+02	1.3953E+03	8.9376E+02	7.5771E+02	9.3486E+02	1.3908E+03	1.1399E+03
	Mean	9.9878E+02	1.4976E+03	9.9350E+02	1.3630E+03	1.0463E+03	1.4228E+03	1.2019E+03
	Std	1.8964E+02	5.4242E+01	5.2227E+01	1.2013E+02	5.6044E+01	2.2065E+01	2.8436E+01
	Rank	2	7	1	5	3	6	4
F5	Best	6.0185E+02	6.2317E+02	6.1026E+02	6.0146E+02	6.1045E+02	6.1205E+02	6.0010E+02
	Mean	6.1496E+02	6.4858E+02	6.1277E+02	6.0284E+02	6.1539E+02	6.1457E+02	6.0030E+02
	Std	8.5638E+00	2.0334E+01	1.7154E+00	8.4588E-01	2.8878E+00	1.5665E+00	1.1959E-01
	Rank	5	7	3	2	6	4	1
F6	Best	1.2853E+03	1.8401E+03	1.3449E+03	1.6294E+03	1.4820E+03	1.6883E+03	1.4933E+03
	Mean	1.7735E+03	2.0744E+03	1.5296E+03	1.7157E+03	1.5772E+03	1.8057E+03	1.5308E+03

	Std	2.7391E+02	1.2875E+02	8.4432E+01	3.3968E+01	9.0680E+01	4.4974E+01	1.8926E+01
	Rank	5	7	1	4	3	6	2
F7	Best	1.0388E+03	1.6774E+03	1.1821E+03	1.6148E+03	1.2298E+03	1.6400E+03	1.4500E+03
	Mean	1.3408E+03	1.8237E+03	1.2846E+03	1.6772E+03	1.3470E+03	1.7187E+03	1.5112E+03
	Std	2.4984E+02	7.4271E+01	5.6702E+01	3.6346E+01	5.5532E+01	4.2276E+01	2.3552E+01
	Rank	2	7	1	5	3	6	4
F8	Best	1.3888E+03	2.9244E+04	6.1615E+03	1.0442E+03	5.7756E+03	4.4994E+03	9.0452E+02
	Mean	1.0011E+04	4.7430E+04	1.2937E+04	1.4702E+03	1.2026E+04	7.1748E+03	9.1976E+02
	Std	8.1509E+03	8.8267E+03	3.8270E+03	7.5481E+02	3.6882E+03	1.7339E+03	8.2404E+00
	Rank	4	7	6	2	5	3	1
F9	Best	2.5350E+04	2.6317E+04	1.4537E+04	2.8202E+04	1.9969E+04	2.8674E+04	2.9031E+04
	Mean	2.7362E+04	2.7406E+04	1.7793E+04	2.9267E+04	2.4660E+04	3.0372E+04	3.0377E+04
	Std	6.8810E+02	5.2420E+02	1.6433E+03	4.3328E+02	2.0912E+03	5.8181E+02	5.5518E+02
	Rank	3	4	1	5	2	6	7
F10	Best	1.5587E+03	3.3086E+04	1.2695E+04	2.1461E+03	1.3628E+04	5.8003E+03	1.7692E+03
	Mean	1.7078E+03	7.2993E+04	2.1610E+04	4.0418E+03	2.2993E+04	7.8533E+03	2.0312E+03
	Std	1.0992E+02	1.6438E+04	6.5649E+03	2.7820E+03	5.5514E+03	1.2437E+03	1.4224E+02
	Rank	1	7	5	3	6	4	2
F11	Best	2.5064E+04	6.8305E+06	7.1841E+07	3.1922E+06	5.6125E+07	1.5580E+08	8.8006E+05
	Mean	5.3747E+04	3.1281E+07	2.8352E+08	8.5528E+06	1.7374E+08	2.4317E+08	1.9499E+06
	Std	2.0065E+04	1.6817E+07	1.0795E+08	5.1454E+06	8.6863E+07	7.3814E+07	8.6653E+05
	Rank	1	4	7	3	5	6	2
F12	Best	1.4692E+03	2.4953E+03	1.6318E+05	2.0426E+03	3.2045E+04	9.7697E+04	7.1548E+03
	Mean	6.8615E+03	5.1882E+03	4.4893E+05	6.0319E+03	6.4659E+04	1.8975E+05	9.8050E+03
	Std	5.0932E+03	2.8914E+03	2.2286E+05	3.4184E+03	2.0313E+04	9.6047E+04	1.8417E+03
	Rank	3	1	7	2	5	6	4
F13	Best	1.6446E+03	2.1961E+06	5.3366E+05	5.4432E+04	5.1454E+05	2.8857E+04	1.6484E+03
	Mean	1.6828E+03	5.4510E+06	2.0283E+06	2.9349E+05	1.1068E+06	7.5514E+04	1.7509E+03
	Std	1.5503E+01	2.2058E+06	1.2223E+06	2.4593E+05	3.4758E+05	3.7130E+04	6.2761E+01
	Rank	1	7	6	4	5	3	2
F14	Best	1.7211E+03	1.8750E+03	2.0781E+04	1.8209E+03	1.8311E+04	2.4936E+04	2.2031E+03
	Mean	4.6235E+03	4.8927E+03	7.2323E+04	4.4201E+03	3.4441E+04	3.9034E+04	2.4244E+03
	Std	4.0545E+03	3.9883E+03	3.5799E+04	2.6426E+03	1.2090E+04	9.3190E+03	1.3553E+02
	Rank	3	4	7	2	5	6	1
F15	Best	2.2939E+03	4.6297E+03	4.8043E+03	3.4921E+03	4.3559E+03	8.4630E+03	7.1336E+03
	Mean	4.9053E+03	8.7882E+03	5.8661E+03	7.5153E+03	5.6423E+03	9.1550E+03	8.0022E+03
	Std	1.8254E+03	1.0461E+03	5.9112E+02	1.8054E+03	7.7914E+02	2.5475E+02	3.9823E+02
	Rank	1	6	3	4	2	7	5
F16	Best	3.0024E+03	5.2287E+03	3.4842E+03	3.3730E+03	3.6098E+03	6.1536E+03	5.1965E+03
	Mean	5.0339E+03	6.3438E+03	4.6703E+03	4.8641E+03	4.5880E+03	6.5402E+03	5.9881E+03
	Std	1.0975E+03	4.0154E+02	4.7190E+02	1.2870E+03	5.0036E+02	2.0559E+02	3.1363E+02
	Rank	4	6	2	3	1	7	5
F17	Best	1.9330E+03	4.1028E+06	7.6028E+05	8.3418E+04	5.3713E+05	1.1501E+05	2.7133E+03
	Mean	1.9559E+03	1.4862E+07	2.6999E+06	4.0762E+05	1.5561E+06	1.8335E+05	4.6566E+03
	Std	1.2776E+01	6.4769E+06	1.1106E+06	2.4586E+05	7.1023E+05	5.3822E+04	1.4695E+03
	Rank	1	7	6	4	5	3	2
F18	Best	1.9946E+03	2.1666E+03	1.8775E+04	2.1244E+03	6.5657E+03	3.8061E+04	2.1138E+03
	Mean	8.6803E+03	7.5659E+03	9.2854E+04	6.2438E+03	2.0788E+04	1.0720E+05	2.1674E+03
	Std	6.6548E+03	6.3150E+03	7.0580E+04	4.6681E+03	1.1810E+04	5.4700E+04	4.4855E+01

	Rank	4	3	6	2	5	7	1
F19	Best	5.3994E+03	5.7532E+03	3.7542E+03	6.5481E+03	4.1010E+03	6.1592E+03	5.9127E+03
	Mean	6.4559E+03	6.4822E+03	4.6444E+03	6.9490E+03	4.8735E+03	6.9539E+03	6.7496E+03
	Std	3.3349E+02	4.1746E+02	5.0048E+02	2.0514E+02	4.6762E+02	2.5130E+02	2.8902E+02
	Rank	3	4	1	6	2	7	5
F20	Best	2.5762E+03	3.2391E+03	2.7025E+03	3.0793E+03	2.6997E+03	3.1686E+03	2.9762E+03
	Mean	2.8963E+03	3.3706E+03	2.8129E+03	3.1656E+03	2.8090E+03	3.2227E+03	3.0253E+03
	Std	2.4950E+02	6.9273E+01	6.1040E+01	3.8068E+01	5.4197E+01	2.6842E+01	2.4249E+01
	Rank	3	7	2	5	1	6	4
F21	Best	2.8692E+04	2.7228E+04	1.7344E+04	3.0436E+04	2.2720E+04	3.0886E+04	3.0700E+04
	Mean	2.9926E+04	2.8952E+04	2.0214E+04	3.1433E+04	2.6992E+04	3.2602E+04	3.2660E+04
	Std	4.8877E+02	6.7121E+02	1.3198E+03	5.5854E+02	1.7241E+03	6.9477E+02	8.2253E+02
	Rank	4	3	1	5	2	6	7
F22	Best	3.1244E+03	3.7857E+03	3.1517E+03	3.0063E+03	3.1791E+03	3.6939E+03	2.9614E+03
	Mean	3.2641E+03	3.9575E+03	3.2344E+03	3.0749E+03	3.2741E+03	3.7756E+03	3.2743E+03
	Std	8.5084E+01	6.2468E+01	4.5834E+01	8.8989E+01	5.3062E+01	3.6845E+01	2.1926E+02
	Rank	3	7	2	1	4	6	5
F23	Best	3.4807E+03	4.2957E+03	3.6305E+03	3.4732E+03	3.6157E+03	4.0976E+03	3.3181E+03
	Mean	3.7464E+03	4.4823E+03	3.7557E+03	3.6628E+03	3.7223E+03	4.2238E+03	3.4285E+03
	Std	1.4054E+02	8.6097E+01	6.3187E+01	1.7453E+02	5.8383E+01	4.7187E+01	1.5524E+02
	Rank	4	7	5	2	3	6	1
F24	Best	3.1372E+03	3.3339E+03	3.5509E+03	3.2919E+03	3.6424E+03	3.7689E+03	3.2276E+03
	Mean	3.2328E+03	3.4740E+03	3.7326E+03	3.4059E+03	3.8807E+03	3.9428E+03	3.3102E+03
	Std	5.4325E+01	6.6301E+01	7.2335E+01	5.3184E+01	1.0950E+02	1.0578E+02	3.0853E+01
	Rank	1	4	5	3	6	7	2
F25	Best	8.5685E+03	1.2147E+04	9.7962E+03	7.8622E+03	9.2636E+03	1.4046E+04	5.9295E+03
	Mean	1.0285E+04	1.7055E+04	1.0989E+04	9.2728E+03	1.0674E+04	1.5021E+04	6.7708E+03
	Std	1.3210E+03	1.1837E+03	5.1435E+02	1.6464E+03	8.3845E+02	4.4791E+02	9.5689E+02
	Rank	3	7	5	2	4	6	1
F26	Best	3.3863E+03	3.6509E+03	3.4574E+03	3.3565E+03	3.4338E+03	3.6824E+03	3.3170E+03
	Mean	3.4910E+03	3.8614E+03	3.5513E+03	3.4446E+03	3.6595E+03	3.8161E+03	3.3481E+03
	Std	7.8241E+01	1.3643E+02	6.6624E+01	5.2872E+01	6.7608E+01	8.1023E+01	2.0529E+01
	Rank	3	7	4	2	5	6	1
F27	Best	3.2620E+03	3.4953E+03	3.6230E+03	3.4610E+03	3.9161E+03	3.9751E+03	3.3383E+03
	Mean	3.3381E+03	3.6254E+03	3.7955E+03	3.5222E+03	4.1097E+03	4.3352E+03	3.4032E+03
	Std	4.9672E+01	7.6599E+01	1.0481E+02	4.4618E+01	1.5141E+02	2.0749E+02	3.3950E+01
	Rank	1	4	5	3	6	7	2
F28	Best	4.1747E+03	5.9941E+03	5.5900E+03	4.8547E+03	5.6151E+03	8.2019E+03	6.6339E+03
	Mean	5.7039E+03	8.4348E+03	6.5300E+03	7.2115E+03	6.6947E+03	9.1633E+03	7.4893E+03
	Std	7.2025E+02	1.1455E+03	4.7024E+02	1.4517E+03	5.6588E+02	3.6830E+02	4.1121E+02
	Rank	1	6	2	4	3	7	5
F29	Best	5.8090E+03	4.1378E+04	9.0424E+05	7.8260E+03	5.1366E+05	1.0810E+06	1.0999E+04
	Mean	4.3415E+04	1.8021E+05	2.2380E+06	1.3900E+04	1.7061E+06	2.4739E+06	1.7091E+04
	Std	1.6219E+05	1.3755E+05	9.9383E+05	4.5772E+03	5.7704E+05	8.0547E+05	3.5625E+03
	Rank	3	4	6	1	5	7	2

Table S13. The statistics results of MSEDO and improved algorithms solving CEC2022 (D=10)

No.	Index	MSEDO	EDO	IRIME	EMTLBO	EOSMA	MTVSCA	APSM-jSO
F1	Best	3.0000E+02	8.7876E+02	3.8362E+02	3.0162E+02	4.1176E+02	3.3211E+02	3.0006E+02

	Mean	3.0000E+02	2.2632E+03	8.6200E+02	6.8618E+02	7.2111E+02	3.9876E+02	3.0025E+02
	Std	4.3522E-14	8.3485E+02	3.8814E+02	7.9209E+02	1.7118E+02	6.0430E+01	1.8424E-01
	Rank	1	7	6	4	5	3	2
F2	Best	4.0000E+02	4.0000E+02	4.0521E+02	4.0005E+02	4.0014E+02	4.0091E+02	4.0017E+02
	Mean	4.0485E+02	4.0503E+02	4.0829E+02	4.0502E+02	4.0442E+02	4.0559E+02	4.0623E+02
	Std	1.2778E+01	3.3098E+00	9.7598E-01	3.7677E+00	3.9534E+00	3.2908E+00	2.5301E+00
	Rank	2	4	7	3	1	5	6
F3	Best	6.0000E+02	6.0000E+02	6.0014E+02	6.0019E+02	6.0010E+02	6.0050E+02	6.0018E+02
	Mean	6.0056E+02	6.0004E+02	6.0040E+02	6.0038E+02	6.0026E+02	6.0101E+02	6.0037E+02
	Std	1.4918E+00	5.9272E-02	1.9723E-01	1.2562E-01	1.6875E-01	2.7086E-01	1.1973E-01
	Rank	6	1	5	4	2	7	3
F4	Best	8.0298E+02	8.1318E+02	8.0717E+02	8.2090E+02	8.0684E+02	8.1302E+02	8.1661E+02
	Mean	8.1907E+02	8.2380E+02	8.1683E+02	8.3418E+02	8.1337E+02	8.3226E+02	8.3442E+02
	Std	1.1159E+01	5.6819E+00	5.7137E+00	5.0003E+00	4.4926E+00	5.6860E+00	6.4262E+00
	Rank	3	4	2	6	1	5	7
F5	Best	9.0000E+02	9.0000E+02	9.0016E+02	9.0001E+02	9.0001E+02	9.0057E+02	9.0001E+02
	Mean	9.0035E+02	9.0009E+02	9.0081E+02	9.0004E+02	9.0029E+02	9.0153E+02	9.0007E+02
	Std	1.1638E+00	2.0934E-01	8.4344E-01	3.7436E-02	2.9487E-01	9.5126E-01	5.3140E-02
	Rank	5	3	6	1	4	7	2
F6	Best	1.8000E+03	2.0045E+03	1.9255E+03	1.8180E+03	3.6860E+03	1.8672E+03	1.8084E+03
	Mean	1.8022E+03	4.6887E+03	6.5782E+03	2.7257E+03	1.1231E+04	1.9699E+03	1.8386E+03
	Std	6.3734E+00	1.8429E+03	4.4505E+03	1.7332E+03	7.7952E+03	9.4861E+01	2.4486E+01
	Rank	1	5	6	4	7	3	2
F7	Best	2.0000E+03	2.0144E+03	2.0037E+03	2.0265E+03	2.0079E+03	2.0177E+03	2.0271E+03
	Mean	2.0149E+03	2.0265E+03	2.0207E+03	2.0346E+03	2.0246E+03	2.0313E+03	2.0389E+03
	Std	2.2938E+01	3.8551E+00	4.0595E+00	4.1402E+00	6.2622E+00	4.3953E+00	4.1737E+00
	Rank	1	4	2	6	3	5	7
F8	Best	2.2017E+03	2.2113E+03	2.2044E+03	2.2135E+03	2.2104E+03	2.2207E+03	2.2213E+03
	Mean	2.2337E+03	2.2228E+03	2.2195E+03	2.2265E+03	2.2256E+03	2.2257E+03	2.2273E+03
	Std	4.2763E+01	3.3278E+00	6.0772E+00	3.9808E+00	4.1996E+00	2.1809E+00	2.6160E+00
	Rank	7	2	1	5	3	4	6
F9	Best	2.4000E+03	2.5293E+03	2.5293E+03	2.5293E+03	2.5293E+03	2.5301E+03	2.5293E+03
	Mean	2.5207E+03	2.5293E+03	2.5293E+03	2.5293E+03	2.5295E+03	2.5310E+03	2.5293E+03
	Std	3.2800E+01	2.5059E-02	4.2286E-03	3.5699E-03	1.8651E-01	6.7311E-01	1.7446E-03
	Rank	1	5	4	3	6	7	2
F10	Best	2.4070E+03	2.5003E+03	2.5003E+03	2.5003E+03	2.5003E+03	2.5004E+03	2.5003E+03
	Mean	2.5015E+03	2.5004E+03	2.5005E+03	2.5005E+03	2.5004E+03	2.5005E+03	2.5093E+03
	Std	2.7193E+01	5.7553E-02	8.8604E-02	7.0876E-02	8.5714E-02	8.6580E-02	3.3611E+01
	Rank	6	1	4	3	2	5	7
F11	Best	2.6000E+03	2.6001E+03	2.6381E+03	2.6019E+03	2.6165E+03	2.7176E+03	2.6066E+03
	Mean	2.8487E+03	2.8701E+03	2.7215E+03	2.6397E+03	2.7554E+03	2.8904E+03	2.8700E+03
	Std	1.1799E+02	9.1419E+01	3.6154E+01	5.9596E+01	1.2533E+02	1.1498E+02	1.0193E+02
	Rank	4	6	2	1	3	7	5
F12	Best	2.8639E+03	2.8602E+03	2.8587E+03	2.8608E+03	2.8628E+03	2.8648E+03	2.8613E+03
	Mean	2.8664E+03	2.8643E+03	2.8626E+03	2.8625E+03	2.8646E+03	2.8659E+03	2.8641E+03
	Std	2.0092E+00	1.2616E+00	1.3313E+00	8.4007E-01	7.5619E-01	6.4319E-01	1.0741E+00
	Rank	7	4	2	1	5	6	3

Table S14. The statistics results of MSEDO and improved algorithms solving CEC2022 (D=20)

No.	Index	MSEDO	EDO	IRIME	EMTLBO	EOSMA	MTVSCA	APSM-jSO
F1	Best	3.0000E+02	9.6051E+03	7.1132E+03	8.5904E+02	2.9015E+03	8.8042E+02	3.0089E+02
	Mean	3.0000E+02	2.1724E+04	1.6314E+04	4.5925E+03	7.5927E+03	2.5056E+03	3.0758E+02
	Std	9.0394E-08	5.6737E+03	5.1387E+03	3.0504E+03	2.4057E+03	7.7629E+02	9.6879E+00
	Rank	1	7	6	4	5	3	2
F2	Best	4.0000E+02	4.4490E+02	4.4520E+02	4.4911E+02	4.4976E+02	4.3665E+02	4.4493E+02
	Mean	4.3884E+02	4.5124E+02	4.5512E+02	4.5844E+02	4.6100E+02	4.5745E+02	4.4843E+02
	Std	1.9476E+01	7.4382E+00	9.4449E+00	1.1780E+01	1.0052E+01	8.3855E+00	1.5186E+00
	Rank	1	3	4	6	7	5	2
F3	Best	6.0000E+02	6.0010E+02	6.0069E+02	6.0056E+02	6.0059E+02	6.0165E+02	6.0021E+02
	Mean	6.0061E+02	6.0066E+02	6.0116E+02	6.0109E+02	6.0128E+02	6.0273E+02	6.0039E+02
	Std	1.5761E+00	7.1989E-01	3.1829E-01	3.4333E-01	4.8219E-01	7.2885E-01	9.3051E-02
	Rank	2	3	5	4	6	7	1
F4	Best	8.2089E+02	8.5664E+02	8.2479E+02	8.9142E+02	8.2821E+02	8.8373E+02	8.8466E+02
	Mean	8.4812E+02	8.8172E+02	8.5326E+02	9.0952E+02	8.4596E+02	9.0459E+02	9.0185E+02
	Std	1.8245E+01	1.2250E+01	1.4687E+01	9.3167E+00	8.7483E+00	9.3518E+00	9.2147E+00
	Rank	2	4	3	7	1	6	5
F5	Best	9.0000E+02	9.0056E+02	9.0654E+02	9.0020E+02	9.0092E+02	9.0335E+02	9.0004E+02
	Mean	9.4602E+02	9.2659E+02	9.3656E+02	9.0128E+02	9.0955E+02	9.1048E+02	9.0016E+02
	Std	1.5860E+02	6.5319E+01	3.0476E+01	1.0739E+00	7.4816E+00	5.5227E+00	6.8847E-02
	Rank	7	5	6	2	3	4	1
F6	Best	1.8002E+03	3.3424E+03	3.8557E+03	1.8460E+03	5.0444E+03	5.9699E+03	1.9976E+03
	Mean	1.8111E+03	1.5245E+04	2.5357E+04	5.1833E+03	4.4256E+04	3.1041E+04	2.3463E+03
	Std	2.2581E+01	1.2646E+04	2.1089E+04	3.8252E+03	6.1985E+04	1.7529E+04	2.6416E+02
	Rank	1	4	5	3	7	6	2
F7	Best	2.0149E+03	2.0463E+03	2.0269E+03	2.0880E+03	2.0343E+03	2.0636E+03	2.0774E+03
	Mean	2.0907E+03	2.0753E+03	2.0388E+03	2.1087E+03	2.0544E+03	2.0855E+03	2.0964E+03
	Std	8.7455E+01	1.4856E+01	8.6150E+00	1.1457E+01	9.1537E+00	8.2009E+00	1.1242E+01
	Rank	5	3	1	7	2	4	6
F8	Best	2.2225E+03	2.2272E+03	2.2231E+03	2.2285E+03	2.2288E+03	2.2307E+03	2.2320E+03
	Mean	2.2379E+03	2.2302E+03	2.2250E+03	2.2584E+03	2.2334E+03	2.2365E+03	2.2380E+03
	Std	3.5363E+01	1.6912E+00	9.9341E-01	5.3237E+01	2.5095E+00	2.9673E+00	3.3697E+00
	Rank	5	2	1	7	3	4	6
F9	Best	2.4808E+03	2.4808E+03	2.4808E+03	2.4809E+03	2.4811E+03	2.4825E+03	2.4808E+03
	Mean	2.4808E+03	2.4814E+03	2.4812E+03	2.4811E+03	2.4819E+03	2.4836E+03	2.4808E+03
	Std	6.7826E-07	6.0194E-01	3.5524E-01	2.5370E-01	5.1555E-01	6.0588E-01	5.3016E-03
	Rank	1	5	4	3	6	7	2
F10	Best	2.5002E+03	2.5005E+03	2.5004E+03	2.5005E+03	2.5004E+03	2.5006E+03	2.5005E+03
	Mean	2.8539E+03	3.8384E+03	2.5056E+03	2.6403E+03	2.5007E+03	2.5123E+03	2.5008E+03
	Std	5.8060E+02	9.9703E+02	2.7337E+01	6.8881E+02	1.2790E-01	4.2362E+01	1.4307E-01
	Rank	6	7	3	5	1	4	2
F11	Best	2.9000E+03	2.9000E+03	3.0298E+03	2.9206E+03	2.9720E+03	3.2822E+03	2.9102E+03
	Mean	2.9000E+03	2.9005E+03	3.2945E+03	2.9533E+03	3.0363E+03	3.5069E+03	2.9235E+03
	Std	2.6504E-11	5.2304E-01	8.5636E+01	4.0785E+01	4.6826E+01	1.1098E+02	6.2693E+00
	Rank	1	2	6	4	5	7	3
F12	Best	2.9351E+03	2.9421E+03	2.9362E+03	2.9376E+03	2.9394E+03	2.9691E+03	2.9330E+03
	Mean	2.9622E+03	2.9541E+03	2.9443E+03	2.9458E+03	2.9523E+03	2.9832E+03	2.9417E+03
	Std	3.0725E+01	1.0488E+01	4.5961E+00	5.5092E+00	6.8248E+00	1.0156E+01	5.6545E+00
	Rank	6	5	2	3	4	7	1

Table S15. The statistics results of MSEDO and competitors solving constrained engineering optimization

**No.**	Index	MSEDO	EDO	RIME	AE	MRFO	APSM-Jso	IRIME	EMTLBO
CE1	Best	1.2668E-02	1.2692E-02	1.3181E-02	1.3552E-02	1.2780E-02	1.3310E-02	1.2846E-02	1.2908E-02
	Mean	1.2681E-02	2.8988E+04	1.8121E-02	1.6280E-02	1.3870E-02	1.6915E-02	1.5274E-02	1.4412E-02
	Std	1.4231E-05	1.5800E+05	2.2194E-03	1.5071E-03	7.7632E-04	2.2220E-03	2.7399E-03	1.3593E-03
	Rank	1	8	7	5	2	6	4	3
CE2	Best	5.8701E+03	6.2437E+03	5.9598E+03	7.6021E+03	6.3297E+03	7.9456E+03	6.7770E+03	6.6407E+03
	Mean	5.8876E+03	7.7030E+03	7.3437E+03	1.0786E+04	7.0627E+03	1.2331E+04	1.0594E+04	9.6043E+03
	Std	4.2611E+01	1.9944E+03	2.1207E+03	2.1038E+03	5.3569E+02	3.1795E+03	2.1162E+03	1.7634E+03
	Rank	1	4	3	7	2	8	6	5
CE3	Best	2.6389E+02	2.6389E+02	2.6389E+02	2.6389E+02	2.6389E+02	2.6389E+02	2.6396E+02	2.6389E+02
	Mean	2.6389E+02	Inf	2.6463E+02	2.6422E+02	2.6399E+02	2.6453E+02	2.6491E+02	2.6432E+02
	Std	5.5335E-05	NaN	8.6336E-01	3.0395E-01	1.1155E-01	5.1059E-01	7.4696E-01	4.7128E-01
	Rank	1	8	6	3	2	5	7	4
CE4	Best	1.6928E+00	1.7344E+00	1.7197E+00	1.8679E+00	1.7423E+00	1.7869E+00	1.7935E+00	1.7496E+00
	Mean	1.6930E+00	1.8453E+00	1.9938E+00	2.0230E+00	1.8367E+00	1.9441E+00	2.1388E+00	2.0961E+00
	Std	1.0667E-04	1.2522E-01	2.4970E-01	1.2275E-01	7.2188E-02	7.2575E-02	2.0527E-01	5.2775E-01
	Rank	1	3	5	6	2	4	8	7
CE5	Best	2.9936E+03	2.9962E+03	2.9966E+03	2.7953E+03	3.0046E+03	3.0275E+03	3.0034E+03	2.9950E+03
	Mean	2.9936E+03	3.0030E+03	3.0091E+03	3.0307E+03	3.0149E+03	3.0464E+03	3.0150E+03	3.0030E+03
	Std	6.8770E-03	3.9793E+00	1.3163E+01	5.9620E+01	6.5711E+00	1.8564E+01	9.4815E+00	4.4280E+00
	Rank	1	2	4	7	5	8	6	3
CE6	Best	2.7009E-12	9.9216E-10	2.3078E-11	2.3078E-11	2.7009E-12	2.7009E-12	2.3078E-11	2.7009E-12
	Mean	2.3828E-09	2.4409E-02	4.5599E-09	2.7860E-09	9.5034E-10	4.5379E-09	3.8902E-08	5.6213E-09
	Std	5.7109E-09	1.3369E-01	6.6495E-09	3.2762E-09	9.0318E-10	7.2891E-09	1.6199E-07	8.7759E-09
	Rank	2	8	5	3	1	4	7	6
CE7	Best	-2.4358E+05	-2.4358E+05	-2.4358E+05	-2.0665E+05	-2.4145E+05	-2.4295E+05	-2.4358E+05	-2.4358E+05
	Mean	-2.4358E+05	-2.4353E+05	-2.4227E+05	-3.3497E+04	-2.3715E+05	-2.3792E+05	-2.4343E+05	-2.4358E+05
	Std	1.8065E-01	8.7865E+01	2.7198E+03	6.5500E+04	3.7512E+03	3.4820E+03	3.9033E+02	7.6725E-01
	Rank	1	3	5	8	7	6	4	2
CE8	Best	1.3400E+00	1.3502E+00	1.3455E+00	1.4007E+00	1.3410E+00	1.4286E+00	1.3712E+00	1.3777E+00
	Mean	1.3400E+00	5.2083E+20	1.4055E+00	1.5947E+00	1.3505E+00	1.6081E+00	1.7281E+00	1.6974E+00
	Std	6.6771E-08	2.8527E+21	4.6013E-02	9.8418E-02	6.1893E-03	1.2712E-01	1.8028E-01	2.3529E-01
	Rank	1	8	3	4	2	5	7	6.0000E+00
CE9	Best	3.9247E+08	3.9247E+08	3.9247E+08	2.5187E+08	3.9247E+08	3.9247E+08	3.9247E+08	3.9247E+08
	Mean	3.9247E+08	3.9247E+08	3.9247E+08	2.5328E+08	3.9247E+08	3.9247E+08	3.9247E+08	3.9247E+08
	Std	1.8187E-07	1.8187E-07	1.8187E-07	3.7425E+06	5.3206E+02	6.9831E+03	1.8187E-07	1.8187E-07
	Rank	2	2	2	1	7	8	2	2
CE10	Best	1.6086E+01	1.6549E+01	1.6333E+01	2.0652E+01	1.6717E+01	1.7178E+01	1.7220E+01	1.6732E+01
	Mean	1.6086E+01	8.3410E+10	1.7258E+01	1.4850E+02	1.8393E+01	2.9160E+01	2.2468E+01	3.2971E+01
	Std	8.4814E-06	3.1743E+11	5.1070E-01	1.0528E+02	2.0205E+00	1.3542E+01	6.6557E+00	6.9061E+01
	Rank	1	8	2	7	3	5	4	6
